# Curcumin Derivatives Verify the Essentiality of ROS Upregulation in Tumor Suppression

**DOI:** 10.3390/molecules24224067

**Published:** 2019-11-10

**Authors:** Ikuko Nakamae, Tsumoru Morimoto, Hiroki Shima, Masafumi Shionyu, Hisayo Fujiki, Noriko Yoneda-Kato, Takashi Yokoyama, Shigehiko Kanaya, Kiyomi Kakiuchi, Tsuyoshi Shirai, Edy Meiyanto, Jun-ya Kato

**Affiliations:** 1 Laboratory of Tumor Cell Biology, Division of Biological Science, Graduate School of Science and Technology, Nara Institute of Science and Technology, Nara 630-0192, Ikoma, Japan; ikuko-n@bs.naist.jp (I.N.); noriko-k@bs.naist.jp (N.Y.-K.); yokoyama-t@bs.naist.jp (T.Y.); 2 Laboratory of Synthetic Organic Chemistry, Division of Materials Science, Graduate School of Science and Technology, Nara Institute of Science and Technology, Nara 630-0192, Ikoma, Japan; morimoto@ms.naist.jp (T.M.); shima.hiroki.sz2@ms.naist.jp (H.S.); f-hisayo@ad.naist.jp (H.F.); kakiuchi@ms.naist.jp (K.K.); 3 Nagahama Institute of Bio-Science and Technology, Nagahama 526-0829, Shiga, Japan; m_shionyu@nagahama-i-bio.ac.jp (M.S.); t_shirai@nagahama-i-bio.ac.jp (T.S.); 4 Laboratory of Computational Systems Biology, Division of Information Science, Graduate School of Science and Technology, Nara Institute of Science and Technology, Nara 630-0192, Ikoma, Japan; skanaya@gtc.naist.jp; 5 Cancer Chemoprevention Research Center, Faculty of Pharmacy, Universitas Gadjah Mada, Sekip Utara, Yogyakarta 55281, Indonesia; edy_meiyanto@ugm.ac.id

**Keywords:** curcumin, cancer, ROS, tumorigenicity, apoptosis, senescence, ROS metabolic enzymes

## Abstract

Background: Curcumin has been shown to exert pleiotropic biological effects, including anti-tumorigenic activity. We previously showed that curcumin controls reactive oxygen species (ROS) levels through the ROS metabolic enzymes, to prevent tumor cell growth. In this study, we synthesized 39 novel curcumin derivatives and examined their anti-proliferative and anti-tumorigenic properties. Methods and Results: Thirty-nine derivatives exhibited anti-proliferative activity toward human cancer cell lines, including CML-derived K562 leukemic cells, in a manner sensitive to an antioxidant, *N*-acetyl-cysteine (NAC). Some compounds exhibited lower GI_50_ values than curcumin, some efficiently induced cell senescence, and others markedly increased ROS levels, efficiently induced cell death and suppressed tumor formation in a xenograft mouse model, without any detectable side effects. A clustering analysis of the selected compounds and their measurement variables revealed that anti-tumorigenic activity was most well-correlated with an increase in ROS levels. Pulldown assays and a molecular docking analysis showed that curcumin derivatives competed with co-enzymes to bind to the respective ROS metabolic enzymes and inhibited their enzymatic activities. Conclusions: The analysis of novel curcumin derivatives established the importance of ROS upregulation in suppression of tumorigenesis, and these compounds are potentially useful for the development of an anti-cancer drug with few side effects.

## 1. Introduction

Reactive oxygen species (ROS) are highly reactive molecules derived from oxygen (O_2_) and were previously thought to damage cells with few physiological functions. However, recent findings have shown that ROS act as secondary messengers in cellular signaling and participate in cell proliferation, cell survival, differentiation, protein synthesis, glucose metabolism, and inflammation [1,2,3,4]. Most intercellular ROS are generated in the mitochondria, as a byproduct of oxidative phosphorylation. First, superoxide (O_2_^−^⋅) is generated at complexes I and III, then it is converted to hydrogen peroxide (H_2_O_2_) by superoxide dismutases (SODs) [5]. Recent reports have shown that, in addition to doing so through mitochondria, growth factors and cytokines stimulate the production of superoxide by the activation of NADPH oxidase (NOX) in the cytoplasm [6], followed by conversion to H_2_O_2_ by cytoplasmic SOD. H_2_O_2_ is highly diffusible and moderate levels of H_2_O_2_ can act as a bona fide second messenger. However, too-high levels of ROS damage cellular DNA, proteins and lipids, resulting in cell death [1,2,3,4].

In many human cancer cells, ROS levels are elevated, compared to those in their normal counterparts, which benefits many aspects of tumor development and progression. A moderate increase in ROS levels stimulates the MAPK/Erk1/2, PI3K/Akt, and IKK/NF-κB pathways [6,7,8,9], which accelerates cell-cycle progression by upregulating the mRNA levels of cyclins [10], actively promotes cell survival through protein kinase D1 (PKD1) and Akt kinase [11], and positively affects many other factors, such as energy metabolism, cell morphology, cell–cell adhesion, cell motility, angiogenesis, and tumor stemness. However, too-high levels of ROS provoke oxidative stress response and ultimately induce cell-cycle arrest, cell senescence, and apoptosis [1,2,3,4], and, therefore, cancer cells have developed mechanisms to control ROS levels below a certain threshold. Generally, intracellular ROS levels are tightly regulated by non-enzymatic molecules (glutathione (GSH), flavonoids and vitamins A, C, and E) and antioxidant enzymes (SOD, catalase, peroxiredoxin, thioredoxin, glutathione reductase, glutathione peroxidase, and glutathione S-transferase, etc.), depending on the type of ROS [2,3]. GSH is reported to be required for cancer initiation, while the thioredoxin pathway plays a critical role in the later stages of tumor progression [12]. Furthermore, several detoxification enzymes (carbonyl reductase (CBR), glutathione-S-transferase (GST), aldo–keto reductase (AKR), glyoxalase (GLO), NAD(P)H dehydrogenase [quinone] (NQO), peroxiredoxin (PRDX), alcohol dehydrogenase (ADH), etc.) are overexpressed in human tumors [13] and play a critical role in the ROS metabolic pathway [14] and tumor development [15,16,17,18,19,20].

Since elevated levels of ROS endow proliferative advantages to tumor cells, antioxidant reagents were developed as an anti-cancer drug. However, it is difficult to consistently maintain ROS levels at a low level in tumor cells, and, therefore, these drugs had no effect or even worsened symptoms [21,22]. On the other hand, another strategy, to increase ROS levels over the threshold to induce senescence and apoptosis has been considered [3], and methods and reagents have been searched for [12,23,24]. We previously showed that curcumin, a phytopolyphenol that is mainly found in turmeric (*Curcuma longa*), suppressed leukemic cell growth, by increasing ROS levels through targeting ROS metabolic enzymes that were overexpressed in leukemia cells [13]. Curcumin exhibits anti-cancer activity on various cancer cells, and appears to have an anti-proliferative effect on a wide variety of tumor cells, but not on normal cells [25,26,27], suggesting that curcumin is suitable for an anti-cancer drug for humans. To date, 17 recent clinical trials reported efficacy, while another 27 clinical trials and five animal studies indicated therapeutic benefits [28]. In addition, a myeloma patient was reported to remain stable with a daily dietary supplement of curcumin [29]. Thus, it seems that the anti-tumorigenic activity of curcumin warrants further investigation. However, the precise molecular function of curcumin in tumor suppression remains to be elucidated, and the dose of curcumin required for human use is relatively high (ca 8 g/day). These issues should be clarified and improved upon for better use of curcumin for humans.

In this study, we designed and tested novel curcumin derivatives, that had never been reported on before. All suppressed tumor cell growth and some exhibited better qualities than curcumin. A cluster analysis revealed that an increase in ROS was connected with anti-tumorigenic activity. The bioinformatic analysis, supported by the wet experimental data, proposed a model in which curcumin/curcumin derivatives inhibited ROS metabolic enzymes by competing with co-enzymes. These results may provide a clue for better understanding of curcumin’s actions and help methods to develop novel drugs.

## 2. Results

### 2.1. Design and Synthesis of Novel Curcumin Derivatives

To explore the essential properties of curcumin that are associated with anti-tumorigenic activity, in order to understand the precise molecular action of curcumin on inhibiting ROS scavengers, and to improve the availability of curcumin, we designed and synthesized novel curcumin derivatives and analyzed their activities. In this research, we used C7-curcuminoids, which has the same framework as curcumin, and C5-curcuminoids, in which an ethylene group was eliminated from the framework of curcumin, expecting an improvement in water solubility. Since these C5-curcuminoids are asymmetric and have not been previously reported on, we decided to explore the possibility of high activity in these compounds.

**Figure 1:**
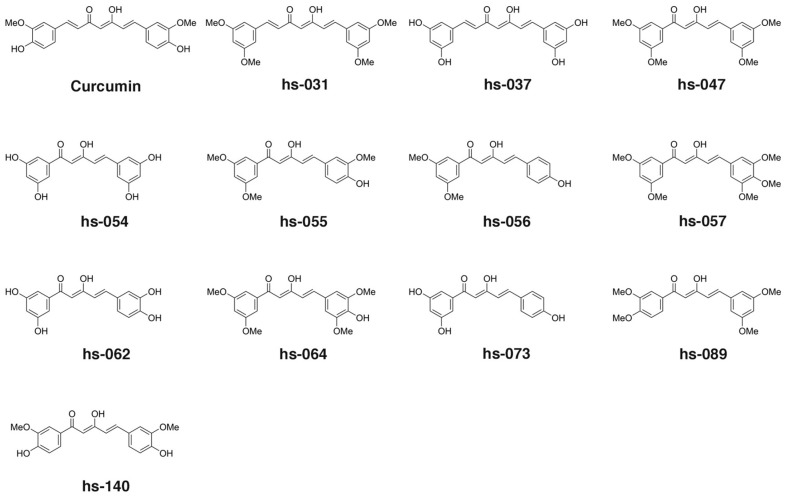
Chemical structure of curcumin and curcumin derivatives used in this study.

First, we designed a C7-curcuminoid hs-037, and C5-curcuminoids h-054, hs-062, and hs-073 (Figure 1), which are expected to show high water solubility from the viewpoint of Clog P values, which were calculated with the ChemDraw 16.0, (hs-037 1.907; hs-054 1.806; hs-062 1.876; hs-073 2.473; curcumin 2.251). In addition, their synthetic precursors were also evaluated (hs-031 for hs-037; hs-047 for hs-054; hs-055 for hs-062; hs-056 for hs-073). Since not many asymmetric C5-curcuminoids among curcumin derivatives or analogs [30,31] have been investigated and reported on before, we investigated the function of C5-curcuminoids that have plural *O*-functionalities (-OMe group) on both aromatic rings (Figure 1, Supplementary Figure S1A) in terms of antitumorigenic activity and inhibition of ROS metabolic enzymes. Finally, as curcumin and PGV-1 [32,33,34,35,36] can affect the activity of ROS metabolic enzymes, C5-curcuminoids hs-140 and hs-157 were designed, synthesized and examined (Figure 1 and Figure S1A).

C7-curcuminoids, hs-031 and its demethylated hs-037, were synthesized via aldol condensation of 2,4-pentanedione with 3,5-dimethoxybenzaldehyde (Figure S1B a) [37]. All the C5-curucuminoids were synthesized via the condensation of 1-aryl-1,3-butanediones, which were prepared by the Claisen condensation of the corresponding acetophenone derivatives with ethyl acetate, [38] with aromatic aldehydes (Figure S1B b) [31].

### 2.2. Growth Inhibition of Human Tumor Cells and ROS Induction by Novel Curcumin Derivatives

**Figure 2:**
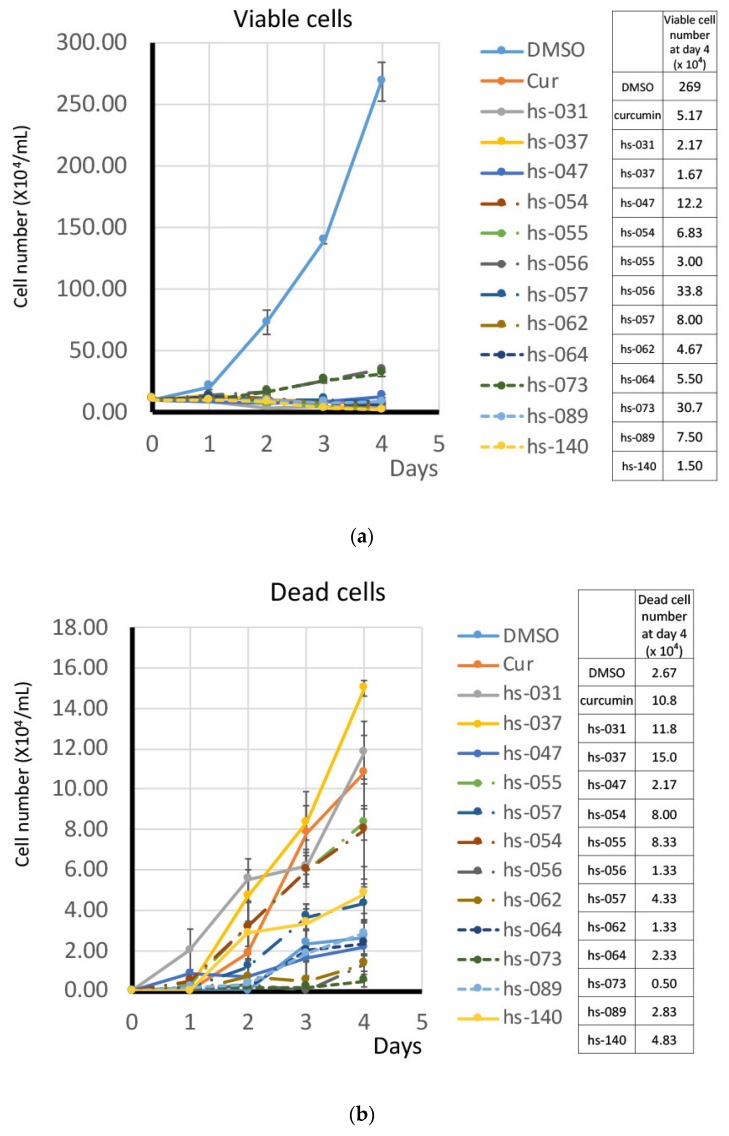

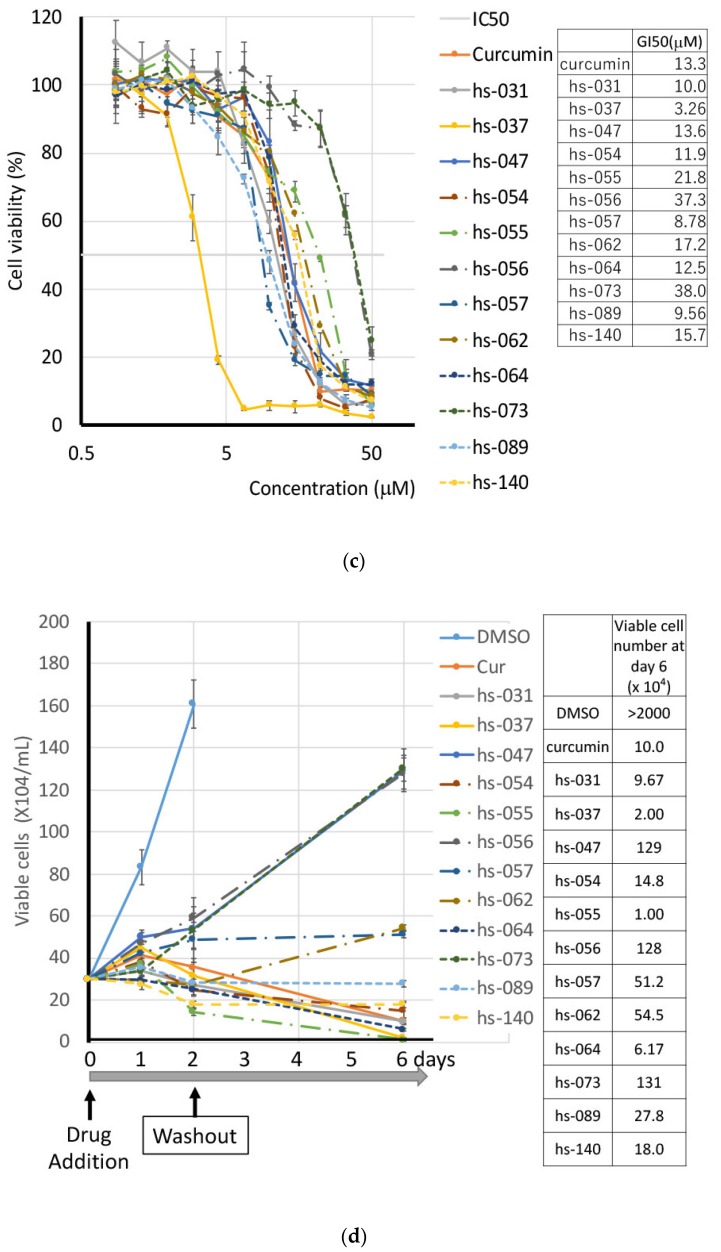
Growth inhibition by curcumin derivatives. (**a**,**b**) K562 cells (10^5^ cells/mL) were cultured in the presence of each compound (50 μM) for 4 days. Cells were enumerated every day using the trypan blue staining method. The results for viable (**a**) and dead (**b**) cells are shown. (**c**) The GI_50_ of curcumin derivatives on K562 cells was determined after a 4-day culture. (**d**) K562 cells (3 × 10^5^ cells/mL) were cultured in the presence of each compound for 2 days, washed once and transferred to the fresh medium without either compound. Viable cells were counted by the trypan blue exclusion method.

To investigate the anti-proliferative activity of novel curcumin derivatives on human tumor cells, we cultured K562 cells in the absence and presence of the representative compounds (50 μM) in vitro. Viable and dead cells were determined by the trypan-blue exclusion assay and enumerated for 4 days (Figure 2a,b). We found that all the compounds showed growth inhibitory activity, but in different degrees, and the induction of dead cells varied from one compound to another. We next determined and compared the GI_50_ of all curcumin derivatives using K562 cells (Figure 2c and Figure S2A, Table S1). The GI_50_ of curcumin derivatives ranged from 3.26 to 101, showing their different levels of effect. Since the GI_50_ of curcumin was calculated as 13.3, 19 compounds (hs-031, 037, 054, 057, 064, 089, 131, 135, 141, 142, 145, 157, 159, 160, 161, 162, 163, and HF-010, 017) exhibited a GI_50_ lower than that of curcumin in this assay.

Since we previously reported that the growth inhibitory effect of curcumin is irreversible [13], we examined the effects of representative curcumin derivatives after their removal from the medium. For this purpose, we cultured K562 cells in the presence of curcumin derivatives for 2 days. After washing, cells were maintained in fresh medium without any drugs for up to six days, and viable cells were counted. Figure 2d shows that growth inhibition by some derivatives was reversible (hs-047, -056 and -073), or partially reversible (hs-057 and 062), whereas cells treated with other compounds (hs-031, 037, 054, 055, 064, 089, and 140) remained growth-inhibited and gradually lost viability even in the absence of the drug.

The growth inhibitory effect of curcumin derivatives was not restricted to K562 leukemic cells. Other types of human cancer cell lines, including U-87 MG glioblastoma, HeLa cervical cancer, MCF-7 breast adenocarcinoma, AN3CA uterine cancer, MIA PaCa-2 and PANC-1 pancreatic cancer, and 293T human embryonic kidney cells, were sensitive to the inhibition of growth by curcumin derivatives (Figure S2B).

**Figure 3:**
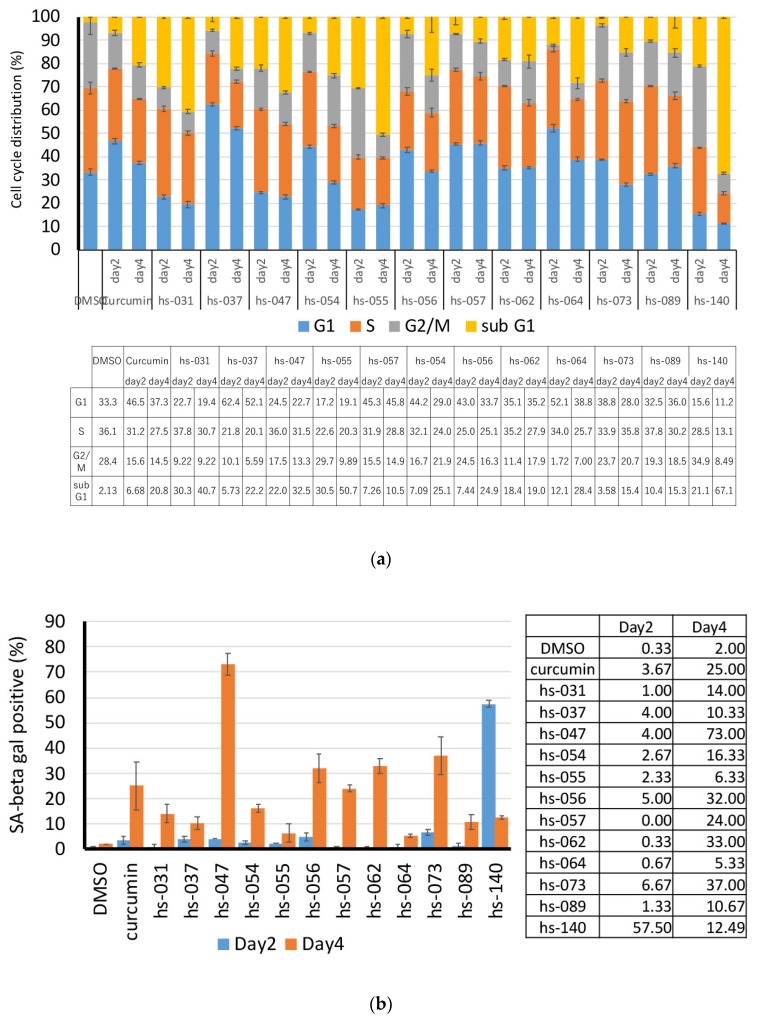

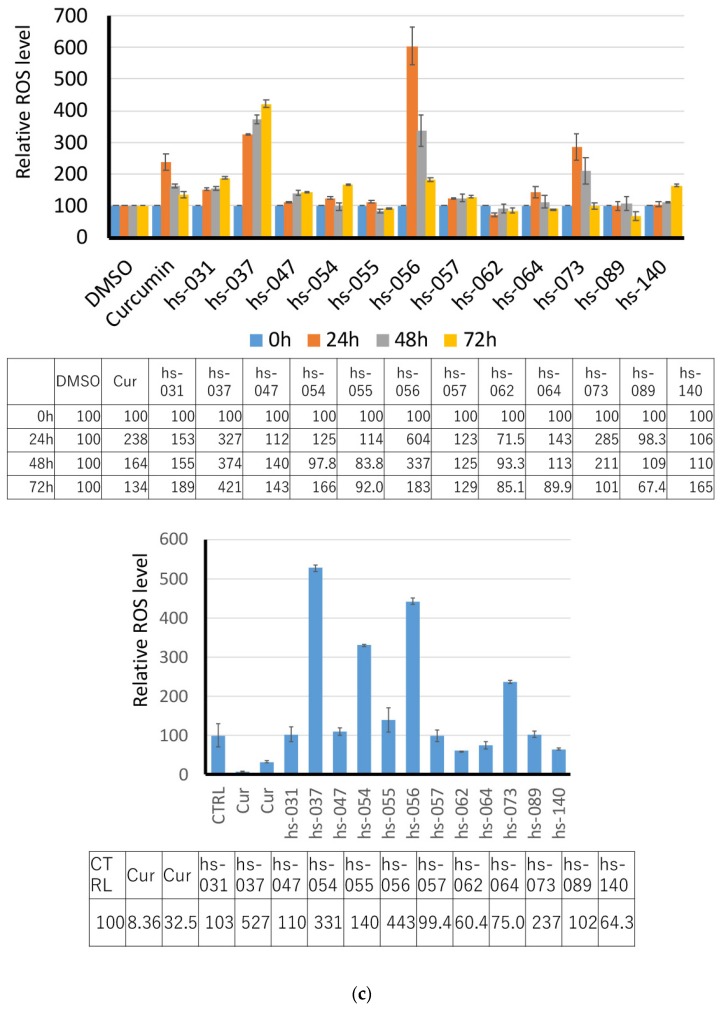

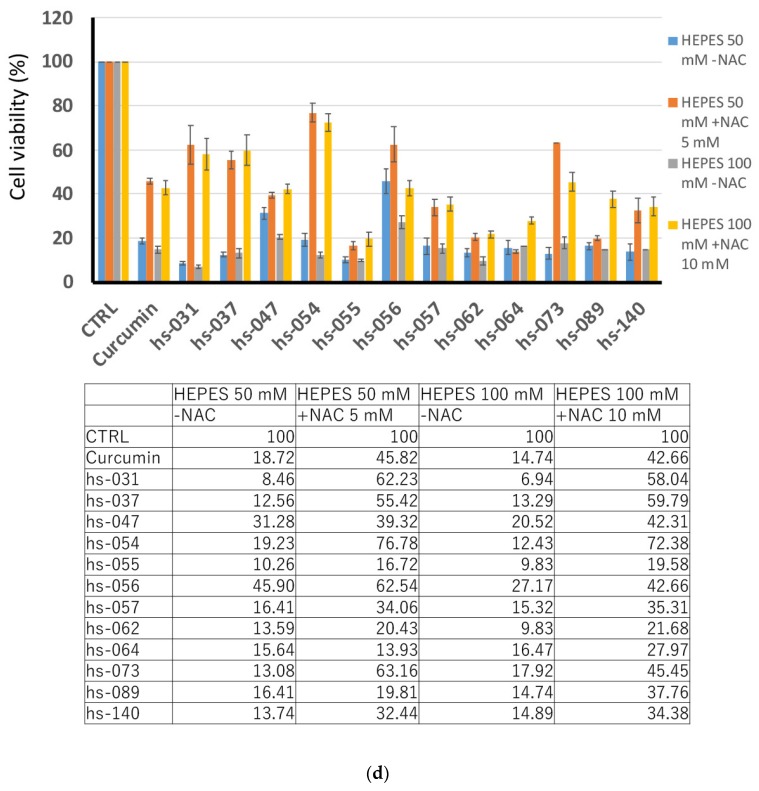
Growth inhibitory properties of curcumin derivatives. (**a**) K562 cells (5 × 10^5^ cells/mL) were treated with curcumin derivatives (50 μM) for 2 and 4 days, and subjected to the cell cycle analysis. (**b**) Cells treated as in (**a**) were subjected to SA-β-gal staining. (**c**) Cells treated with curcumin derivatives (50 μM) for 1 h (lower panel), 24, 48, and 72 h (upper panel) were subjected to a ROS detection analysis using FACS. (**d**) Cells (3 × 10^5^ cells/mL) were cultured in the presence of each compound (50 μM) with and without NAC (5 mM and 10 mM) for 2 days, and viable cells were counted using the trypan blue staining method. HEPES buffer was supplemented to avoid a change in pH. (**a**–**d**) The results are the averages of three independent experiments (means ± SD).

In order to elucidate the anti-proliferative activity of curcumin derivatives, K562 cells were treated with curcumin derivatives for 2 to 4 days and subjected to cell cycle analysis. Figure 3a shows that curcumin and curcumin derivatives did not block the progression of the cell cycle at one exclusive time point, but, rather, the compounds seemed to affect multiple points of the cell cycle, although some were relatively specific to G1 (hs-037) or G2/M (hs-140). However, an increase in the sub G1 population appeared to be common to all compounds, which implies that the compounds induced apoptotic cell death in tumor cells, consistent with the result in Figure 2b. The population of sub G1 increased on day 4 compared to day 2, indicating that consecutive action of the drug effectively induced cell death.

Since cell proliferation was blocked (Figure 2a), but not all cells were dead (Figure 3a), in the presence of the drugs, we suspected that cells may undergo senescence. To examine this possibility, we treated K562 cells with curcumin derivatives for 2–4 days and tested for senescence-associated (SA)-β-galactosidase (gal) activity, a marker of senescence. Figure 3b shows that cells treated with the compounds were positive for SA-β-gal activity. Taking into consideration that cells did not proliferate after removal of the drug from the medium (Figure 2d), we concluded that cells treated with curcumin derivatives underwent senescence, but with a different degree of induction. Thus, the treatment with curcumin derivatives induced both cell death and senescence, which may benefit the efficient inhibition of tumor cell growth.

Since we previously showed that curcumin interacts with several ROS metabolic enzymes and up-regulates intracellular ROS levels in human tumor cells [13], we examined whether treatment with curcumin derivatives would elevate the intracellular levels of reactive oxygen species (ROS). For this purpose, K562 cells were treated with representative curcumin derivatives for 24, 48, and 72 h (Figure 3c upper panel), or for 1 h (Figure 3c lower panel), and ROS levels were measured by staining with fluorescent ROS sensors, followed by an FACS analysis. Figure 3c shows that some compounds significantly increased their ROS levels 24–72 h after addition. Some compounds, such as hs-037, 056, and 073, increased ROS levels at a markedly higher rate than curcumin, and their effect started 1 h after the addition of the compounds. Interestingly, curcumin treatment temporarily decreased ROS levels 1 h after its addition, and we do not know what caused this. Some compounds, such as hs-054, immediately increased ROS levels 1 h after its addition, but ROS levels returned to their normal level after 24 h.

To investigate the significance of the increase in ROS levels by curcumin derivatives for tumor cell growth, we examined the effect of curcumin derivatives in the presence of the antioxidant NAC on the proliferation/survival of human tumor cells. K562 cells were treated with curcumin derivatives in the presence and absence of NAC. We added HEPES buffer to the medium to avoid a change in the pH of the medium by NAC. After 4 days, viable cells were counted in the presence of trypan blue. Figure 3d shows that NAC counteracted the growth inhibitory effect of curcumin derivatives. The degree of restoration differed from one compound to another, but, basically, the treatment with all compounds was sensitive to the action of NAC, suggesting that even compounds in which we failed to detect much increase in ROS levels suppressed tumor cell growth, at least in part, through the upregulation of ROS levels. For example, treatment with the compounds that increased ROS levels to much higher than those in curcumin (hs-037, 056, and 073) markedly recovered cell viability in the presence of NAC, indicating that these compounds act through the upregulation of ROS levels. Notably, the inhibitory effect of the compound represented by hs-031 was also markedly recovered, even though we detected minimum elevation of ROS levels 1, 24, 48, and 72 h after addition of the compound to the medium.

### 2.3. Anti-Tumorigenic Activity of Novel Curcumin Derivatives

**Figure 4:**
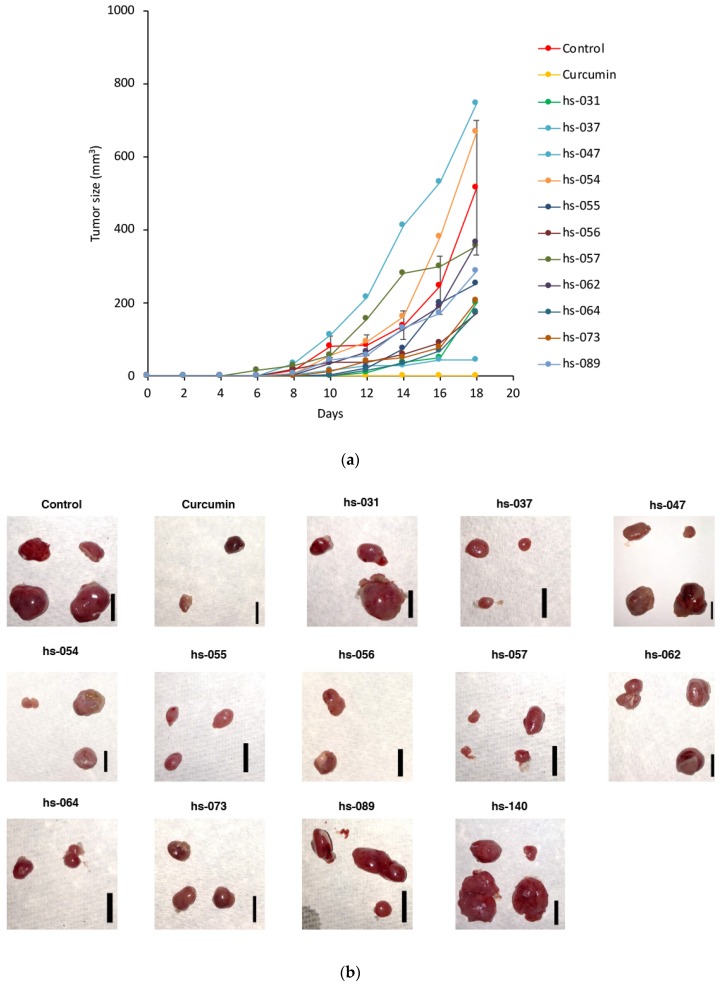

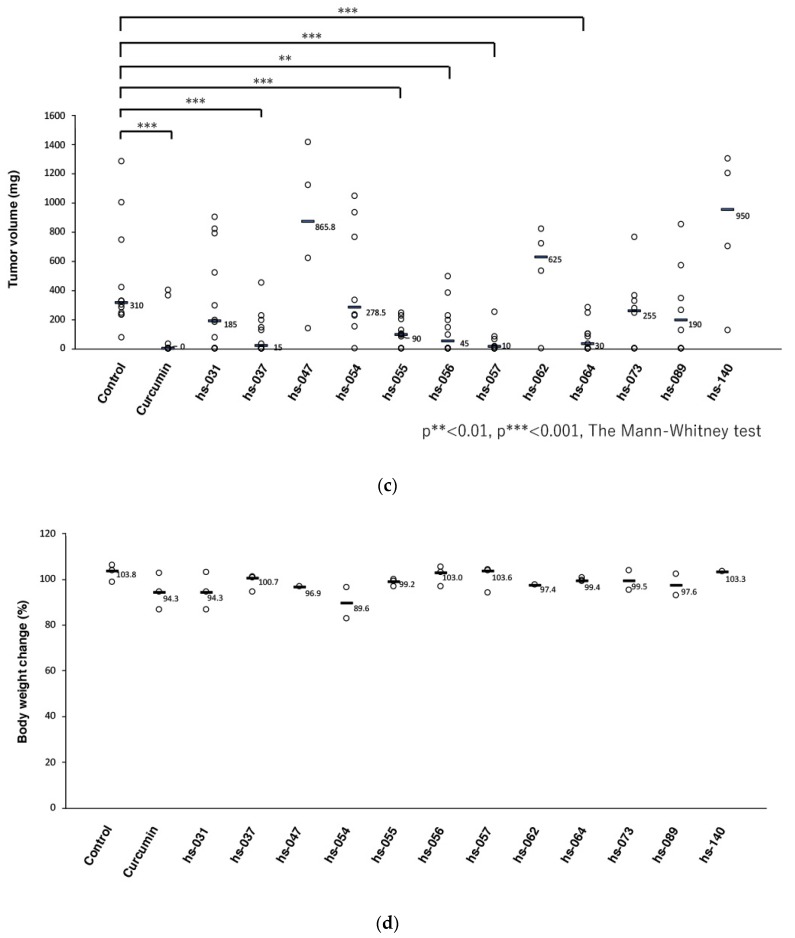
Inhibition of tumor formation by curcumin derivatives. (**a**) K562 cells (2.5 × 10^6^ cells) were transplanted s.c. into the flanks of nude mice. Mice were then treated with curcumin derivatives (25 mg/kg BW) in corn oil and vehicle (corn oil) via an i.p. injection every 2 days. The tumor sizes were measured every 2 days. (**b**–**d**) After 18 days post-injection, mice were sacrificed, tumors were taken (**b**), and tumor weights (**c**) and the body weights (**d**) were measured.

Since we previously reported that curcumin suppressed the tumorigenic cell growth of human cancer cells in a xenograft mouse model [13], we tested curcumin derivatives for their anti-tumorigenic activity in a xenograft system. K562 cells were subcutaneously (s.c.) injected into the flanks of nude mice, and curcumin derivatives dissolved in corn oil were administered to these mice via intraperitoneal (i.p.) injection every 2 days (Figure 4a). As a control, corn oil alone was given. After 18 days, visible tumors had formed at all injection sites in control mice, and treatment with curcumin suppressed the formation of tumors (Figure 4b–c). Among curcumin derivatives, hs-037, 055, 056, 057, and 064 markedly reduced the size of tumors (Figure 4b–c, Table S1).

Notably, mice treated with curcumin and curcumin derivatives showed no decrease in body weight (Figure 4d), nor a significant decrease in white (Figure S2C a) and red (Figure S2C b) blood cell counts and platelet counts (Figure S2C c) in their peripheral blood, nor any other adverse effects in behavior and macroscopic appearance. Thus, curcumin and curcumin derivatives did not induce any obvious adverse effects in the normal lineage of cells under the conditions in which curcumin and a group of curcumin derivatives sufficiently inhibit tumor cell growth in vivo.

### 2.4. Clustering Analysis of Properties of Novel Curcumin Derivatives

**Figure 5:**
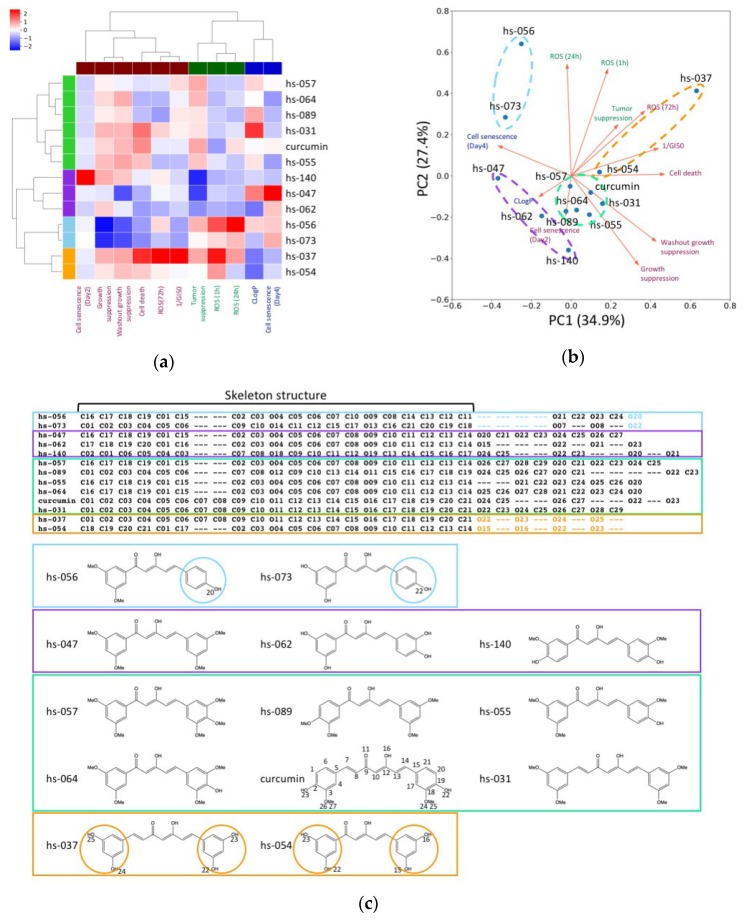
Cluster analysis. (**a**) Heatmap of normalized 11 measurement variables obtained from 13 curcumin derivatives. The dendrograms on the top and left of the heatmap indicate the hierarchical clustering of the measurement variables and derivatives, respectively. The clustering analyses were performed by using complete linkage method with distance matrix converted from the correlation matrix of the normalized measurement variables. A threshold of 0.7× (maximum value of distance between the clusters) was employed for the clustering. The leaves of the dendrograms are colored according to the clusters. (**b**) PCA biplot of measurement variables and derivatives. The red arrows are projections of the measurement variables onto the first two principal components. The names of the measurement variables are colored according to the clusters in plate a. The derivatives (blue points) classified into the same cluster are surrounded by a dashed circle colored according to the clusters in plate a. (**c**) Comparison of the chemical structures of the derivatives. The atom alignment was generated by the COMPLIG program [39]. The derivatives are surrounded by rectangles colored according to the clusters in plate a. The characteristic moieties of clusters 1 and 4 are highlighted in the alignment and encircled in the formula. The atom numbers are labeled for curcumin and the characteristic moieties for simplicity.

We performed a clustering analysis of the curcumin derivatives and their measurement variables in order to evaluate the relationship among parameters. We selected and analyzed 11 variables, which include (1) CLogP, (2) growth suppression, (3) cell death, (4) 1/GI50, (5) washout growth suppression, (6) cell senescence (Day 2), (7) cell senescence (Day 4), (8) ROS (1 h), (9) ROS (24 h), (10) ROS (72 h), and (11) tumor suppression (Table 1 and Table S1, Figure 3b,c). The clustering analysis indicated that 11 measurement variables were classified into three clusters: cluster 1 (consisting of cell senescence (Day 2), growth suppression, washout growth suppression, cell death, ROS (72 h), and 1/GI50), cluster 2 (tumor suppression, ROS (1h), and ROS (24 h)), and cluster 3 (CLogP and cell senescence (Day 4)) (Figure 5a). The curcumin and 12 derivatives were classified into four clusters: cluster 1 (hs-057, hs-064, hs-089, hs-031, curcumin, and hs-055), cluster 2 (hs-140, hs-047, and hs-062), cluster 3 (hs-056 and hs-073), and cluster 4 (hs-037 and hs-054) (Figure 5a).

The PCA (Principal Component Analysis) analysis showed that the variables related to cell number suppression, such as cell death and 1/GI50, and cell senescence (Day 4), mainly contributed to the first principal component (PC1) (Figure 5b). Interestingly, cell death and cell senescence (Day 4) conversely contributed to PC1. The second principal component (PC2) was mainly determined by the variables relevant to ROS activation, such as ROS (1 h) and ROS (24 h). In addition, the clustering analysis showed that the curcumin derivatives in clusters 3 and 4, especially hs-056 in cluster 3 and hs-037 in cluster 4, had distinguished profiles from curcumin. The derivatives hs-056 and hs-037 demonstrated relatively higher tumor suppression and ROS activation than the other derivatives. However, tumor suppression activity appeared to be positively correlated to cell number suppression and ROS activation in cluster 4, whereas it was correlated to ROS activation and cell senescence (Day 4), rather than cell number suppression, in cluster 3. These results imply that these curcumin derivatives exhibited tumor suppression activity by distinct pathways of ROS activation. According to an atom alignment generated by a graph match algorithm, the derivative clusters 3 and 4 had distinct structural features from the other derivatives: the derivatives in cluster 3 (hs-056 and hs-073) had one phenol ring and the derivatives in cluster 4 (hs-054 and hs-073) had two 1, 3-dihydroxybenzen rings (Figure 5c). These characteristic moieties might have affected the interaction between the derivatives and their potential targets, and generated the difference in ROS activation pathways.

### 2.5. Binding Properties of Novel Curcumin Derivatives to ROS Metabolic Enzymes and Clustering Analysis

**Figure 6:**
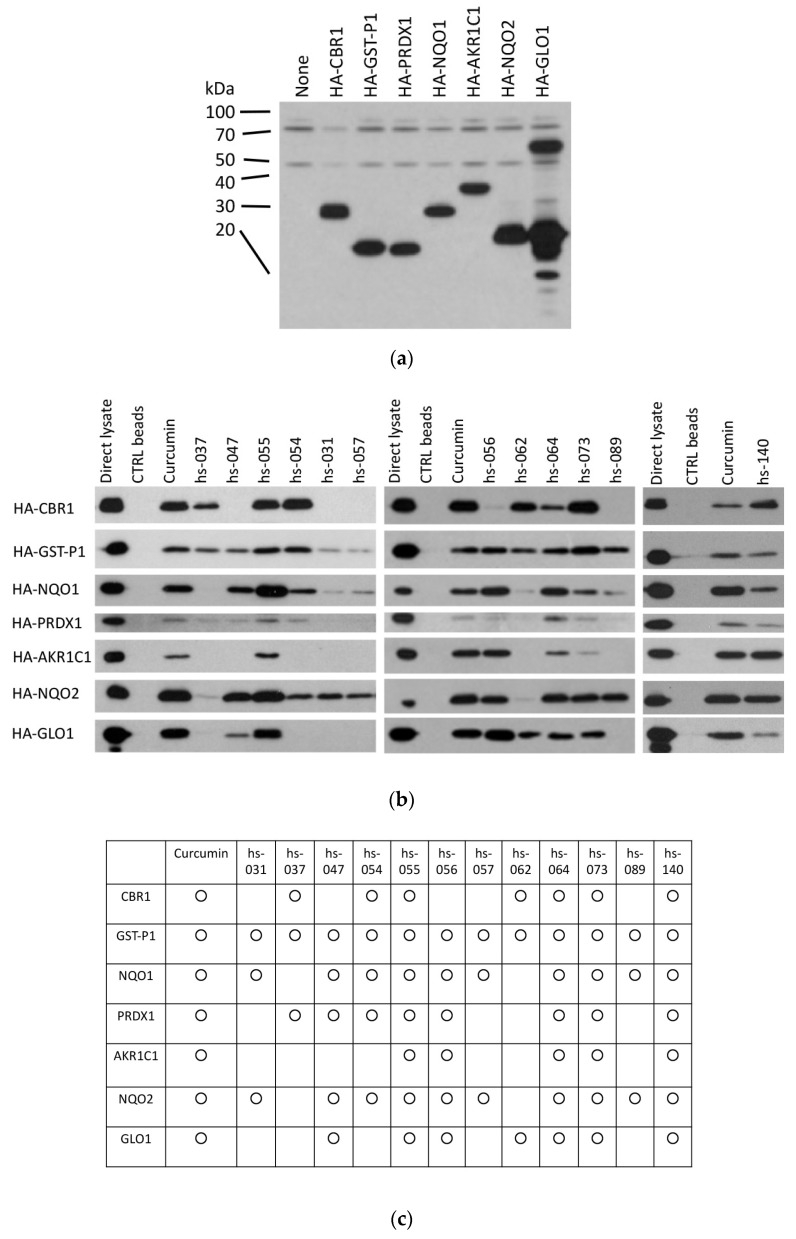

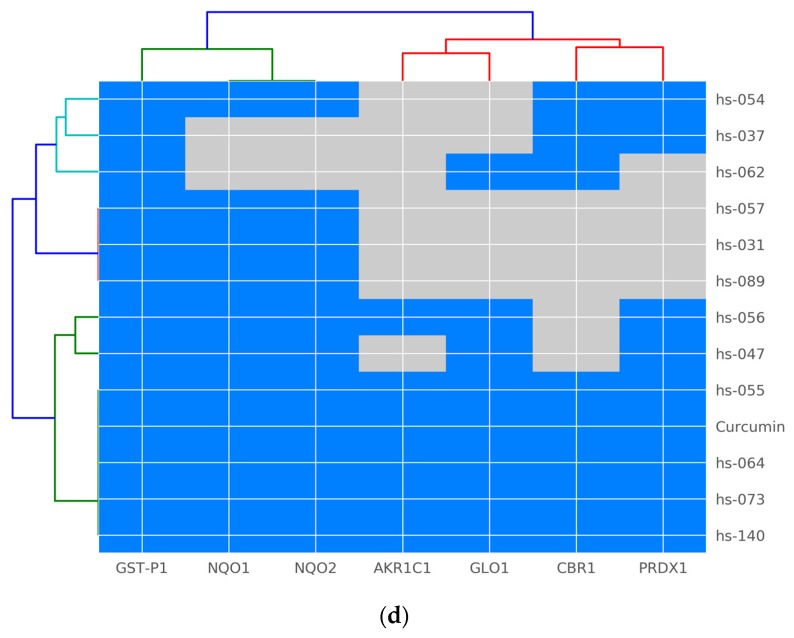
Binding of curcumin derivatives to ROS metabolic enzymes. (**a**,**b**) Control, curcumin, and curcumin derivatives beads were subjected to a pull-down assay using cell lysates containing HA-CBR1, HA-GSTP1, HA-PRDX1, HA-NQO1, HA-AKR1C1, HA-NQO2, and HA-GLO1 proteins (**a**). Bound proteins were visualized by immunoblotting, using an anti-HA antibody (**b**). (**c**) Summary of the results of panel b. The positive combination is marked as O. (**d**) Heatmap of the binding result shown in panel c. The dendrograms on the top and left of the heatmap indicate the hierarchical clustering of the target proteins and derivatives, respectively.

Since we previously showed that curcumin interacts with a group of enzymes functioning in the ROS metabolic pathway [13,14], we tested whether curcumin derivatives interact with these enzymes in vitro (Figure 6a–c). We conjugated curcumin derivatives (hs-031, 037, 047, 054, 055, 056, 057, 062, 064, 073, 089, and 140) on epoxy–sepharose beads and performed a pull-down assay using lysates isolated from cells transfected with HA-tagged enzymes (Figure 6a). The results of the binding (Figure 6b) and the summary of the results (Figure 6c) are shown.

Figure 6c shows that the interaction profile differs from one compound to another. Glutathione-*S*-transferase phi 1 (GST-P1) bound to all the compounds (13 out of 13 compounds) we tested, followed by NAD(P)H dehydrogenase [quinone] 1 and 2 (NQO1 and NQO2) (11 out of 13 compounds), while peroxiredoxin-1 (PRDX1) (9 out of 13 compounds), carbonyl reductase 1 (CBR1) (8 out of 13 compounds), and glyoxalase I (GLO1) (8 out of 13 compounds) were intermediate, and the aldo–keto reductase family 1 member 1 (AKR1C1) (6 out of 13 compounds) bound to the least. The clustering analysis shows that target enzymes and the compounds can be classified into several groups (Figure 6d), but the correlation with anti-proliferative and anti-tumorigenic properties was not significant, suggesting that no single target molecule was responsible for the anti-cancer activity, rather, their combinatory inhibition may be the key property for curcuminoid bioactivity, or, otherwise, other unidentified target molecules may be responsible for the action of the derivatives.

### 2.6. Mode of Inhibitory Action of Novel Curcumin Derivatives on ROS Metabolic Enzymes

**Figure 7:**
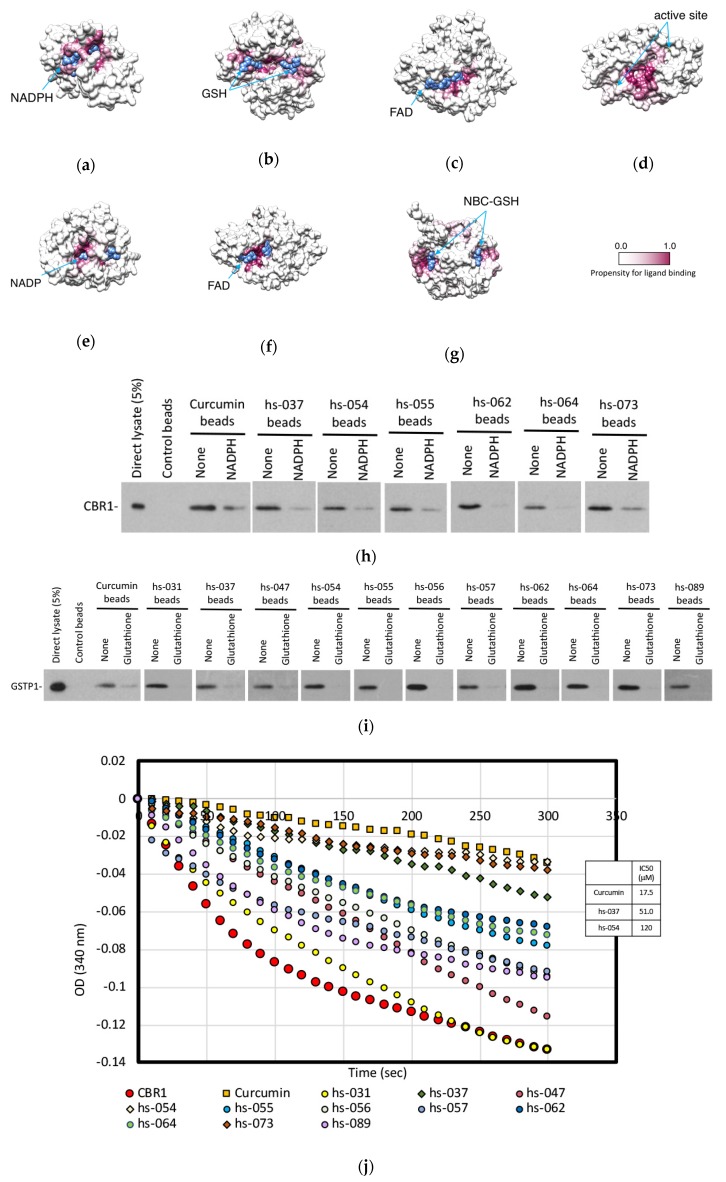

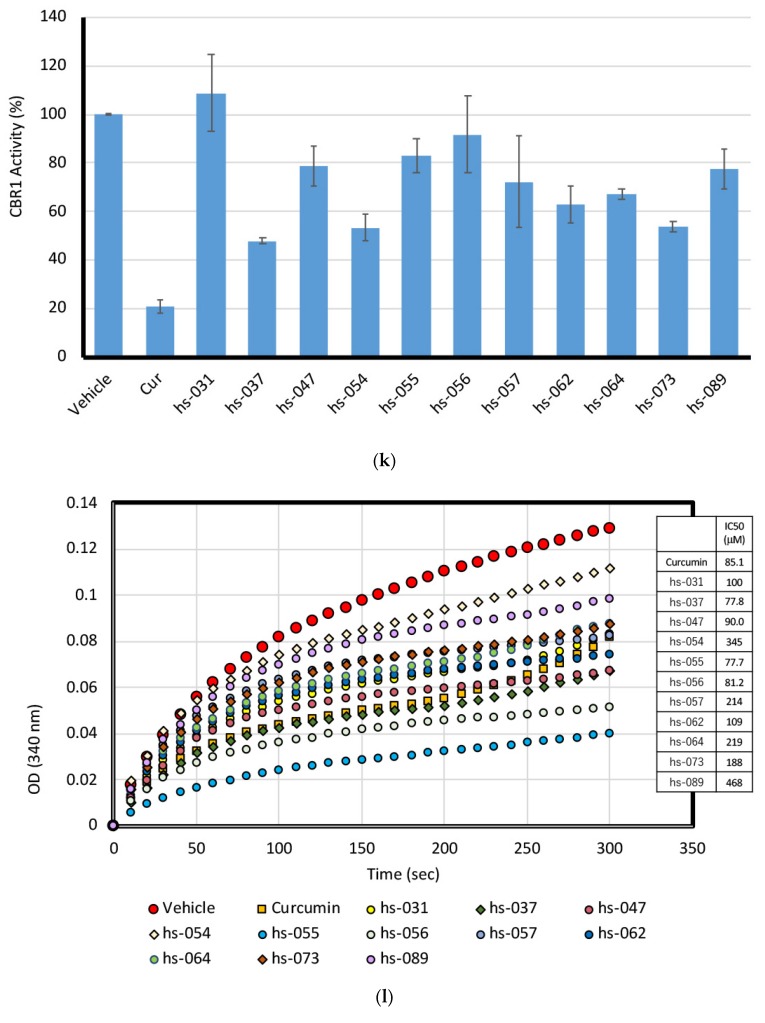

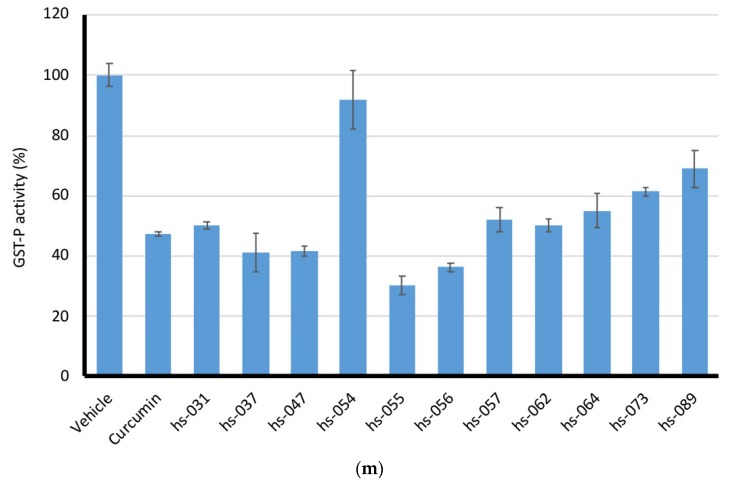
Predicted binding sites of curcumin on ROS metabolic enzymes, (**a**) CBR1, (**b**) GST-P1, (**c**) NQO1, (**d**) PRDX1, (**e**) AKR1C1, (**f**) NQO2, and (**g**) GLO1. The ROS metabolic enzymes are shown in the surface model. The residues are colored by maroon gradation according to their propensity for ligand binding. The bound co-enzymes (NADPH, FAD, and NADP) or substrate/substrate analog (GSH and NBC-GSH) are shown in sphere models colored in blue. (**h**,**i**) Competitive pulldown assay between cell lysates containing HA-CBR1 (**h**)/HA-GSTP1 (**i**) proteins and curcumin derivatives-beads in the presence and absence of 10 mM NADPH (**h**) and 10 mM glutathione (**i**). Bound proteins were visualized by immunoblotting, using an anti-HA antibody. (**j**–**m**) CBR1 (**j**) and GST-P1 (**l**) enzymatic activities was measured in vitro in the presence of curcumin derivatives. The IC_50_ of the compounds is shown as the mean ± SD (*n* = 3). CBR1 (**k**) and GST-P1 (**m**) activities in the presence of curcumin derivatives (50 μM) are shown as the mean ± SD (*n* = 3).

To better understand how curcumin and the curcumin derivatives affect the ROS metabolic enzymes, we predicted the binding sites of the ligands on the enzymes by using molecular docking simulations. The predicted binding sites of curcumin (Figure 7a–g) and the curcumin derivatives (Figure S4) are indicated by the highlighted high-propensity sites for ligand binding. In most of the cases, the probable binding sites are located near the binding sites for co-enzymes, such as NADPH for CBR1, FAD for NQO1 and 2, NADP for AKR1C1, or binding sites for substrate/substrate analog (GSH for GST-P1, NBC-GSH for GLO1), or near the enzymatically active site (PRDX1). These results imply that curcumin and the curcumin derivatives might compete with co-enzyme and/or substrate and attenuate enzyme activities.

To examine whether curcumin derivatives and co-enzymes compete to bind to the enzymes, we performed a competition binding assay using the in vitro pull-down assay between curcumin derivatives and CBR1 or GST-P1, in the absence and presence of NADPH (for CBR1) or GSH (for GST-P1). Figure 7h,i show that binding between CBR1 and the compounds (curcumin, hs-037, -054, -055, -062, -064, and -073) was lowered in the presence of NADPH (Figure 7h), and addition of GSH weakened the interaction between GST-P1 and the derivatives (curcumin, hs-031, -037, -047, -054, -055, -056, -057, -062, -064, -073, and -089) (Figure 7i).

Because curcumin derivatives can compete with co-enzymes to bind to the enzymes, these compounds may inhibit the enzymatic activity of the enzymes. To test this hypothesis, we established the enzymatic activity assay in vitro for CBR1 (Figure 7j) and GST-P1 (Figure 7l) and measured the activity of each enzyme in the presence of curcumin and its derivatives. In these assays, recombinant CBR1 and GST-P1 proteins were expressed in *E. coli* and affinity-purified. Recombinant enzymes were mixed with synthetic substrates in the presence of co-enzymes and the absorbance of the substrates were monitored. CBR1 bound to curcumin, hs-037, -054, -055, -062, -064, and -073 (Figure 6c), among which curcumin, hs-037 and -054 significantly inhibited CBR1 activity under this condition (Figure 7j,k), whereas GST-P1 activity was markedly inhibited by all compounds, except hs-054 (Figure 7l,m).

Thus, these data indicate that curcumin and curcumin derivatives inhibited the enzymatic activity of ROS scavengers by interfering with the binding of co-enzymes to the enzymes, but the specificity and the degree of inhibition varied from one compound to another, generating the complexity of the effectiveness of the compounds.

## 3. Discussion

Curcumin is widely used in Asian cuisine as a spice and in Aver medicine as an herb, and has been reported to exhibit many therapeutic and biological effects, including anti-tumorigenic activity [25,26]. Curcumin has been reported to affect many signaling pathways [26], which has led to the idea that the anti-tumorigenic effect of curcumin may be mediated by several factors, and a combination of distinct biochemical pathways. We recently showed that curcumin directly bound to several ROS scavengers and increased ROS levels in tumor cells, and, most importantly, anti-oxidants GSH and NAC neutralized the anti-proliferative effect of curcumin in tumor cells [13]. These results demonstrate that the anti-tumorigenic effect of curcumin is mediated somewhat, if not entirely, through the upregulation of ROS levels over the threshold in the cell. However, the neutralizing effect of anti-oxidants were partial (Figure 3d, and see [13]), and the clustering analysis (Figure 5) pointed out the involvement of non-ROS pathways, as well as the ROS activation pathway. Curcumin has been well known to target several different factors, such as NFkB, recently-identified target DYRK2 [40], etc. An ROS-mediated pathway may work together with these pathways to suppress tumor cell growth. In this study, we designed a series of molecules related to curcumin and tested their anti-proliferative and anti-tumorigenic activities. The clustering analysis of these curcuminoids and their measurement variables revealed that ROS levels and tumor suppression were classified into the same cluster, indicating the importance of ROS induction in curcuminoid-mediated tumor growth inhibition.

Today, many researchers evaluate ROS for its ability to control tumors in terms of metastasis prevention [41], as well as suppressing growth [3]. Since a reduction in ROS levels in tumor cells has been proven to be of limited efficacy [21,22], a different strategy, to increase ROS levels in tumor cells enough to induce senescence and apoptosis, has been explored, and curcumin seems to be a promising candidate to achieve this [13]. The advantage of curcuminoid application is that these compounds upregulate ROS levels in a temporary manner, and their cytotoxic effect is more selective to tumor cells than to normal cells because of the low basal levels of ROS in normal cells, implying that few side effects are expected for curcuminoid-mediated therapy.

The following question about curcumin remains to be resolved: why this high specificity in such a small molecule? Curcumin has many target molecules [25,26], but still retains specificity. For example, curcumin binds to a variety of ROS metabolic enzymes that require different types of co-enzymes, such as NADPH, FAD, and GSH. In this study, we showed that curcumin and curcumin derivatives competed to bind to the enzymes with their specific co-enzymes. However, we do not see much similarity among curcumin and co-enzymes, suggesting that the action of curcumin does not seem to mimic the structure of co-enzymes. In addition, among the family of proteins, curcumin exhibits specificity. Curcumin binds to and inhibits the enzymatic activity of GST-P1, but does not interact with another member of GST, GST-O1, or GST from other species (*Schistosoma japonicum)*. This result suggests that curcumin does not simply replace the molecular action of GSH as an unreactable co-enzyme, and may have its own specificities for inhibition, which presumably reflects the biological responses mediated by curcumin. Human cells contain nine classes of GST [16], and curcumin acts on only a subset of GST members. It may be interesting to functionally analyze the physiological difference of curcumin-binding GST and non-binding GST in terms of tumorigenesis.

Many proteins and enzymes have been reported to be targets of curcumin [25,26]. In fact, we found that curcumin interacts with and inhibits several enzymes in the ROS metabolic pathway [13]. Strangely, these enzymes do not necessarily belong to the same family, nor do they share structural similarities. In this study, we found that curcumin competes with co-enzymes to bind to the enzyme, which provides a clue into the mechanisms of the molecular action of curcumin to many targets. However, the fact remains that co-factors, such as NADPH, FAD and GSH, do not necessarily share a common structure. Additional, deeper insights will be needed to understand the mechanism of the specificity of curcumin.

Among the C5-curcuminoids generated and examined in this study (Figure 1 and Figure S1), hs-163 and HF-016 scored the lowest and the highest GI_50_, respectively (Table S1). These two compounds were the same molecular weights and harbored the same functional groups (methoxy groups, -OMe), but at different positions (Figure S1). When we compared the molecular structures of these two molecules (Figure S3), we found that the angles formed between two aromatic rings were 14.7° and 23.0°, for hs-163 and HF-016, respectively. Furthermore, the methoxy group at position 2 in HF-016 was out of the plane in the molecule. These observations suggest that the planarity of the whole molecule is critical for the anti-proliferative activity of the curcuminoid.

In the previous and present studies, we revealed a novel molecular pathway (a ROS-mediated pathway), by which curcumin exerts anti-proliferative and anti-tumorigenic activities, and suggested a putative molecular mechanism (competition with co-enzymes) by which curcumin acts on multiple targets. Recently, ROS has been reported to play a critical role in tumor metastasis [41], as well as in tumor growth [3], and the mechanisms of ROS regulation have been paid more attention than ever. In addition, the effect of curcumin is more selective to tumor cells than to normal cells [27], thus, fewer side effects are expected for therapy. Thus, it is worthwhile to continue the research on curcumin and its derivatives, which will lead to the finding of a novel therapy with much lfewer side effects.

## 4. Materials and Methods

### 4.1. Synthesis

#### 4.1.1. C7-Curcuminoids, hs-031 and hs-037

C7-Curcuminoid, hs-031, was synthesized from 2,4-pentanedione as a starting material, using the modification of the method previously reported [37]. The representative procedure is as follows. In a 30 mL two-necked flask 2,4-pentanedione (1.0 mmol), boric anhydride (1.0 mmol), and ethyl acetate (2.0 mL) were placed, and the mixture was stirred at 40 °C for 45 min. A solution of 3,5-dimethoxybenzaldehyde (2.0 mmol) in ethyl acetate (5.5 mL), followed by tributyl borate (4.0 mmol), was added to the reaction mixture at 40 °C, and the mixture was stirred at the same temperature for 45 min. Then, a solution of *n*-butylamine (0.6 mmol) in ethyl acetate (2.0 mL) was added dropwise over a period of 15 min at 40 °C, and the whole mixture was stirred at the same temperature for a further 3 h. After 3 h, the reaction was quenched with 0.4 N HCl aq. (5.0 mL). The organic layer was separated, and the aqueous layer was extracted with ethyl acetate (10 mL × 3). The combined organic extracts were washed with saturated NaCl aq. (5 mL), dried over Na_2_SO_4_, and concentrated in vacuo. The concentrate was dissolved in methanol (5.0 mL) and cooled to −20 °C to obtain a yellow crystal in 47%. The product (hs-031) was identified with ^1^H-NMR, ^13^C-NMR, IR, LRMS, and HRMS.

In a 10 mL two-necked plask, hs-031 (0.25 mmol) and CH_2_Cl_2_ (5 mL) were placed, and the mixture was cooled to −78 °C. To the cooled mixture was added dropwise BBr_3_ (8.0 mmol), and the whole mixture was stirred at the same temperature for 19 h. After the reaction mixture was warmed to 0 °C, 10 mL of saturated NaHCO_3_ aqueous solution was added slowly. The organic layer was separated, and the aqueous layer was extracted with ethyl acetate (30 mL × 3). The combined organic extracts were dried over Na_2_SO_4_ and concentrated in vacuo. The concentrate was purified by column chromatography on silica-gel (eluent; hexane/ethyl acetae) to give hs-037 in 44% yield as a yellow solid.

#### 4.1.2. 1-Aryl-1,3-diketones

Two drops of absolute EtOH, acetophenone derivative (25 mmol) in dry THF (20 mL), 18-crown-6 (0.4 mmol) in THF (20 mL) were added consecutively to the stirred mixture of NaH (50 mmol) and ethyl acetate (50 mmol) in dry THF at room temperature, and then it was refluxed until the reaction was completed. After acetophenone derivative was completely consumed, 10% H_2_SO_4_ was added dropwise to the precooled reaction mixture. The organic layer was separated, and the aqueous layer was extracted with ethyl acetate (50 mL × 3). The combined organic extracts were washed with 10 mL of a saturated NaHCO_3_ aqueous solution, dried over Na_2_SO_4_, and concentrated in vacuo. The concentrate was purified by recrystallization (hexane/toluene).

#### 4.1.3. C5-Curcuminoids

All C5-curcuminoids were synthesized from the corresponding 1-aryl-1,3-diketones as starting materials, using the modification of the method previously reported [31]. The representative procedure is as follows. In a 30 mL two-necked flask 1,3-diketone (2.0 mmol), boric anhydride (2.0 mmol), and ethyl acetate (2.0 mL) were placed, and the mixture was stirred at 40 °C for 45 min. A solution of aldehyde (4.0 mmol) in ethyl acetate (5.5 mL), followed by tributyl borate (8.15 mmol), was added to the reaction mixture at 60 °C, and the mixture was stirred at the same temperature for 45 min. Then, a solution of *n*-butylamine (2.0 mmol) in ethyl acetate (2.0 mL) was added dropwise over a period of 15 min at 60 °C, and the whole mixture was stirred at the same temperature for a further 3 h. After 3 h, the reaction was quenched with 0.4 N HCl aq. (5.0 mL). The organic layer was separated, and the aqueous phase was extracted with ethyl acetate (10 mL × 3). The combined organic extracts were washed with saturated NaCl aq. (5 mL), dried over Na_2_SO_4_, and concentrated in vacuo. The concentrate was dissolved in methanol (5.0 mL) and cooled to −20 °C to obtain a yellow crystal. The product was identified with ^1^H-NMR, ^13^C-NMR, IR, LRMS, and HRMS.

### 4.2. Proliferation Assays

Tissue culture of human cancer cell lines, transfection of plasmid DNA, cell cycle analysis, β-gal assay, and measurement of intracellular ROS levels were performed as described previously [13,42].

### 4.3. Protein Analyses

Construction of expression vectors, immobilization of curcumin derivatives on sepharose beads, a pull-down assay, enzyme assay, and molecular docking analysis were performed as described previously [13,42].

### 4.4. CBR and GST Enzyme Assays

6xHis-tagged CBR1 and GST-P1 recombinant proteins were expressed in bacteria, affinity purified using Ni-NTA agarose beads (QIAGEN), eluted with 0.5 M imidazole, and dialyzed against PBS at 4 °C. Two micrograms of recombinant GST-P1 protein was incubated with curcumin derivatives for 1 h on ice, then mixed with a reaction solution (0.1 M potassium phosphate buffer, pH 6.5) containing 2 mM glutathione-reduced form (GSH) and 1 mM 1-chloro-2,4-dini-trobenzene (CDNB) (Sigma) [43]. To detect GSH-conjugated CDNB, an absorbance of 340 nm was recorded every 10 s for 5 min at 25 °C, with a UV-1800 spectrophotometer (SHIMADZU). The Km and Vmax values were determined from direct plots of velocity versus substrate concentration, and are presented along with their standard errors.

### 4.5. Tumorigenicity Test

K562 cells were subcutaneously (s.c.) injected into nude mice, and curcumin and curcumin derivatives dissolved in corn oil were intra-peritoneally (i.p.) administered to mice (50 μL of 10 mg/mL compounds for 20 g BW), as described previously [13]. For the control, corn oil alone was used. All methods were carried out in accordance with NAIST guidelines and regulations, and all experimental protocols were approved by the NAIST institutional and licensing committees.

### 4.6. Statistical Analysis

Data are presented as the mean ± S.D. and statistical analyses were conducted using SPSS 24. The significance of differences between the two experimental conditions was examined using a Student’s *t*-test. The values for the significance of differences were added to every figure. The values for the significance of differences (*p* values, * *p* < 0.05, ** *p* < 0.01, *** *p* < 0.001, and **** *p* < 0.0001) were added to every figure.

### 4.7. Prediction of Binding Sites of ROS Metabolic Enzymes for Curcumin and Curcumin Derivatives via a Docking Study

The binding sites for curcumin and curcumin derivatives (hs-031, hs-037, hs-047, hs-054, hs-055, hs-056, hs-057, hs-062, hs-064, hs-073, hs-089, and hs-140) on the ROS metabolic enzymes (CBR1, GST-P1, NQO1, PRDX1, AKR1C1, NQO2, and GLO1) were predicted, based on molecular docking simulations. Three-dimensional (3D) structures of keto and enol forms of curcumin and the curcumin derivatives were generated using Balloon software, with options --nconfs 20, --nGenerations 300, and --randomSeed 201,807 from their structural formulas [44]. When more than one conformation was generated, the most stable one was selected as a representative. The high-resolution structures of the ROS metabolic enzymes complexed with their specific co-enzymes or substrates/substrate analogs were retrieved form the Protein Data Bank (PDB) [45] and CBR1 (PDB ID 1wma, chain A, determined to 1.24 Å resolution) [46], GST-P1 (5j41, chains A and B, 1.19 Å) [47], NQO1 (1d4a, chains A and B, 1.7 Å, AKR1C1 (1mrq, chain A, 1.59 Å) [48], NQO2 (4fgl, chains A and B, 1.2 Å) [49], and GLO1 (1qip, chains A and B, 1.72 Å) [50] were selected as the target structures. Since no complex structure with substrate was found, the structure with the highest resolution and least mutations at the active site was selected for PRDX1 (4xcs, chain A and B, 2.10 Å) [51].

The pocket searches on the target structures were then performed using fpocket2 software with default parameters [52]. All pockets detected by fpocket2 (nine pockets for CBR1, 10 pockets for GST-P1, 16 pockets for NQO1, 15 pockets for PRDX1, 11 pockets for AKR1C1, 16 pockets for NQO2, and 17 pockets for GLO1) and the co-enzymes/substrates binding sites in the complex structures were used as the target sites in the docking simulations. The docking simulations were performed using the Schrödinger Release 2018-2 (Schrödinger, LLC, New York, NY, USA, 2019), as follows. The preprocess of the target enzyme structure, which consisted of assigning bond order, adding hydrogen atoms, optimizing H-bond networks, and restraining energy minimization with OPLS3 force field [53], was conducted using the Protein Preparation wizard, after all the HETATM records in the PDB file were removed. Receptor energy grids were generated on the preprocessed structures by setting the OUTERBOX parameter to 30, 30, 30, and GRID_CENTER to the centroid of atoms surrounding a pocket detected by fpocket2, or the centroid of the co-enzyme/substrate. For the curcumin and the curcumin derivatives that were shown to bind to the target enzyme by the pull-down assay, molecular dockings were performed using Glide with the standard precision (SP) mode [54]. Up to 10 poses for each ligand (curcumin or curcumin derivatives) were obtained for each receptor energy grid. Using all the poses obtained from SP mode as initial structures, refinements of the poses with the extra precision (XP) mode were performed [55].

Finally, the propensity for ligand binding was estimated for each amino acid residue of the target enzyme, from the frequencies used for the interface to ligands in the docking poses. The frequencies of residues were counted over the docking poses, with XP scores better than the 75% percentile of XP scores of all poses. The residues were considered as interface residues when at least one atom from the residues was positioned less than 4.0 Å from any of the ligand atoms. Propensity for the ligand binding of residue *i* was evaluated as the relative frequency *f_i_/n*, where *f_i_* was the frequency of residue *i,* and *n* was the number of poses with XP scores better than the 75% percentile.

## Figures and Tables

**Table 1:** Measurement variables of selected curcumin derivatives.

Compound	CLogP ^(1)^	Growth Suppression ^(2)^	Cell Death ^(3)^	1/GI50 ^(4)^	Washout Growth Suppression ^(5)^	Tumor Suppression ^(6)^
Curcumin	2.94	4.83	10.80	0.08	20.00	100.00
hs-031	4.59	7.83	11.80	0.10	20.33	40.32
hs-037	1.91	8.33	15.00	0.31	28.00	95.16
hs-047	4.03	−2.20	2.17	0.07	−99.00	−179.29
hs-054	1.81	3.17	8.00	0.08	15.20	10.16
hs-055	3.20	7.00	8.33	0.05	29.00	69.35
hs-056	3.35	−23.80	1.33	0.03	−98.00	85.48
hs-057	3.32	2.00	4.33	0.11	−21.20	96.77
hs-062	1.88	5.33	1.33	0.06	−24.50	−101.61
hs-064	2.98	4.50	2.33	0.08	23.83	82.26
hs-073	2.47	−20.70	0.50	0.03	−101.00	17.74
hs-089	3.68	2.50	2.83	0.10	2.20	38.71
hs-140	2.65	8.50	4.83	0.06	12.00	−206.45

^(1)^ CLog P values were calculated with ChemDraw 16.0. ^(2)^ Growth suppression: 10 [Cell growth values], Cell growth: 10^5^ cells/mL of K562 cells were cultured in the presence of the compounds, and, after 4 days, the cell concentration was determined and divided by 10^4^, as shown in Figure 2a and Table S1. ^(3)^ Cell death: 10^5^ cells/mL of K562 cells were cultured in the presence of the compounds, and, after 4 days, dead cells were enumerated by the trypan blue exclusion method. The cell concentration was divided by 10^4^, as shown in Figure 2b and Table S1. ^(4)^ 1/GI_50_: the reciprocal of GI_50_ (GI_50_ is shown in Figure 2c and Table S1). ^(5)^ Washout growth suppression: 30 [Washout growth values], Washout growth: 3 × 10^5^ cells/mL of K562 cells were cultured in the presence of the compounds for 2 days and, then, the compounds were removed from the medium. The cells were maintained in compound-free medium for 4 days and enumerated. The cell concentration was divided by 10^4^, as shown in Figure 2f and Table S1. ^(6)^ Tumor suppression was calculated as ([control tumor size] − [sample tumor size])/[control tumor size] × 100.

## Supplementary Materials

The following are available online, Figure S1A: Chemical structure of curcumin and curcumin derivatives used in this study, S1B: Synthetic route of curcumin and curcumin derivatives used in this study, Figure S2A: Growth inhibition by curcumin derivatives, S2B: Growth inhibition of human tumor cells by curcumin derivatives, S2C: Inhibition of tumor formation by curcumin derivatives with few side effects, Figure S3: Spectra of Curcuminoids, Figure S4: Predicted binding sites for curcumin or curcumin derivatives on ROS metabolic enzymes, Spectra of Curcuminoids, Table S1: Summary of measurement variables of selected curcumin derivatives.
