# Supplementary material for: Curcumin Derivatives Verify the Essentiality of ROS Upregulation in Tumor Suppression
Source: Molecules. 2019 Nov 10;24(22):4067. doi: 10.3390/molecules24224067 (PMC6891586; doi:10.3390/molecules24224067)
Supplement: Supplementary file 1 [file molecules-24-04067-s001.pdf]

Legends to Supplementary Figures and Tables

Figure S1
S1A: Chemical structure of curcumin and curcumin derivatives used in this study.
S1B: Synthetic scheme of curcumin and curcumin derivatives used in this study.

Figure S2
S2A: Growth inhibition by curcumin derivatives.
(a, b) The GI_{50} of curcumin derivatives on K562 cells was determined after a 4-day culture as described in Fig. 2c.
S2B: Growth inhibition of human tumor cells by curcumin derivatives.
Each human tumor cell line (HEK293T, AN3CA, U87-MG, MIA PaCa2, HeLa, PANC1, and MCF7, 1-2 x 10^5 cells/mL) was cultured in the presence of each compound (50 μM) for 4 days, and living cells were enumerated using the trypan blue staining method.
S2C: Inhibition of tumor formation by curcumin derivatives with few side effects.
(a-c) After 18 days post-injection, mice were sacrificed, and the white (a) and red (b) cells and platelets (c) in peripheral blood were counted.

Figure S3: Spectra of Curcuminoids

Figure S4: Predicted binding sites for curcumin or curcumin derivatives on ROS metabolic enzymes.
(a) CBR1, (b) GST-P1, (c) NQO1, (d) PRDX1, (e) AKR1C1, (f) NQO2, and (g) GLO1. For each enzyme – ligand combination, the front (co-factor binding site side) and the back (rotated 180 degrees) views are shown on left and right, respectively. The residues are colored by maroon gradation according to the propensity for ligand binding. The bound co-enzymes (NADPH, FAD, and NADP) or substrate/substrate analog (GSH and NBC-GSH) are shown in sphere models colored by blue. The models for curcumin ligand are same as shown in Fig.7.

Table S1: Summary of measurement variables of selected curcumin derivatives.
・CLog P values were calculated with the ChemDraw 16.0.
・Growth (allowed): 10^5 cells/ml of K562 cells were cultured in the presence of the compounds (50 μM), and after 4 days, the cell concentration was determined and divided by 10^4.
・Death (induced): 10^5 cells/ml of K562 cells were cultured in the presence of the compounds (50 μM), and after 4 days, dead cells were enumerated by the trypan blue exclusion method. The cell concentration was divided by 10^4.

・Washout growth (allowed): 3 x 10^5 cells/ml of K562 cells were cultured in the presence of the compounds (50 μM) for 2 days, and, then, the compounds were removed from the medium. Cells were maintained in compound-free medium for 4 days and enumerated. The cell concentration was divided by 10^4.

・b-gal (Day2), b-gal (Day4): K562 cells were cultured in the presence of the compounds (50 μM) for 2 and 4 days, and stained for the SA-β-gal activity. The percentages of SA-β-gal-positive cells were calculated at day2 and day4, respectively.

・ROS (1h, fold), ROS (24h, fold), ROS (72h, fold): K562 cells were cultured in the presence of the compounds (50 μM) for 1, 24, and 72 hours, and stained in the presence of the ROS indicator.

・Tumor formation: K562 cells (2.5 x 10^6 cells) were transplanted s.c. into the flanks of nude mice. Mice were then treated with curcumin derivatives (25 mg/kg BW) in corn oil and vehicle (corn oil) via an i.p. injection every 2 days. After 18 days post-injection, mice were sacrificed, tumors were taken, and tumor weights were measured. Tumor formation was calculated as the percentage of the sample tumor size compared to the control tumor size.

Figure S1A - Related to Figure 1

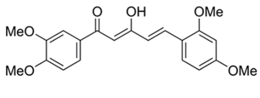

hs-129

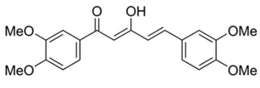

hs-141

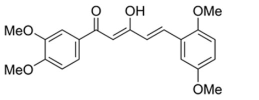

hs-142

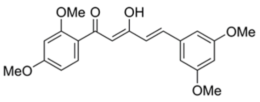

hs-143

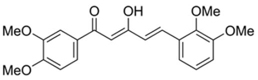

hs-144

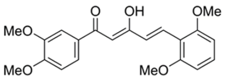

hs-145

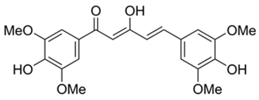

hs-149

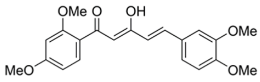

hs-150

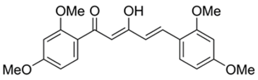

hs-151

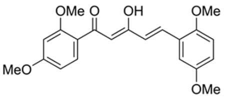

hs-152

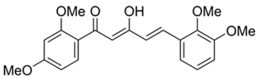

hs-153

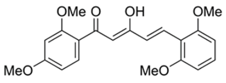

hs-154

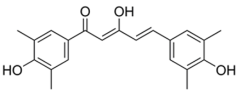

hs-157

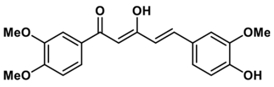

hs-127
CLogP: 2.85
Chemical Formula: $C_{20}H_{20}O_6$
Molecular Weight: 356.37

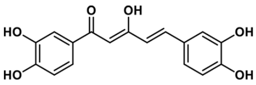

hs-131
CLogP: 1.95
Chemical Formula: $C_{17}H_{14}O_6$
Molecular Weight: 314.29

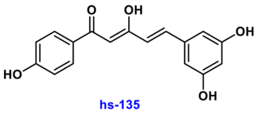

hs-135
CLogP: 2.31
Chemical Formula: $C_{17}H_{14}O_5$
Molecular Weight: 298.29

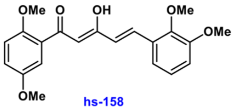

hs-158
CLogP: 3.68
Chemical Formula: $C_{21}H_{22}O_6$
Molecular Weight: 370.4010

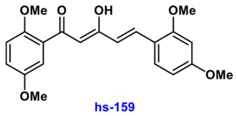

hs-159
CLogP: 4.03
Chemical Formula: $C_{21}H_{22}O_6$
Molecular Weight: 370.4010

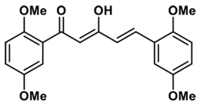

hs-160
CLogP: 4.03
Chemical Formula: $C_{21}H_{22}O_6$
Molecular Weight: 370.4010

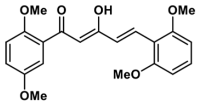

hs-161
CLogP: 4.03
Chemical Formula: $C_{21}H_{22}O_6$
Molecular Weight: 370.4010

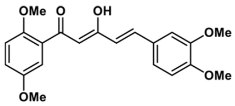

hs-162
CLogP: 3.68
Chemical Formula: $C_{21}H_{22}O_6$
Molecular Weight: 370.4010

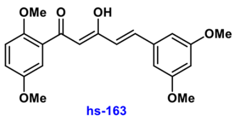

hs-163
CLogP: 4.03
Chemical Formula: $C_{21}H_{22}O_6$
Molecular Weight: 370.4010

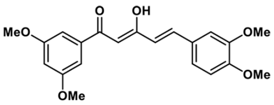

HF-010
CLogP: 3.68
Chemical Formula: $C_{21}H_{22}O_6$
Molecular Weight: 370.4010

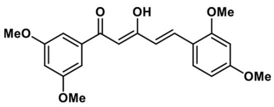

HF-014
CLogP: 4.03
Chemical Formula: $C_{21}H_{22}O_6$
Molecular Weight: 370.4010

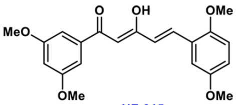

HF-015
CLogP: 4.03
Chemical Formula: $C_{21}H_{22}O_6$
Molecular Weight: 370.4010

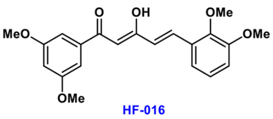

HF-016
CLogP: 3.68
Chemical Formula: $C_{21}H_{22}O_6$
Molecular Weight: 370.4010

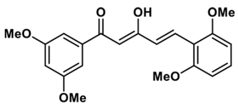

HF-017
CLogP: 4.03
Chemical Formula: $C_{21}H_{22}O_6$
Molecular Weight: 370.4010

Supplementary Fig. S1A. Chemical structure of curcumin and curcumin derivatives used in this study.

Figure S1B – Related to Figure 1

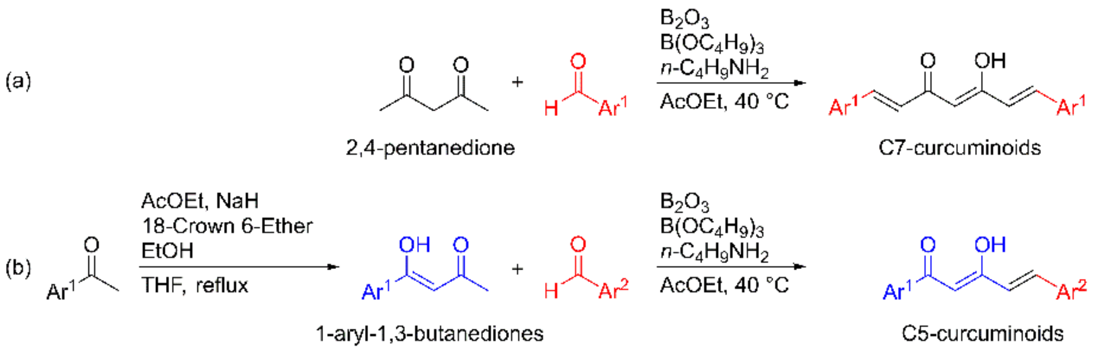

Supplementary Fig. S1B. Synthetic scheme of curcumin and curcumin derivatives used in this study.

General considerations.

NMR spectra were recorded on a 500 MHz spectrometer. Chemical shifts of ^{1}H NMR spectra are given in ppm using the solvent signal as the internal standard ($CDCl_3$, 7.26 ppm). Data are reported as follows: multiplicity (s = singlet, d = doublet, t = triplet, q = quartet, and m = multiplet), coupling constant in Hz, and integration. ^{13}C NMR chemical shifts are given in ppm using the solvent signal ($CDCl_3$, 77.0 ppm) as the internal standard. Infrared spectra (IR) were collected on a JASCO FT/IR-4200 spectrometer; absorption peaks are reported in reciprocal centimeters (cm^{-1}) with the following relative intensities: s (strong), m (medium), or w (weak). Mass spectra were obtained with ionization voltages of 70 eV. Column chromatography was performed using a SiO_2 (MERCK Silica gel 60).

Materials.

4-Hydroxybenzaldehyde, 2,4-dimethoxybenzaldehyde, 2,5-dimethoxybenzaldehyde, 3,4-dimethoxybenzaldehyde, 3,5-dimethoxybenzaldehyde, 3,4,5-trimethoxybenzladehyde, 2’4’-dimethoxyacetophenone, 2’5’-dimethoxyacetophenone, and 3’4’-dimethoxyacetophenone were purchased from Tokyo Chemical Industry Co., Ltd. 4-Hydroxy-3,5-dimethylbenzaldehyde, 3’5’-dimethoxyacetophenone, 3’,4’,5’-trimethoxyacetophenone were purchased from Sigma-Aldrich Co., Llc.

Demethylation of C5-curcuminoids were performed using the modified method reported previously (Scheme S1) [1]. Selective demethylation of *para*-methoxy group on the aromatic ring was performed using the modified method reported previously (Scheme S2) [2].

[1] Shirali, A.; Sriram, M.; Hall, J. J.; Nguyen, B. L.; Guddneppanavar, R.; Mallinath B. Hadimani, M. B.; Ackley, J. F.; Siles, R.; Jelinek, C. J.; Arthasery, P.; Brown, R. C.; Murrell, V. L.; McMordie, A.; Sharma, S.; Chaplin, D. J.; Pinney, K. G., *J Nat Prod* **2009**, 72, (3), 414-421. Development of Synthetic Methodology Suitable for the Radiosynthesis of Combretastatin A-1 (CA1) and Its Corresponding Prodrug CA1P.
[2] McOmie, J. F. W.; West, D. E., *Org Synth* **1969**, 49, 50-52. 3,3'-Dihydroxybiphenyl.

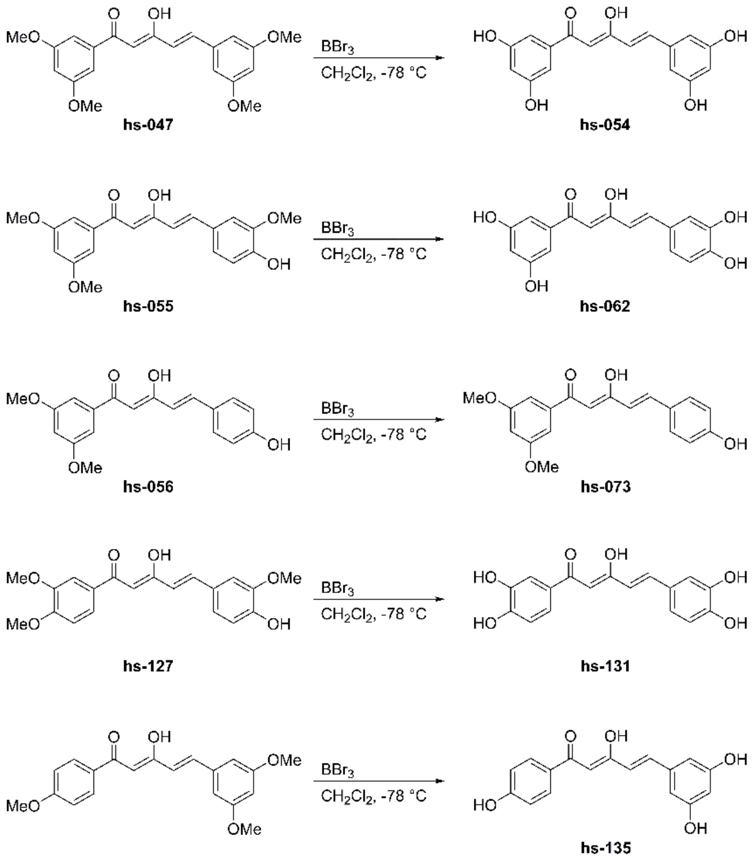

Scheme S1. Demethylation of MeO-groups on aromatic ring.

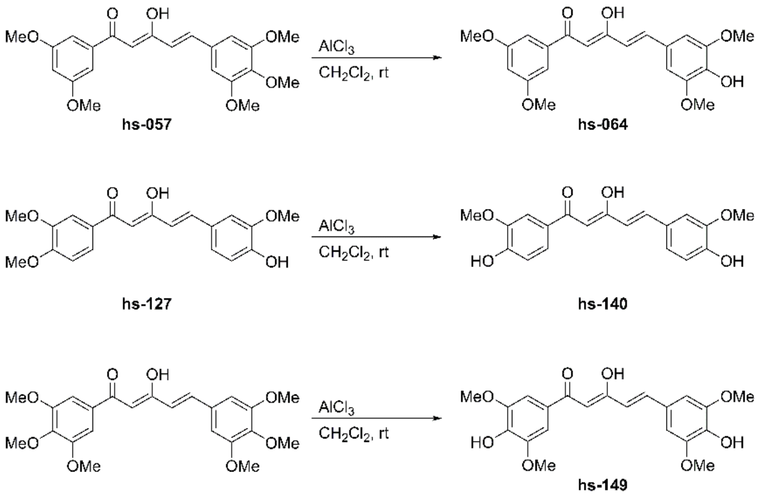

Scheme S2. Selective demethylation of *para*-MeO-group.

C7-Curcuminoids

hs-031 (1*E*,4*Z*,6*E*)-1,7-bis(3,5-dimethoxyphenyl)-5-hydroxyhepta-1,4,6-trien-3-one. Yellow powder; mp 145.7-146.9 °C; *Rf* 0.57 (hexane/EtOAc = 1/1); ^{1}H NMR (CD_3COCD_3, 500 MHz) δ 3.83 (s, 12H), 6.11 (s, 1H), 6.55 (m, 2H), 6.87-6.91 (m, 6H), 7.60 (d, *J*=15.5 Hz, 2H); ^{13}C NMR (CD_3COCD_3, 500 MHz) δ 55.9, 102.7, 103.3, 107.0, 125.7, 138.0, 141.4, 162.3, 184.3; IR (KBr) 3001, 2937, 2838, 1593, 1457, 1427, 1357, 1302, 1276, 1205, 1156, 1065, 978, 924, 829, 675; MS m/z (relative intensity, %) 396 (100), 378 (32), 325(17), 295 (3), 259 (6), 245 (17), 205 (72), 191 (86), 148 (29), 133 (8), 119 (7), 91 (10), 77 (8), 69 (40); exact mass-EI calcd for $C_{23}H_{24}O_6$ 396.1580, found 396.1573.

hs-037 (1*E*,4*Z*,6*E*)-1,7-bis(3,5-dihydroxyphenyl)-5-hydroxyhepta-1,4,6-trien-3-one. Brown powder; mp 241.2-242.4 °C; *Rf* 0.34 ($CHCl_3$/MeOH = 4/1); ^{1}H NMR (CD_3COCD_3, 500 MHz) δ 6.09 (s, 1H), 6.44 (m, 2H), 6.66 (m, 2H), 6.72 (d, *J*=16.0 Hz, 2H), 7.51 (d, *J*=16.0, 2H), ^{13}C NMR (CD_3COCD_3, 500 MHz) δ 102.4, 105.6, 107.5, 124.8, 137.8, 141.5, 159.7, 184.3; IR (KBr) 3261, 1631, 1597, 1445, 1381, 1338, 1301, 1201, 1147, 1012, 996, 966, 835, 722, 671, 514; MS m/z (relative intensity, %) 340 (21), 324 (8), 282 (15), 254 (6), 217 (5), 178 (50), 163 (100), 149 (27), 107 (26), 77 (22), 69 (14); exact mass-EI calcd for $C_{19}H_{16}O_6$ 340.0946, found 340.0946.

1-Aryl-1,3-butanediones

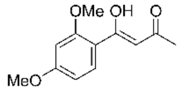

1-(2,4-Dimethoxyphenyl)butane-1,3-dione. Yield 66%; Pale yellow crystal; mp 61.5–63.2 °C (hexane/toluene); R_f 0.36 (hexane/AcOEt = 2/1); ^{1}H NMR (500 MHz, $CDCl_3$); ^{1}H NMR (500 MHz, $CDCl_3$) 16.49 (brs, 1H), 7.93 (d, J = 9.0 Hz, 1H), 6.57 (dd, J = 8.5, 1.5 Hz, 1H), 6.50–6.45 (m, 2H), 3.90–3.86 (m, 6H), 2.17 (s, 3H); ^{13}C NMR (126 MHz, $CDCl_3$) δ 193.8, 181.2, 164.0, 160.3, 132.1, 116.9, 105.2, 100.9, 98.7, 55.7, 55.6, 26.1; IR (KBr) 2981 w, 2952 w, 1606 s, 1507 s, 1456 m, 1434 m, 1328 m, 1256 s, 1212 s, 1124 s, 1077 m, 1021 s, 943 m, 832 m, 790 m, 669 w; LRMS (EI, M = $C_{12}H_{14}O_4$) *m/z* 222 (26, M^+), 191 (23), 166 (10), 165 (100); HRMS (EI) calcd for $C_{12}H_{14}O_4$ (M^+) 222.0892, found 222.0896.

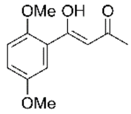

1-(2,5-Dimethoxyphenyl)butane-1,3-dione. Yield 61%; Pale yellow oil; R_f 0.44 (hexane/AcOEt = 2/1); ^{1}H NMR (500 MHz, $CDCl_3$) δ 16.25 (brs, 1H), 7.43 (d, J = 3.0 Hz, 1H), 7.00 (dd, J = 9.5, 2.0 Hz 1H), 6.91 (d, J = 9.5 Hz, 1H), 6.48 (s, 1H), 3.88 (s, 3H), 3.81 (s, 3H), 2.19 (s, 3H); ^{13}C NMR (126 MHz, $CDCl_3$) δ 194.6, 181.1, 153.5, 152.9, 124.5, 119.2, 114.1, 113.1, 102.0, 56.3, 55.9, 26.2; IR (NaCl, neat) 3001 w, 2946 w, 2835 w, 1718 w, 1576 s, 1498 s, 1465 m, 1414 m, 1362 w, 1281 m, 1223 s, 1171 m, 1045 m, 1021 m, 812 m, 729 m; LRMS (EI, M = $C_{12}H_{14}O_4$) *m/z* 222 (39, M^+), 192 (12), 191 (100), 165 (70); HRMS (EI) calcd for $C_{12}H_{14}O_4$ (M^+) 222.0892, found 222.0895.

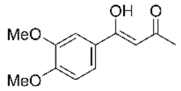

1-(3,4-Dimethoxyphenyl)butane-1,3-dione. Yield 46%; Pale yellow crystal; mp 73.5–74.6 °C (hexane/toluene); R_f 0.34 (hexane/AcOEt = 2/1); ^{1}H NMR (500 MHz, $CDCl_3$) δ 16.32 (brs, 1H), 7.52–7.47 (m, 2H), 6.91 (d, J = 8.5 Hz, 1H), 6.14 (s, 1H), 3.96–3.95 (m, 6H), 2.19 (s, 3H); ^{13}C NMR (126 MHz, $CDCl_3$) δ 190.7 184.9, 152.8, 149.0, 128.0, 121.2, 110.4, 109.5, 96.0, 56.1, 56.0, 25.1; IR (KBr) 3437 w, 2964 w, 2841 w, 1598 s, 1515 s, 1441 s, 1333 w, 1265 s, 1233 s, 1167 s, 1146 s, 1017 m, 984 w, 893 w, 775 m, 668 w; LRMS (EI, M = $C_{12}H_{14}O_4$) *m/z* 223 (14), 222 (100, M^+), 221 (21), 207 (30), 191 (15), 165 (55), 139 (23), 138 (10), 124 (30), 69 (13); HRMS (EI) calcd for $C_{12}H_{14}O_4$ (M^+) 222.0892,

found 222.0889.

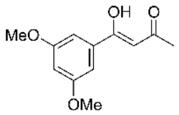

1-(3,5-Dimethoxyphenyl)butane-1,3-dione. Pale yellow crystal; mp 56.7–58.0 °C (hexane/toluene); R_f 0.31 (hexane/AcOEt = 4/1); ^{1}H NMR (500 MHz, $CDCl_3$) δ 16.12 (brs, 1H), 7.01 (d, J = 1.5 Hz, 2H), 6.61 (t, J = 2.5 Hz, 1H), 6.13 (s, 1H), 3.84 (s, 6H), 2.20 (s, 3H); ^{13}C NMR (126 MHz, $CDCl_3$) δ 193.3, 183.8, 161.0, 137.1, 104.9, 104.7, 97.0, 55.7, 25.8; IR (KBr) 3429 w, 3093 m, 2942 m, 2839 w, 1596 s, 1455 s, 1349 s, 1300 s, 1207 s, 1159 s, 1045 s, 972 m, 923 m, 864 s, 795 s, 677 s; LRMS (EI, M = $C_{12}H_{14}O_4$) *m/z* 223 (14), 222 (100, M^+), 221 (25), 207 (34), 191 (17), 165 (27), 139 (22), 69 (12); HRMS (EI) calcd for $C_{12}H_{14}O_4$ (M^+) 222.0892, found 222.0891.

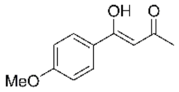

1-(4-Methoxyphenyl)butane-1,3-dione. White crystal; mp 53.2–53.9 °C (hexane); R_f 0.37 (hexane/AcOEt = 3/1); ^{1}H NMR (500 MHz, $CDCl_3$) δ 16.34 (brs, 1H), 7.88–7.86 (m, 2H), 6.96–6.94 (m, 2H), 6.13 (s, 1H), 3.87 (s, 3H), 2.18 (s, 3H); ^{13}C NMR (126 MHz, $CDCl_3$) δ 191.7, 184.2, 163.1, 129.2, 127.5, 114.0, 95.9, 55.5, 25.4; IR (KBr) 3728 w, 3424 w, 2966 w, 2841 w, 1606 s, 1505 m, 1465 m, 1288 w 1260 s, 1181 m, 1117 w, 1026 m, 842 m, 781 m, 681 w, 669 w; LRMS (EI, M = $C_{11}H_{12}O_3$) *m/z* 193 (13), 192 (100, M^+), 191 (30), 177 (74), 161 (10), 135 (84), 109 (21), 108 (12), 92 (11), 77 (16), 69 (24); HRMS (EI) calcd for $C_{11}H_{12}O_3$ (M^+) 192.0786, found 192.0785.

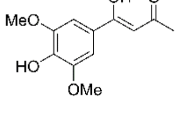

1-(4-Hydroxy-3,5-dimethoxyphenyl)butane-1,3-dione. Yield 77%; Brown crystal; mp 142.6–143.5 °C (hexane/dichloromethane); R_f 0.34 (hexane/AcOEt = 1/1); ^{1}H NMR (500 MHz, $CDCl_3$) δ 16.32 (brs, 1H), 7.18 (s, 2H), 6.11 (s, 1H), 5.93 (brs, 1H), 3.97 (s, 6H), 2.19 (s, 3H); ^{13}C NMR (126 MHz, $CDCl_3$) δ 190.8, 185.0, 147.0, 139.1, 126.5, 104.4, 96.0, 56.5, 25.1; IR (KBr) 3303 m, 2947 w, 2843 w, 1610 s, 1542 m, 1517 m, 1473 m,

1422 m, 1327 s, 1235 s, 1170 m, 1110 s, 1032 w, 908 w, 776 m, 667 w; LRMS (EI, M = $C_{12}H_{14}O_5$) *m/z* 239 (13), 238 (100, M^+), 237 (14), 223 (16), 207 (11), 182 (10), 181 (99), 155 (23), 154 (12), 153 (10), 149 (17), 140 (25), 69 (10), 58 (72); HRMS (EI) calcd for $C_{12}H_{14}O_5$ (M^+) 238.0841, found 238.0835.

C5-Curcuminoids

C5-curcuminoids were synthesized from 1-aryl-1,3-diketones which are prepared as the above, with commercially available aldehydes. See the text.

Figure S2A – Related to Figure 2

(**a**)

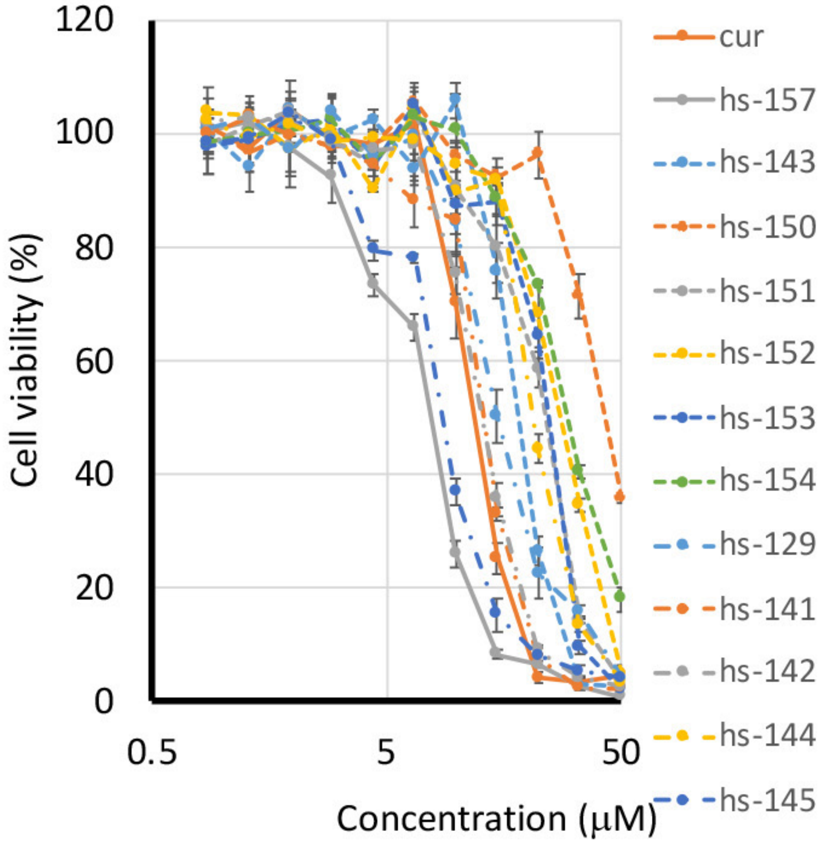

	GI50(μM)
curcumin	11.9
hs-157	7.74
hs-150	42.5
hs-151	24.0
hs-143	18.3
hs-152	27.7
hs-153	24.7
hs-154	29.6
hs-129	14.8
hs-141	13.0
hs-142	12.9
hs-144	21.2
hs-145	8.68

(**b**)

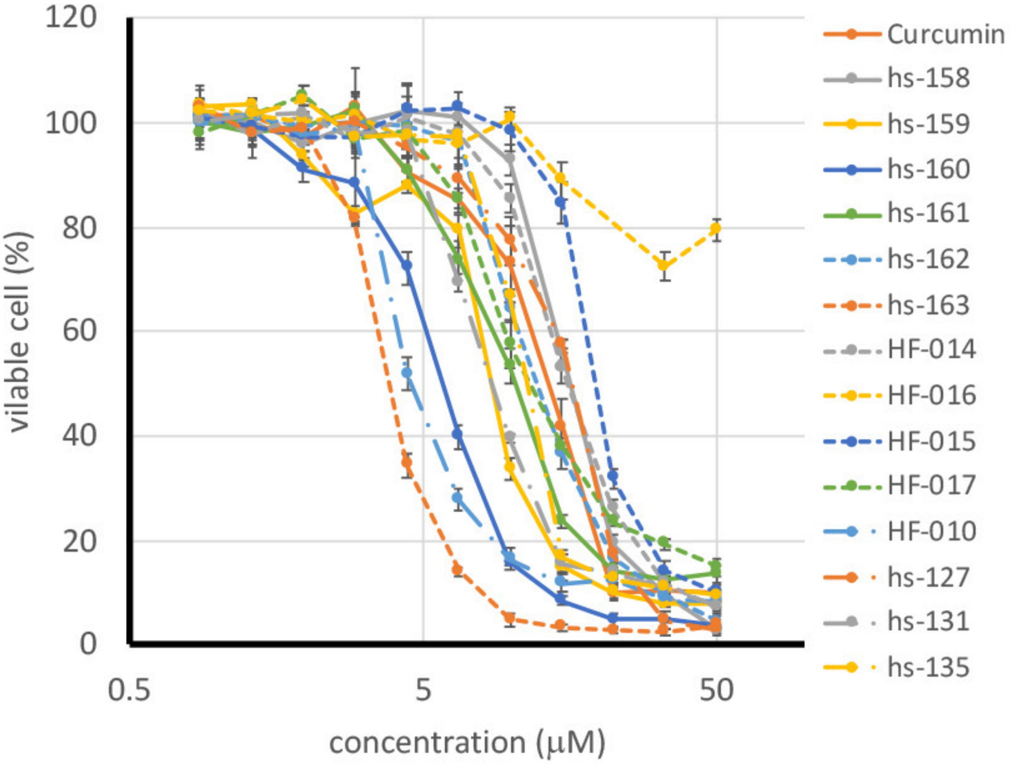

	GI 50 (μM)
cur	11.86
hs-158	15.81
hs-159	8.54
hs-160	5.80
hs-161	10.34
hs-162	12.19
hs-163	3.84
HF-014	15.50
HF-016	101.12
HF-015	19.33
HF-017	11.56
HF-010	4.53
hs-127	16.01
hs-131	8.56
hs-135	11.32

Supplementary Fig. S2A. Growth inhibition by curcumin derivatives.
(a, b) The GI50 of curcumin derivatives on K562 cells

Figure S2B - Related to Figure 2

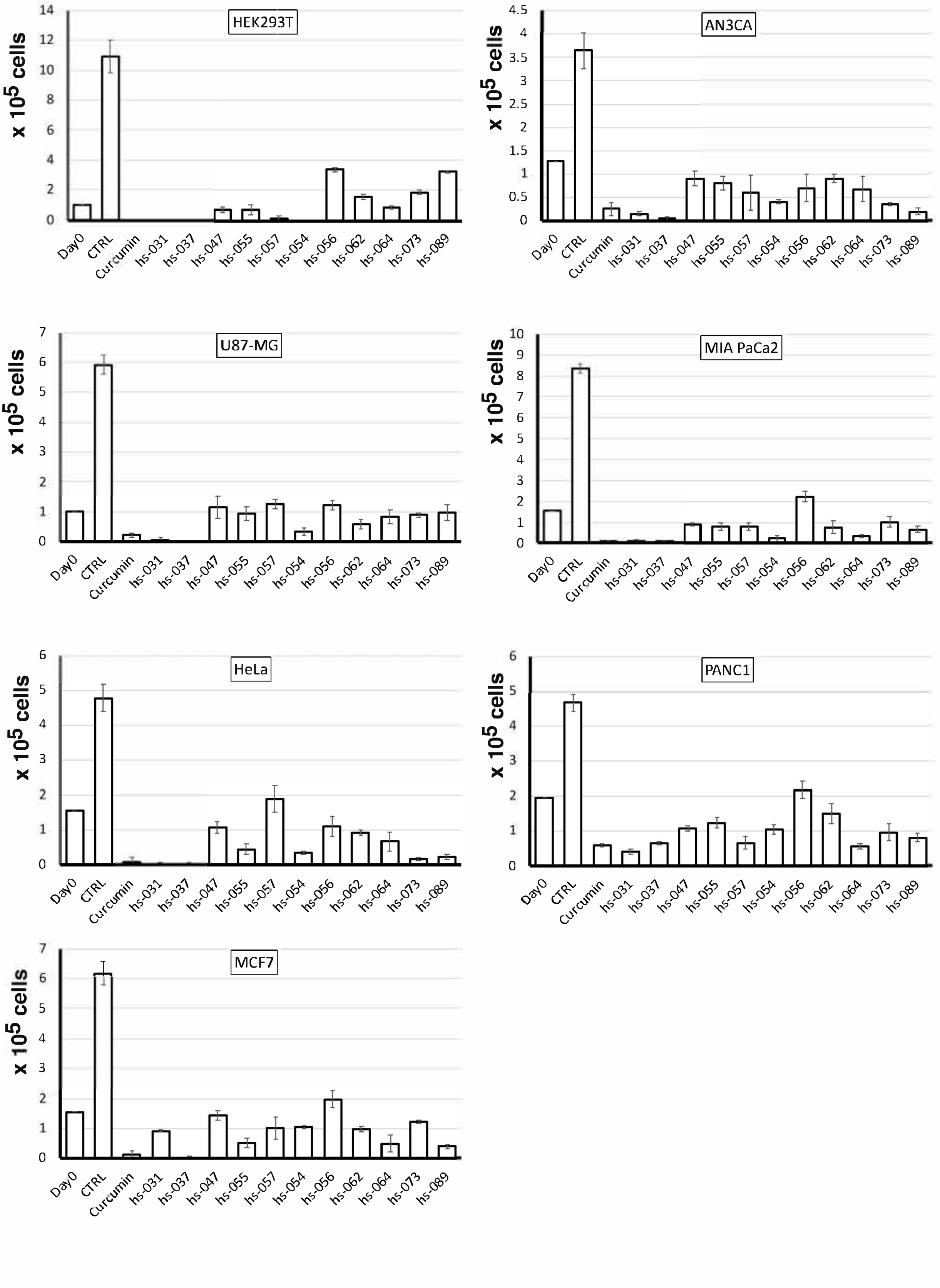

Supplementary Fig. S2B. Growth inhibition of human tumor cells by curcumin derivatives

Figure S2C – Related to Figure 4

(a)

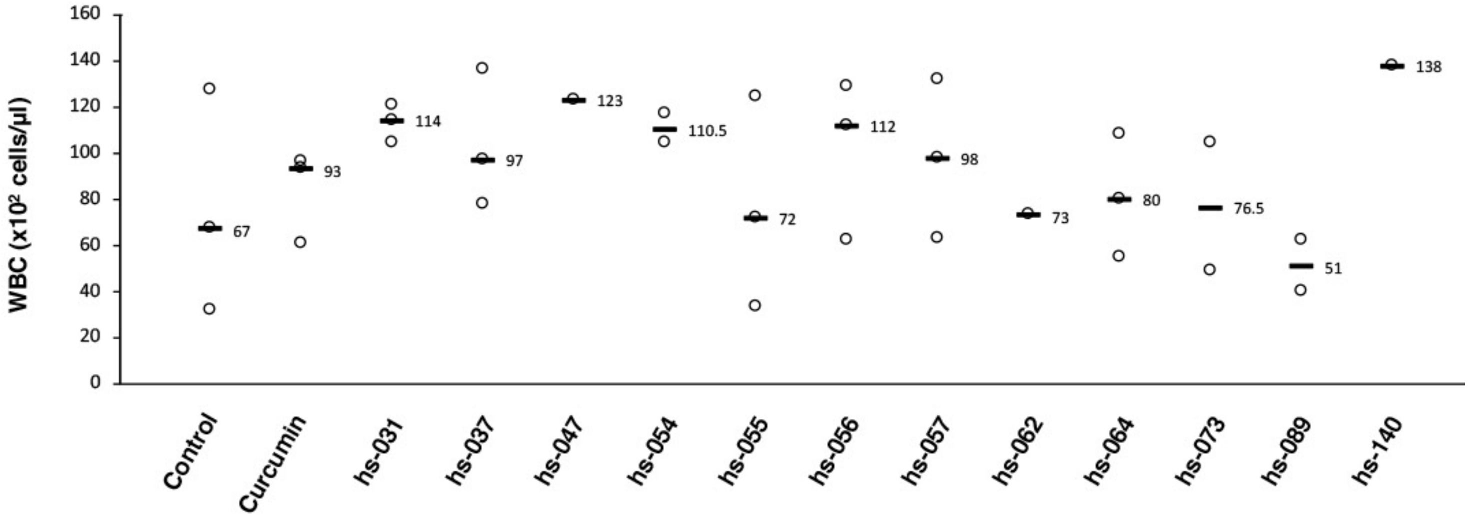

(b)

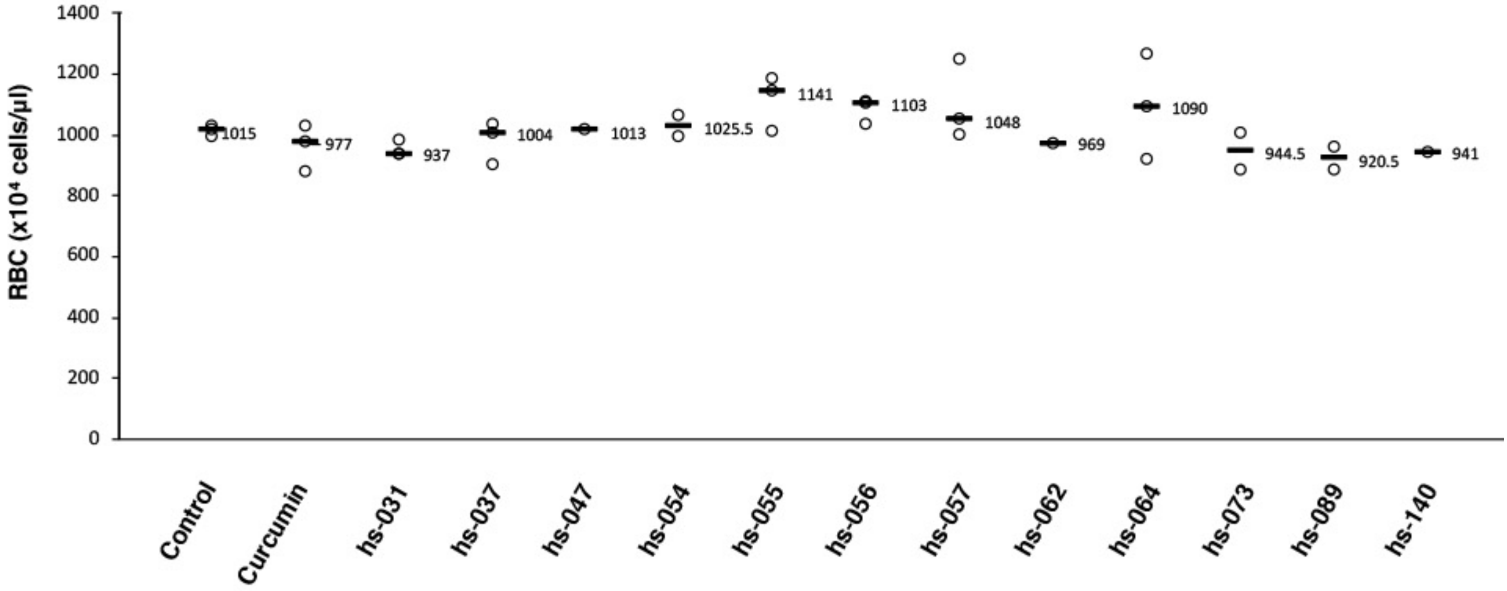

(c)

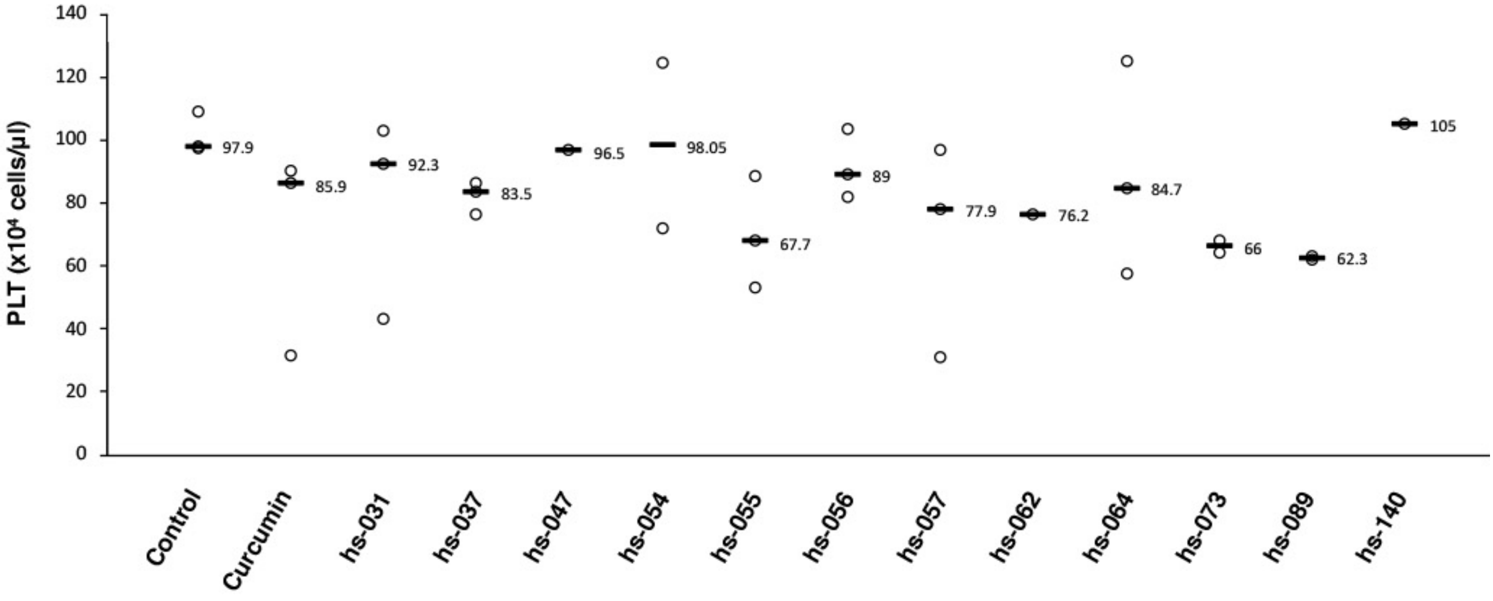

Supplementary Fig. S2C. Inhibition of tumor formation by curcumin derivatives with few side effects. (a) After 18 days post-injection, mice were sacrificed, and tumor sizes were measured. (b–d) When sacrificed, the white (b) and red (c) cells and platelets (d) in peripheral blood were counted.

Figure S3 - Related to Figure 1

Spectra of Curcuminoids

hs-047 ((*E*)-1,5-Bis(3,5-dimethoxyphenyl)pent-4-ene-1,3-dione). Yield 48%; Yellow needle; mp 105.4–106.7 °C (MeOH); R_f 0.57 (hexane/AcOEt = 1/1); ^{1}H NMR (500 MHz, CD_3COCD_3) δ 16.28 (brs, 1H), 7.64 (d, *J* = 15.5 Hz, 1H), 7.17 (d, *J* = 2.0 Hz, 2H), 6.93 (d, *J* = 16.0 Hz, 1H), 6.88 (d, *J* = 1.5 Hz, 2H), 6.74 (t, *J* = 2.0 Hz, 1H), 6.67 (s, 1H), 6.55 (t, *J* = 2.0 Hz, 1H), 3.87 (s, 3H), 3.84 (s, 3H); ^{13}C NMR (126 MHz, CD_3COCD_3) δ 190.5, 180.2, 162.3, 162.2, 140.8, 139.3, 138.1, 124.9, 106.8, 106.1, 105.6, 103.2, 98.8, 56.1, 55.9; IR (KBr) 3445 w, 2993 w, 2940 w, 2836 w, 1637 m, 1595 s, 1457 m, 1424 m, 1326 m, 1207 m, 1153 s, 1065 m, 964 w, 820 w, 776 w, 698 w; LRMS (EI, M = $C_{21}H_{22}O_6$) *m/z* 371 (23), 370 (88, M$^+$), 352 (18), 219 (16), 215 (14), 206 (15), 205 (100), 204 (31), 192 (11), 191 (86), 190 (17), 185 (11), 174 (12), 166 (35), 165 (40), 148 (16), 138 (49), 137 (13), 122 (11), 81 (13), 71 (12), 69 (26), 57 (17); HRMS (EI) calcd for $C_{21}H_{22}O_6$ (M$^+$) 370.1416, found 370.1421.

hs-054 ((*E*)-1,5-Bis(3,5-dihydroxyphenyl)pent-4-ene-1,3-dione). Yield 40% (from **2aa**); Brown powder; mp 225.0–226.4 °C; R_f 0.31 ($CHCl_3$/MeOH = 5/1); ^{1}H NMR (500 MHz, CD_3COCD_3) δ 8.67–8.53 (m, 4H), 7.54 (d, *J* = 16.0 Hz, 1H), 7.01 (d, *J* = 2.0 Hz, 2H), 6.79 (d, *J* = 15.5 Hz, 1H), 6.67 (d, *J* = 2.0 Hz, 2H), 6.60 (t, *J* = 2.0 Hz, 1H), 6.54 (s, 1H), 6.44 (t, *J* = 2.0 Hz, 1H); ^{13}C NMR (126 MHz, CD_3COCD_3) δ 190.5, 180.4, 159.94, 159.86, 140.9, 139.3, 138.1, 124.4, 107.9, 107.5, 106.8, 105.6, 98.6; IR (KBr) 3322 s, 1638 s, 1599 s, 1548 s, 1509 m, 1473 m, 1342 s, 1308 s, 1211 m 1159 s, 998 m, 968 w, 832 w, 786 w, 695 w, 658 w; LRMS (EI, M = $C_{17}H_{14}O_6$) *m/z* 314 (39, M$^+$), 296 (14), 187 (10), 178 (10), 177 (46), 176 (20), 163 (43), 152 (50), 138 (27), 137 (100), 134 (12), 129 (10), 110 (20), 109 (42), 107 (12), 81 (19), 77 (13), 73 (17), 69 (34), 60 (12), 57 (13), 55 (14); HRMS (EI) calcd for $C_{17}H_{14}O_6$ (M$^+$) 314.0790, found 314.0790.

hs-055 ((*E*)-1-(3,5-dimethoxyphenyl)-5-(4-hydroxy-3-methoxyphenyl)pent-4-ene-1,3-dione). Yield 62%; Yellow powder; mp 136.2–137.3 °C (MeOH); R_f 0.49 (hexane/AcOEt = 1/1); ^{1}H NMR (500 MHz, CD_3COCD_3) δ 8.26 (brs, 1H), 7.67 (d, *J* = 15.5 Hz, 1H), 7.35 (d, *J* = 1.5 Hz, 1H), 7.20 (dd, *J* =

8.0, 2.0 Hz, 1H), 7.15 (d, *J* = 2.0 Hz, 2H), 6.90 (d, *J* = 8.0 Hz, 1H), 6.77 (d, *J* = 16.0 Hz, 1H), 6.71 (t, *J* = 2.0 Hz, 1H), 6.60 (s, 1H), 3.93 (s, 3H), 3.86 (s, 6H); ^{13}C NMR (126 MHz, CD_3COCD_3) δ 189.0, 181.7, 162.0, 150.0, 148.7, 141.4, 139.1, 128.1, 123.9, 121.3, 116.2, 111.3, 105.7, 105.2, 97.9, 56.2, 55.8; IR (KBr) 3418 w, 2944 w, 2840 w, 1631 m, 1602 s, 1512 s, 1457 m, 1429 m, 1341 m, 1279 m, 1204 s, 1158 s, 1065 w, 1030 w, 972 w, 853 w; LRMS (EI, M = $C_{20}H_{20}O_6$) *m/z* 357 (15), 356 (64, M^+), 338 (11), 221 (10), 220 (11), 201 (11), 191 (50), 190 (12), 178 (16), 177 (100), 167 (10), 166 (93), 165 (58), 150 (16), 145 (24), 139 (13), 138 (82), 137 (24), 131 (12), 122 (16), 117 (10), 77 (11), 69 (10); HRMS (EI) calcd for $C_{20}H_{20}O_6$ (M^+) 356.1260, found 356.1260.

hs-056 ((*E*)-1-(3,5-Dimethoxyphenyl)-5-(4-hydroxyphenyl)pent-4-ene-1,3-dione). Yield 50%; Yellow powder; mp 135.8–136.4 °C (MeOH); R_f 0.50 (hexane/AcOEt = 1/1); ^{1}H NMR (500 MHz, CD_3COCD_3) δ 8.99 (brs, 1H), 7.68 (d, *J* = 16.0 Hz, 1H), 7.59 (d, *J* = 8.5 Hz, 2H), 7.16 (d, *J* = 2.5 Hz, 2H), 6.92 (d, *J* = 9.0 Hz, 2H), 6.76–6.71 (m, 2H), 6.63 (s, 1H), 3.87 (s, 6H); ^{13}C NMR (126 MHz, CD_3COCD_3) δ 189.1, 181.8, 162.0, 160.7, 141.2, 139.2, 131.1, 127.6, 121.1, 116.9, 105.9, 105.3, 98.1, 55.9; IR (KBr) 3545 w, 3183 w, 1630 m, 1606 m, 1587 s, 1456 m, 1349 w, 1286 m, 1207 m, 1163 s, 1068 w, 1049 w, 973 w, 923 w, 822 m, 783 w; LRMS (EI, M = $C_{20}H_{20}O_5$) *m/z* 327 (15), 326 (73, M^+), 325 (10), 284 (11), 256 (14), 166 (71), 165 (65), 161 (34), 160 (12), 148 (11), 147 (100), 139 (14), 138 (75), 137 (15), 129 (12), 122 (15), 120 (19), 119 (20), 107 (12), 91 (17), 85 (15), 83 (10), 77 (11), 73 (20), 71 (20), 69 (16), 65 (10), 60 (15), 59 (12), 57 (28), 55 (17); HRMS (EI) calcd for $C_{19}H_{18}O_5$ (M^+) 326.1154, found 326.1157.

hs-057 ((*E*)-1-(3,5-Dimethoxyphenyl)-5-(3,4,5-trimethoxyphenyl)pent-4-ene-1,3-dione). Yield 57%; pale yellow powder; mp 112.8–113.6 °C (MeOH); R_f 0.57 (hexane/AcOEt = 1/1); ^{1}H NMR (500 MHz, CD_3COCD_3) δ 7.66 (d, *J* = 16.0 Hz, 1H), 7.16 (d, *J* = 1.5 Hz, 2H), 7.05 (s, 2H), 6.88 (d, *J* = 16.0 Hz, 1H), 6.73 (t, *J* = 2.0 Hz, 1H), 6.63 (s, 1H), 3.90 (s, 6H), 3.87 (s, 6H), 3.77 (s, 3H); ^{13}C NMR (126 MHz, CD_3COCD_3) δ 189.8, 180.6, 161.9, 154.5, 141.0, 140.9, 139.0, 131.5, 123.3, 106.3, 105.8, 105.3, 98.3, 60.6, 56.3, 55.8; IR (KBr) 3439 w, 2940 w, 2838 w, 1637 m, 1587 s, 1505 m, 1452 m, 1419 m, 1345 m, 1301 m, 1208 m, 1155 s, 1124 s, 1063 m, 1005 w, 821 w; IR (KBr) 3439 m, 2940 w, 2838 w, 1637 s, 1587 s, 1505 m, 1452 m, 1419 m, 1345 s, 1301 m, 1208 m, 1155 s, 1124 s, 1063 m, 1005

w, 821 w; LRMS (EI, M = $C_{22}H_{24}O_7$) *m/z* 401 (23), 400 (94, M^+), 382 (25), 235 (32), 234 (10), 221 (100), 220 (18), 219 (11), 205 (13), 204 (46), 190 (10), 181 (17), 167 (10), 166 (93), 165 (50), 139 (10), 138 (76), 137 (17), 122 (15); HRMS (EI) calcd for $C_{22}H_{24}O_7$ (M^+) 400.1522, found 400.1525.

hs-062 ((*E*)-5-(3,4-Dihydroxyphenyl)-1-(3,5-dihydroxyphenyl)pent-4-ene-1,3-dione). Yield 38%; Brown powder; mp 217.5–218.4 °C; R_f 0.14 ($CHCl_3$/MeOH = 5/1); ^{1}H NMR (500 MHz, CD_3COCD_3) δ 8.64 (m, 4H), 7.59 (d, *J* = 16.0 Hz, 1H), 7.20 (d, *J* = 2.5 Hz, 1H), 7.09 (dd, *J* = 8.5, 2.0 Hz, 1H) 7.00 (d, *J* = 2.5 Hz, 2H), 6.89 (d, *J* = 8.5 Hz, 1H), 6.69 (d, *J* = 15.5 Hz, 1H), 6.58 (t, *J* = 2.0 Hz, 1H), 6.48 (s, 1H); ^{13}C NMR (126 MHz, CD_3COCD_3) δ 189.2, 181.7, 159.6, 148.7, 146.4, 141.2, 139.2, 128.2, 122.6, 121.2, 116.4, 115.1, 107.6, 106.5, 97.8; IR (KBr) 3315 m, 1635 s, 1605 s, 1578 s, 1542 s, 1463 m, 1387 m, 1342 s, 1283 s, 1158 s, 1118 s, 1004 m, 964 m, 848 m, 805 m, 758 w; LRMS (EI, M = $C_{17}H_{14}O_6$) *m/z* 314 (23, M^+), 296 (11), 178 (20), 177 (27), 176 (12), 163 (51), 159 (11), 152 (49), 138 (42), 137 (100), 136 (19), 135 (17), 134 (11), 131 (12), 110 (21), 109 (39), 89 (15), 81 (18), 77 (12), 69 (43); HRMS (EI) calcd for $C_{17}H_{14}O_6$ (M^+) 314.0790, found 314.0787.

hs-064 ((*E*)-1-(3,5-Dimethoxyphenyl)-1-(4-hydroxy-3,5-dimethoxyphenyl)pent-4-ene-1,3-dione). Yield 70%; Pale orange powder; mp 151.0–151.9 °C (MeOH); R_f 0.37 (hexane/AcOEt = 1/1); ^{1}H NMR (500 MHz, CD_3COCD_3) δ 7.91 (brs, 1H), 7.66 (d, *J* = 15.5 Hz, 1H), 7.15 (d, *J* = 2.0 Hz, 2H), 7.06 (s, 2H), 6.80 (s, *J* = 16.0 Hz, 1H), 6.72 (t, *J* = 2.0 Hz, 1H), 6.59 (s, 1H), 3.90 (s, 6H), 3.87 (s, 6H); ^{13}C NMR (126 MHz, CD_3COCD_3) δ 189.1, 181.6, 162.0, 149.0, 141.8, 139.5, 139.2, 126.8, 121.5, 106.7, 105.8, 105.2, 97.9, 56.6, 55.9; IR (KBr) 3404 m, 2939 w, 2835 w, 1632 m, 1586 s, 1513 s, 1462 s, 1345 s, 1317 m, 1205 m, 1156 s, 1119 s, 1055 m, 975 w, 821 w, 668 w; LRMS (EI, M = $C_{21}H_{22}O_7$) *m/z* 387 (23), 386 (100, M^+), 368 (30), 284 (21), 256 (14), 221 (20), 220 (20), 207 (71), 206 (16), 205 (15), 189 (23), 180 (16), 175 (16), 167 (19), 166 (91), 165 (51), 161 (30), 147 (11), 139 (12), 138 (83), 137 (16), 129 (15), 122 (15), 77 (10), 73 (24), 71 (13), 69 (13), 60 (18), 57 (20), 55 (16); HRMS (EI) calcd for $C_{21}H_{22}O_7$ (M^+) 386.1366, found 386.1364.

hs-073 ((*E*)-1-(3,5-Dihydroxyphenyl)-5-(4-hydroxyphenyl)pent-4-ene-1,3-dione). Yield 46%; Orange powder; mp 118.3–119.4 °C; R_f 0.36 ($CHCl_3$/MeOH = 5/1); ^{1}H NMR (500 MHz, CD_3COCD_3) δ 8.99

(brs, 1H), 8.65 (brs, 2H), 7.66 (d, *J* = 16.0 Hz, 1H), 7.60–7.58 (m, 2H), 6.99 (d, *J* = 2.5 Hz, 2H), 6.92 (dt, *J* = 8.5, 2.5 Hz, 2H), 6.74 (d, *J* = 15.5 Hz, 1H), 6.59 (t, *J* = 2.5 Hz, 1H), 6.48 (s, 1H); ^{13}C NMR (126 MHz, CD_3COCD_3) δ 189.4, 181.9, 160.7, 159.8, 141.0, 139.4, 131.1, 127.8, 121.4, 116.9, 107.7, 106.7, 97.9; IR (KBr) 3394 s, 1631 s, 1592 s, 1547 m, 1514 m, 1453 m, 1344 s, 1302 m, 1214 w, 1173 s, 1006 m, 968 w, 822 m, 783 w, 661 w, 607 w; LRMS (EI, M = $C_{17}H_{14}O_5$) *m/z* 299 (13), 298 (70, M^+), 297 (15), 280 (10), 205 (14), 161 (62), 160 (16), 148 (13), 147 (100), 143 (11), 138 (92), 137 (64), 120 (38), 119 (28), 110 (35), 109 (19), 107 (14), 91 (23), 81 (10), 69 (34), 65 (14); HRMS (EI) calcd for $C_{17}H_{14}O_5$ (M^+) 298.0841, found 298.0838.

hs-089 ((*E*)-1-(3,4-Dimethoxyphenyl)-5-(3,5-dimethoxyphenyl)pent-4-ene-1,3-dione). Yield 43%; Yellow crystal; mp 108.6–109.5 °C (AcOEt); R_f 0.29 (hexane/AcOEt = 2/1); ^{1}H NMR (500 MHz, CD_3COCD_3) δ 16.36 (brs, 1H), 7.70 (dd, *J* = 8.5, 2.5 Hz, 1 H), 7.61–7.56 (m, 2H), 7.10 (d, *J* = 8.5 Hz, 1H), 6.92–6.86 (m, 3H), 6.64 (d, *J* = 2.5 Hz, 1H), 6.54 (t, *J* = 2.0 Hz, 1H), 3.92–3.91 (m, 6H), 3.84 (s, 6H); ^{13}C NMR (126 MHz, CD_3COCD_3) δ 190.9, 178.2, 162.3, 154.7, 150.4, 139.8, 138.3, 129.9, 124.9, 122.8, 111.9, 111.0, 106.7, 103.0, 98.3, 56.3, 56.2, 55.9; IR (KBr) 3437 w, 2936 w, 2837 w, 1634 m, 1597 s, 1515 m, 1464 m, 1269 s, 1207 m, 1165 m, 1065 w, 1025 w, 961 w, 823 w, 768 w, 691 w; LRMS (EI, M = $C_{21}H_{22}O_6$) *m/z* 371 (23), 370 (100, M^+), 353 (12), 352 (35), 301 (11), 206 (11), 205 (81), 204 (32), 191 (50), 190 (12), 166 (13), 165 (85), 151 (12), 148 (12), 77 (10), 69 (12), 57 (11); HRMS (EI) calcd for $C_{21}H_{22}O_6$ (M^+) 370.1416, found 370.1414.

hs-129 ((*E*)-5-(2,4-Dimethoxyphenyl)-1-(3,4-dimethoxyphenyl)pent-4-ene-1,3-dione). Yield 41%; Yellow powder, mp 139.4–140.2 °C (AcOEt); R_f 0.49 (hexane/AcOEt = 1/1); ^{1}H NMR (500 MHz, CD_3COCD_3) δ 16.6 (brs, 1H), 7.92 (d, *J* = 16.0 Hz, 1H), 7.68 (dd, *J* = 8.5, 2.0 Hz, 1H), 7.63 (d, *J* = 9.0 Hz, 1H), 7.60 (d, *J* = 2.0 Hz, 1H), 7.08 (d, *J* = 8.5 Hz, 1H), 6.80 (d, *J* = 15.0 Hz, 1H), 6.64–6.60 (m, 2H), 6.55 (s, 1H), 3.94 (s, 3H), 3.91–3.90 (m, 6H), 3.87 (s, 3H); ^{13}C NMR (126 MHz, CD_3COCD_3) δ 189.8, 180.1, 163.9, 160.7, 154.4, 150.3, 135.1, 130.5, 130.0, 122.4, 121.8, 117.8, 110.9, 106.9, 99.1, 97.4, 59.2, 56.09, 56.07, 55.9; IR (KBr) 3446 w, 2935 w, 2838 w, 1623 s, 1600 s, 1568 s, 1518 s, 1467 s, 1314 s, 1276 s, 1211 s, 1165 s, 1027 s, 974 m, 769 m, 676 w; LRMS (EI, M = $C_{21}H_{22}O_6$) *m/z* 370 (33, M^+),206 (12), 205 (88), 204 (17), 191 (72), 166 (16), 165 (100), 164 (13), 151 (35), 149

(15), 148 (15), 138 (10), 133 (12), 121 (13), 77 (16), 69 (12); HRMS (EI) calcd for $C_{21}H_{22}O_6$ (M^+) 370.1416, found 370.1412.

hs-140 ((*E*)-1,5-Bis(4-hydroxy-3-methoxyphenyl)pent-4-ene-1,3-dione). Yellow powder; mp 165.4–166.3 °C (AcOEt); R_f 0.29 (hexane/AcOEt = 1/1); ^{1}H NMR (500 MHz, CD_3COCD_3) δ 16.61 (brs, 1H), 8.39 (brs, 2H), 7.62–7.58 (m, 3H), 7.33 (d, *J* = 1.5 Hz, 1H), 7.17 (dd, *J* = 7.5, 2.0 Hz, 1H), 6.95 (d, *J* = 9.5 Hz, 1H), 6.89 (d, *J* = 9.0 Hz, 1H), 6.74 (d, *J* = 16.0 Hz, 1H), 6.55 (s, 1H), 3.94 (s, 3H), 3.93 (s, 3H); ^{13}C NMR (126 MHz, CD_3COCD_3) δ 189.9, 179.4, 152.2, 149.7, 148.4, 140.3, 129.2, 128.2, 123.6, 122.9, 121.4, 116.2, 115.7, 11.2, 111.0, 97.2, 56.3, 56.2; IR (KBr) 3397 m, 1631 m, 1593 s, 1517 s, 1468 m, 1437 m, 1310 m, 1262 s, 1215 s, 1176 m, 1127 m, 1035 m, 960 w, 835 w, 812 w, 790 w; LRMS (EI, M = $C_{19}H_{18}O_6$) *m/z* 343 (15), 342 (62, M^+), 324 (27), 191 (66), 190 (25), 177 (56), 152 (15), 151 (100), 150 (10), 145 (15); HRMS (EI) calcd for $C_{19}H_{18}O_6$ (M^+) 342.1103, found 342.1109.

hs-141 ((*E*)-1,5-Bis(3,4-dimethoxyphenyl)pent-4-ene-1,3-dione). Yield 44%; Pale orange crystal; mp 61.5–63.2 °C (MeOH); R_f 0.36 (hexane/AcOEt = 1/1); ^{1}H NMR (500 MHz, CD_3COCD_3) δ 16.56 (brs, 1H), 7.68 (dd, *J* = 8.5, 2.5 Hz, 1H), 7.63–7.59 (m, 2H), 7.33 (d, *J* = 2.0 Hz, 1H), 7.23 (dd, *J* = 8.0, 2.0 Hz, 1H), 7.09 (d, *J* = 8.5 Hz, 1H), 7.02 (d, *J* = 8.5 Hz, 1H), 6.78 (d, *J* = 15.0 Hz, 1H), 6.58 (s, 1H), 3.91–3.86 (m, 12H); ^{13}C NMR (126 MHz, CD_3COCD_3) 190.0, 179.3, 154.4, 152.2, 150.5, 150.2, 140.1, 129.8, 129.0, 123.4, 122.4, 121.9, 112.3, 111.7, 110.9, 110.7, 97.4, 56.1, 56.0; IR (KBr) 3428 w, 2938 w, 2837 w, 1632 s, 1588 m, 1510 m, 1461 m, 1436 m, 1356 w, 1266 s, 1163 m, 1023 m, 974 w, 853 w, 804 w, 766 w; LRMS (EI, M = $C_{21}H_{22}O_6$) *m/z* 371 (20), 370 (51, M^+), 353 (12), 352 (33), 206 (15), 205 (57), 204 (22), 191 (73), 190 (12), 166 (19), 165 (100), 151 (17); HRMS (EI) calcd for $C_{21}H_{22}O_6$ (M^+) 370.1416, found 370.1416.

hs-142 ((*E*)-5-(2,5-Dimethoxyphenyl)-1-(3,4-dimethoxyphenyl)pent-4-ene-1,3-dione). Yield 56%; Yellow needle; mp 165.4–166.3 °C (MeOH) R_f 0.49 (hexane/AcOEt = 1/1); ^{1}H NMR (500 MHz, CD_3COCD_3) δ 16.44 (brs, 1H), 7.96 (d, *J* = 16.5 Hz, 1H), 7.70 (dd, *J* = 8.5, 2.0 Hz, 1H), 7.61 (d, *J* = 2.0 Hz, 1H), 7.26 (d, *J* = 3.0 Hz, 1H), 7.09 (d, *J* = 8.5 Hz, 1H), 7.03 (d, *J* = 8.5 Hz, 1H), 6.99–6.92 (m, 2H), 6.62 (s, 1H), 3.91–3.89 (m, 9H), 3.81 (s, 3H); ^{13}C NMR (126 MHz, CD_3COCD_3) δ 190.7, 178.6,

154.6, 154.5, 153.5, 150.2, 134.4, 129.8, 125.2, 124.6, 122.6, 117.8, 113.5, 113.1, 111.7, 110.8, 98.1, 56.4, 56.1, 56.0, 55.9; IR (KBr) 3427 w, 2938 w, 2836 w, 1631 s, 1593 m, 1519 s, 1495 s, 1460 m, 1302 m, 1277 s, 1225 s, 1170 s, 1022 m, 975 w, 786 w, 717 w; LRMS (EI, M = $C_{21}H_{22}O_6$) *m/z* 370 (37, M^+), 339 (11), 205 (19), 204 (16), 191 (19), 176 (10), 166 (14), 165 (100); HRMS (EI) calcd for $C_{21}H_{22}O_6$ (M^+) 370.1416, found 370.1412.

hs-143 ((*E*)-5-(2,6-Dimethoxyphenyl)-1-(3,4-dimethoxyphenyl)pent-4-ene-1,3-dione). Yield 66%; Yellow crystal; mp 113.0–114.0 °C (MeOH); R_f 0.54 (hexane/AcOEt = 1/1); ^1H NMR (500 MHz, CD_3COCD_3) δ 16.73 (brs, 1H), 7.94 (d, *J* = 9.5 Hz, 1H), 7.55 (d, *J* = 15.5 Hz, 1H), 6.92–6.87 (m, 3H), 6.79 (s, 1H), 6.68–6.66 (m, 2H), 6.53 (t, *J* = 2.5 Hz, 1H), 3.98 (s, 3H), 3.90 (s, 3H), 3.83 (s, 6H); ^{13}C NMR (126 MHz, CD_3COCD_3) δ 188.7, 179.3, 165.6, 162.1, 161.8, 139.5, 138.2, 132.9, 125.5 118.8, 106.7, 106.6, 103.5, 102.8, 99.1, 56.2, 56.0, 55.7; IR (KBr) 3443 w, 2935 w, 2835 w, 1631 m, 1596 s, 1497 m, 1456 m, 1359 m, 1254 s, 1207 s, 1157 s, 1119 m, 1066 m, 1027 m, 970 m, 814 m; LRMS (EI, M = $C_{21}H_{22}O_6$) *m/z* 371 (21), 370 (59, M^+), 339 (12), 301 (26), 300 (13), 205 (20), 204 (15), 191 (29), 166 (10), 165 (100), 151 (21), 138 (14); HRMS (EI) calcd for $C_{21}H_{22}O_6$ (M^+) 370.1416, found 370.1416.

hs-144 ((*E*)-5-(2,3-Dimethoxyphenyl)-1-(3,4-dimethoxyphenyl)pent-4-ene-1,3-dione). Yield 60%; Yellow powder; mp 51.5–52.8 °C (MeOH); R_f 0.11 (hexane/AcOEt = 4/1); ^1H NMR (500 MHz, CD_3COCD_3) δ 16.39 (brs, 1H), 7.95 (d, *J* = 16.0 Hz, 1H), 7.71 (dd, *J* = 8.5, 2.0 Hz, 1H), 7.61 (d, *J* = 1.5 Hz, 1H), 7.33–7.31 (m, 1H), 7.14–7.08 (m, 3H), 6.90 (d, *J* = 16.5 Hz, 1H), 6.66 (s, 1H), 3.92–3.89 (m, 9H), 3.86 (s, 3H); ^{13}C NMR (126 MHz, CD_3COCD_3) δ 190.8, 178.1, 154.5, 154.1, 150.2, 149.1, 140.0, 129.7, 125.15, 125.05, 122.6, 119.4, 114.8, 111.6, 110.8, 98.2, 61.2, 56.1, 56.0; IR (KBr) 3438 w, 2935 w, 2835 w, 1632 m, 1582 m, 1516 m, 1464 m, 1427 m, 1267 s, 1224 m, 1165 m, 1068 w, 1022 w, 833 w, 792 w, 747 w; LRMS (EI, M = $C_{21}H_{22}O_6$) *m/z* 370 (32, M^+), 339 (22), 205 (15), 191 (17), 166 (12), 165 (100); HRMS (EI) calcd for $C_{21}H_{22}O_6$ (M^+) 370.1416, found 370.1411.

hs-145 ((*E*)-5-(2,6-Dimethoxyphenyl)-1-(3,4-dimethoxyphenyl)pent-4-ene-1,3-dione). Yield 77%; Yellow crystal; mp 137.5–138.9 °C (EtOH); R_f 0.11 (hexane/AcOEt = 4/1); ^1H NMR (500MHz,

CD_3COCD_3) δ 16.62 (brs, 1H), 8.14 (d, *J* = 16.0 Hz, 1H), 7.70 (dd, *J* = 8.5, 2.0 Hz, 1H), 7.60 (d, *J* = 3.0 Hz, 1H), 7.34 (t, *J* = 8.5 Hz, 1H), 7.26 (d, *J* = 16.0 Hz, 1H), 7.08 (d, *J* = 8.5 Hz, 1H), 6.73 (d, *J* = 8.5 Hz, 2H), 6.56 (s, 1H), 3.94 (s, 6H), 3.91–3.90 (m, 6H); ^{13}C NMR (126 MHz, CD_3COCD_3) δ 190.2, 180.4, 160.9, 154.3, 150.2, 132.2, 131.9, 130.0, 126.6, 122.4, 113.3, 111.7, 110.9, 104.8, 97.9, 56.3, 56.15, 56.07; IR (KBr) 3434 w, 2937 w, 2837 w, 1613 s, 1594 s, 1475 s, 1433 s, 1266 s, 1219 s, 1156 s, 1108 s, 1018 m, 873 m, 768 s, 614 w; LRMS (EI, M = $C_{21}H_{22}O_6$) *m/z* 370 (32, M$^+$), 339 (37), 191 (41), 166 (10), 165 (100), 151 (14); HRMS (EI) calcd for $C_{21}H_{22}O_6$ (M$^+$) 370.1416, found 370.1410.

hs-149 ((*E*)-1,5-Bis(4-hydroxy-3,5-dimethoxyphenyl)pent-4-ene-1,3-dione). Yield 83%; Orange crystal; mp 187.7–188.9 °C (MeOH); R_f 0.10 (hexane/AcOEt = 1/1); ^{1}H NMR (500 MHz, CD_3COCD_3) δ 16.62 (brs, 1H), 7.59 (d, *J* = 15.5 Hz, 1H), 7.38 (s, 2H), 7.03 (s, 2H), 6.73 (d, *J* = 15.5 Hz, 1H), 6.57 (s, 1H), 3.92–3.90 (m, 12H); ^{13}C NMR (126 MHz, CD_3COCD_3) δ 190.0, 179.3, 149.0, 148.6, 141.8, 140.7, 139.2, 127.8, 127.0, 121.5, 106.6, 106.1, 97.4, 56.7, 56.6; IR (KBr) 3507 m, 2996 w, 2941 w, 2838 w, 1632 m, 1607 m, 1514 s, 1463 m, 1343 m, 1285 m, 1210 m, 1149 m, 1110 s, 961 w, 818 w, 779 w; LRMS (EI, M = $C_{21}H_{22}O_8$) *m/z* 403 (16), 402 (51, M$^+$), 384 (30), 221 (25), 220 (27), 207 (50), 206 (11), 205 (15), 189 (22), 182 (41), 181 (100), 180 (12), 175 (23), 167 (15), 161 (27), 154 (28), 153 (12), 147 (15), 119 (13), 91 (11), 65 (11); HRMS (EI) calcd for $C_{21}H_{22}O_8$ (M$^+$) 402.1315, found 402.1314.

hs-150 ((*E*)-1-(2,4-Dimethoxyphenyl)-5-(3,4-dimethoxyphenyl)pent-4-ene-1,3-dione). Yield 88%; Yellow powder; mp 129.0–130.4 °C (EtOH); R_f 0.34 (hexane/AcOEt = 1/1); ^{1}H NMR (500 MHz, CD_3COCD_3) δ 16.89 (brs, 1H), 7.94 (d, *J* = 9.5 Hz, 1H), 7.59 (d, *J* = 15.5 Hz, 1H), 7.34 (s, 1H), 7.23 (dd, *J* = 8.5, 2.0 Hz, 1H), 7.01 (d, *J* = 8.5 Hz, 1H), 6.79–6.61 (m, 4H), 3.98 (s, 3H), 3.90–3.86 (m, 9H); ^{13}C NMR (126 MHz, CD_3COCD_3) δ 187.6, 180.8, 165.5, 161.7, 152.3, 150.6, 140.0, 132.8, 129.2, 123.4, 122.8, 118.9, 112.4, 111.1, 106.7, 102.9, 99.2, 56.2, 56.1, 56.1, 56.0; IR (KBr) 3421 w, 2929 w, 2834 w, 1619 s, 1573 s, 1522 s, 1458 s, 1293 s, 1254 s, 1208 s, 1134 s, 1073 w, 1026 s, 967 m, 818 m, 670 w; LRMS (EI, M = $C_{21}H_{22}O_6$) *m/z* 370 (22, M$^+$), 205 (13), 204 (13), 191 (31), 166 (10), 165 (100), 151 (20), 122 (11), 77 (14); HRMS (EI) calcd for $C_{21}H_{22}O_6$ (M$^+$) 370.1416, found 370.1414.

hs-151 ((*E*)-1,5-Bis(2,4-imethoxyphenyl)pent-4-ene-1,3-dione). Yield 67%; Yellow powder; mp 145.5–146.1 °C (EtOH); R_f 0.44 (hexane/AcOEt = 1/1); ^{1}H NMR (500 MHz, CD_3COCD_3) δ 16.95 (brs, 1H), 7.95–7.91 (m, 2H), 7.65 (d, J = 8.5 Hz, 1H), 6.78–6.59 (m, 6H), 3.98 (s, 3H), 3.94 (s, 3H), 3.90 (s, 3H), 3.87 (s, 3H); ^{13}C NMR (126 MHz, CD_3COCD_3) δ 187.2, 181.6, 165.4, 163.8, 161.7, 160.7, 134.9, 132.8, 130.2, 122.5, 118.9, 117.7, 106.9, 106.7, 102.8, 99.2, 99.0, 56.2, 56.1, 56.0, 55.9; IR (KBr) 3421 w, 2979 w, 2945 w, 2840 w, 1607 s, 1573 s, 1494 s, 1460 s, 1276 s, 1212 s, 1154 m, 1119 s, 1024 s, 975 m, 826 m, 637 w; LRMS (EI, M = $C_{21}H_{22}O_6$) *m/z* 370 (23, M$^+$), 339 (11), 205 (32), 204 (10), 191 (54), 166 (11), 165 (100), 151 (50), 148 (10), 77 (10); HRMS (EI) calcd for $C_{21}H_{22}O_6$ (M$^+$) 370.1416, found 370.1416.

hs-152 ((*E*)-1-(2,4-Dimethoxyphenyl)-5-(2,5-dimethoxyphenyl)pent-4-ene-1,3-dione). Yield 75%; Yellow needle; mp 88.5–89.9 °C (MeOH); R_f 0.53 (hexane/AcOEt = 1/1); ^{1}H NMR (500 MHz, CD_3COCD_3) δ 16.79 (brs, 1H), 7.98–7.93 (m, 2H), 7.28 (d, J = 3.0 Hz, 1H), 7.02 (d, J = 9.5 Hz, 1H), 6.97 (dd, J = 9.5, 3.0 Hz, 1H), 6.91 (d, J = 16.5 Hz, 1H), 6.77 (s, 1H), 6.68–6.66 (m, 2H), 3.99 (s, 3H), 3.90–3.88 (m, 6H), 3.81 (s, 3H), ^{13}C NMR (126 MHz, CD_3COCD_3) δ 188.3, 180.1, 165.6, 161.8, 154.7, 153.5, 134.3, 132.9, 125.4, 125.3, 119.0, 117.7, 113.5, 113.1, 106.8, 103.4, 99.2, 56.5, 56.2, 56.0; IR (KBr) 3435 w, 2939 w, 2833 w, 1624 s, 1604 s, 1493 s, 1463 s, 1329 m, 1253 s, 1212 s, 1177 m, 1111 m, 1020 s, 969 m, 796 m, 714 w; LRMS (EI, M = $C_{21}H_{22}O_6$) *m/z* 371 (13), 370 (45, M$^+$), 339 (17), 301 (11), 204 (11), 191 (20), 176 (10), 166 (10), 165 (100), 151 (13); HRMS (EI) calcd for $C_{21}H_{22}O_6$ (M$^+$) 370.1416, found 370.1414.

hs-153 ((*E*)-5-(2,3-Dimethoxyphenyl)-1-(2,4-dimethoxyphenyl)pent-4-ene-1,3-dione). Yield 81%; Yellow crystal (EtOH); mp 109.5–110.3 °C; R_f 0.53 (hexane/AcOEt = 1/1); ^{1}H NMR (500 MHz, CD_3COCD_3) δ 16.73 (brs, 1H), 7.96–7.92 (m, 2H), 7.34 (d, J = 6.5 Hz, 1H), 7.13–7.08 (m, 2H), 6.87 (d, J = 16.0 Hz, 1H), 6.80 (s, 1H), 6.68–6.67 (m, 2H), 3.99 (s, 3H), 3.90–3.86 (m, 9H); ^{13}C NMR (126 MHz, CD_3COCD_3) δ 188.5, 179.7, 165.6, 161.8, 154.2, 149.1, 133.9, 132.9, 129.9, 126.0, 236.0. 119.4. 118.9. 114.8. 106.8. 103.5. 99.2. 61.3. 56.2. 56.0; IR (KBr) 3435 w, 2933 w, 2834 w, 1632 m, 1601 s, 1579 s, 1459 s, 1329 m, 1262 s, 1216 s, 1119 m, 1072 m, 1027 m, 968 m, 798 m, 738 w; LRMS (EI, M = $C_{21}H_{22}O_6$) *m/z* 371 (10), 370 (33, M$^+$), 339 (22), 191 (16), 166 (10), 165 (100), 151(11);

HRMS (EI) calcd for $C_{21}H_{22}O_6$ (M^+) 370.1416, found 370.1413.

hs-154 ((*E*)-1-(2,4-Dimethoxyphenyl)-5-(2,6-dimethoxyphenyl)pent-4-ene-1,3-dione). Yield 83%; Yellow needle; mp 138.5–139.8 °C (EtOH); R_f 0.51 (hexane/AcOEt = 1/1); ^{1}H NMR (500 MHz, CD_3COCD_3) δ 16.95 (brs, 1H), 8.13 (d, *J* = 16.2 Hz, 1H), 7.94 (d, *J* = 8.5 Hz, 1H), 7.33 (t, *J* = 8.5 Hz, 1H), 7.21–7.17 (m, 1H), 6.73-6.66 (m, 5H), 3.99 (s, 3H), 3.94 (s, 6H), 3.90 (s, 3H); ^{13}C NMR (126 MHz, CD_3COCD_3) δ 187.5, 182.1, 165.4, 161.6, 160.9, 132.8, 132.2, 131.1, 127.4, 119.0, 113.3, 106.7, 104.8 103.0, 99.2, 56.3, 56.2, 56.0; IR (KBr) 3437 w, 2941 w, 2837 w, 1610 s, 1583 s, 1475 s, 1327 m, 1277 s, 1255 s, 1111 s, 1018 s, 984 m, 868 w, 820 m, 770 m, 725 m; LRMS (EI, M = $C_{21}H_{22}O_6$) *m/z* 371 (11), 370 (35, M^+), 340 (10), 339 (35), 301 (11), 191 (36), 166 (10), 165 (100), 151 (19); HRMS (EI) calcd for $C_{21}H_{22}O_6$ (M^+) 370.1416, found 370.1418.

(Figure 1-supplement.)

hs-127 ((*E*)-1-(3,4-Dimethoxyphenyl)-5-(4-hydroxy-3-methoxyphenyl)pent-4-ene-1,3-dione). Yield 41%; Orange powder; mp 138.5–139.4 °C (MeOH); R_f 0.31 (hexane/AcOEt = 1/1); ^{1}H NMR (500 MHz, CD_3COCD_3) δ 16.58 (brs, 1H) , 8.23 (brs, 1H), 7.68 (dd, *J* = 8.5, 2.0 Hz, 1H), 7.67–7.59 (m, 2H), 7.33 (d, *J* = 2.0 Hz, 1H), 7.18 (dd, *J* = 7.5, 2.0 Hz, 1H), 7.08 (d, *J* = 9.0 Hz, 1H), 6.74 (d, *J* = 15.5 Hz, 1H), 6.57 (s, 1H), 3.93–3.90 (m, 9H); ^{13}C NMR (126 MHz, CD_3COCD_3) δ 189.7, 179.7, 154.4, 150.2, 149.9, 148.8, 140.5, 129.8, 128.2, 123.6, 122.4, 121.4, 116.2, 111.8, 111.3, 110.9, 97.3, 56.3, 56.2, 56.1; IR (KBr) 3420 w, 2938 w, 2839 w, 1635 m, 1598 s, 1575 m, 1514 s, 1467 m, 1434 m, 1269 s, 1227 m, 1162 m, 1022 m, 967 w, 811 w, 784 w; LRMS (EI, M = $C_{20}H_{20}O_6$) *m/z* 357 (10), 356 (43, M^+), 338 (23), 191 (44), 190 (22), 177 (39), 166 (18), 165 (100), 145 (11); HRMS (EI) calcd for $C_{20}H_{20}O_6$ (M^+) 356.1260, found 356.1263.

hs-131 ((*E*)-1,5-Bis(3,4-dihydroxyphenyl)pent-4-ene-1,3-dione). Orange powder; mp 197.5–198.8 °C; R_f 0.29 ($CHCl_3$/MeOH = 9/1); ^{1}H NMR (500 MHz, CD_3COCD_3) δ 16.61 (brs, 1H), 8.76–

8.18 (m, 4H), 7.55–7.48 (m, 3H), 7.18 (d, *J* = 2.5 Hz, 1H), 7.07 (dd, *J* = 8.5, 2.0 Hz, 1H), 6.93 (d, *J* = 8.5 Hz, 1H), 6.88 (d, *J* = 8.5 Hz, 1H), 6.64 (d, *J* = 16.5 Hz, 1H), 6.48 (s, 1H); ^{13}C NMR (126 MHz, CD_3COCD_3) δ 189.8, 179.6, 150.9, 148.4, 146.3, 146.0, 140.2, 129.4, 128.5, 122.4, 121.6, 121.4, 116.0, 115.14, 115.07, 97.1; IR (KBr) 3393 m, 1636 s, 1600 s, 1577 s, 1541 s, 1520 s, 1473 m, 1446 s, 1313 s, 1294 s, 1193 m, 1166 s, 1112 m, 968 w, 848 w, 786 w; LRMS (EI, M = $C_{17}H_{14}O_6$) *m/z* 314 (28, M^+), 296 (18), 177 (42), 176 (23), 165 (14), 163 (40), 159 (13), 152 (10), 138 (15), 137 (100), 136 (14), 135 (12), 109 (13), 69 (12), 57 (12); HRMS (EI) calcd for $C_{17}H_{14}O_6$ (M^+) 314.0790, found 314.0790.

hs-158 ((*E*)-5-(2,3-Dimethoxyphenyl)-1-(2,5-dimethoxyphenyl)pent-4-ene-1,3-dione). Yield 54%; Pale orange crystal; mp 86.1–87.0 °C (MeOH/AcOEt); R_f 0.57 (hexane/AcOEt = 1/1); ^{1}H NMR (500 MHz, CD_3COCD_3) δ 16.47 (brs, 1H), 7.99 (d, *J* = 16.0 Hz, 1H), 7.44 (s, 1H), 7.36 (dd, *J* = 7.0, 2.0 Hz, 1H), 7.14–7.09 (m, 4H), 6.91 (d, *J* = 16.0 Hz, 1H), 6.81 (s, 1H), 3.93 (s, 3H), 3.89 (s, 3H), 3.87 (s, 3H), 3.81 (s, 3H); ^{13}C NMR (126 MHz, CD_3COCD_3) δ 189.0, 180.4, 154.4, 154.2, 154.0, 149.2, 134.7, 129.7, 126.6, 125.6, 125.1, 120.3, 119.4, 114.9, 114.9, 14.4, 104.0, 61.3, 56.6, 56.1, 55.9; IR (KBr) 3396 w, 2940 w, 2835 w, 1627 m, 1577 m, 1508 s, 1477 s, 1302 m, 1267 s, 1220 s, 1178 m, 1045 m, 1000 m, 808 m, 745 w, 628 w; LRMS (EI, M = $C_{21}H_{22}O_6$) *m/z* 371 (13), 370 (37, M^+), 340 (24), 339 (90), 191 (26), 176 (13), 174 (15), 166 (13), 165 (100); HRMS (EI) calcd for $C_{21}H_{22}O_6$ (M^+) 370.1416, found 370.1419.

hs-159 ((*E*)-5-(2,4-Dimethoxyphenyl)-1-(2,5-dimethoxyphenyl)pent-4-ene-1,3-dione). Yield 82%; Orange crystal; mp 129.0–130.3 °C (MeOH); R_f 0.54 (hexane/AcOEt = 1/1); ^{1}H NMR (500 MHz, CD_3COCD_3) δ 16.73 (brs, 1H), 7.97 (d, *J* = 15.0 Hz, 1H), 7.67 (d, *J* = 8.5 Hz, 1H), 7.44 (s, 1H), 7.10 (d, *J* = 3.0 Hz, 2H), 6.80 (d, *J* = 16.5 Hz, 1H), 6.73–6.70 (m , 1H), 6.64-6.60 (m, 2H), 3.95 (s, 3H), 3.92 (s, 3H), 3.87 (s, 3H), 3.81 (s, 3H); ^{13}C NMR (126 MHz, CD_3COCD_3) δ 187.4, 182.3, 164.0, 160.7, 154.4, 153.9, 135.7, 130.4, 126.7, 122.1 119.9, 117.5, 114.9, 114.4, 106.9, 103.4, 98.9, 56.6, 56.1, 55.9, 55.9; IR (KBr) 3340 w, 2944 w, 2833 w, 1619 m, 1599 m, 1502 s, 1456 m, 1299 m, 1276 s, 1206 m, 1152 m, 1035 m, 973 w, 822 m, 739 w, 630 w; LRMS (EI, M = $C_{21}H_{22}O_6$) *m/z* 371 (14), 370 (46, M^+), 340 (18), 339 (72), 321 (10), 205 (31), 192 (12), 191 (100), 176 (15), 174 (11), 166 (13), 165

(73), 151 (32), 149 (10), 148 (17), 133 (11), 121 (11), 77 (13); HRMS (EI) calcd for $C_{21}H_{22}O_6$ (M^+) 370.1416, found 370.1418.

hs-160 ((*E*)-1,5-Bis(2,5-dimethoxyphenyl)pent-4-ene-1,3-dione). Yield 68%; Yellow crystal; mp 62.1–63.4 °C (MeOH); R_f 0.53 (hexane/AcOEt = 1/1); ^{1}H NMR (500 MHz, CD_3COCD_3) δ 16.52 (brs, 1H), 8.01 (d, *J* = 16.5 Hz, 1H), 7.43 (s, 1H), 7.30 (d, *J* = 3.0 Hz, 1H), 7.12 (s, 2H), 7.04-6.93 (m, 3H), 6.78–6.75 (m, 1H), 3.92 (s, 3H), 3.89 (s, 3H), 3.811–3.805 (m, 6H); ^{13}C NMR (126 MHz, CD_3COCD_3) δ 188.8, 180.7, 154.6, 154.4, 154.0, 153.5, 135.0 126.7, 125.1, 124.9, 120.2, 118.0, 114.9, 114.4, 113.5, 113.0, 103.9, 56.6, 56.4, 55.9; IR (KBr) 3394 w, 2945 w, 2833 w, 1628 m, 1558 m, 1495 s, 1463 m, 1286 m, 1215 s, 1183 m, 1154 m, 1049 m, 971 w, 854 w, 804 m, 736 w; LRMS (EI, M = $C_{21}H_{22}O_6$) *m/z* 371 (12), 370 (31, M^+), 339 (51), 321 (10), 191 (29), 176 (19), 174 (12), 166 (17), 165 (100), 148 (10), 77 (11); HRMS (EI) calcd for $C_{21}H_{22}O_6$ (M^+) 370.1416, found 370.1413.

hs-161 ((*E*)-1-(2,5-Dimethoxyphenyl)-5-(2,6-dimethoxyphenyl)pent-4-ene-1,3-dione). Yield 52%; Yellow powder; mp 93.3–94.0 °C (MeOH); R_f 0.50 (hexane/AcOEt = 1/1); ^{1}H NMR (500 MHz, CD_3COCD_3) δ 16.70 (brs, 1H), 8.18–8.14 (m, 1H), 7.42 (dd, *J* = 8.5, 2.5 Hz, 1H), 7.35 (t, *J* = 8.5 Hz, 1H), 7.23–7.19 (m, 1H), 7.11 (d, *J* = 8.5 Hz, 2H), 6.74–6.68 (m, 3H), 3.94–3.93 (m, 9H), 3.81 (s, 3H); ^{13}C NMR (126 MHz, CD_3COCD_3) δ 187.8, 182.8, 160.9, 154.4, 153.8, 132.4, 131.9, 127.0, 126.8, 119.9, 114.9, 114.4, 113.1, 104.7, 103.6, 56.6, 56.3, 55.9; IR (KBr) 3423 w, 2958 w, 2832 w, 1616 m, 1586 m, 1499 s, 1475 s, 1321 w, 1258 m, 1215 s, 1109 s, 1043 m, 984 w, 868 w, 808 m, 773 m; LRMS (EI, M = $C_{21}H_{22}O_6$) *m/z* 370 (32, M^+), 340 (22), 339 (92), 191 (53), 176 (19), 174 (18), 166 (13), 165 (100), 151 (14), 149 (10), 148 (19), 133 (15), 122 (10), 120 (12), 107 (14), 105 (10), 91 (28), 77 (19); HRMS (EI) calcd for $C_{21}H_{22}O_6$ (M^+) 370.1416, found 370.1413.

hs-162 ((*E*)-1-(2,5-Dimethoxyphenyl)-5-(3,4-dimethoxyphenyl)pent-4-ene-1,3-dione). Yield 82%; Yellow powder; mp 130.7–131.3 °C (MeOH); R_f 0.26 (hexane/AcOEt = 2/1); ^{1}H NMR (500 MHz, CD_3COCD_3) δ 16.65 (brs, 1H), 7.63 (d, *J* = 15.5 Hz, 1H), 7.43 (s, 1H), 7.36 (s, 1H), 7.26–7.25 (m, 1H), 7.11 (s, 2H), 7.02 (d, *J* = 8.5 Hz, 1H), 6.81 (d, *J* = 16.5 Hz, 1H), 6.74–6.71 (m, 1H), 3.92 (s, 3H), 3.88-3.87 (m, 6H), 3.81 (s, 3H); ^{13}C NMR (126 MHz, CD_3COCD_3) δ 188.0, 181.4, 154.4, 153.9, 152.4,

150.5, 140.9, 128.9, 126.7, 123.7, 122.3, 120.0, 114.9, 114.4, 112.3, 111.0, 103.5, 56.6, 56.0, 55.9; IR (KBr) 3404 w, 2962 w, 2838 w, 1627 m, 1582 m, 1508 s, 1465 m, 1352 w, 1310 m, 1265 s, 1231 m, 1147 s, 1038 m, 975 m, 813 m, 733 w, 549 w; LRMS (EI, M = $C_{21}H_{22}O_6$) *m/z* 371 (12), 370 (30, M^+), 352 (10), 340 (11), 339 (34), 321 (15), 205 (19), 204 (12), 192 (10), 191 (73), 174 (11), 166 (25), 165 (100), 163 (18), 151 (29), 150 (13), 148 (17), 147 (12), 133 (15), 122 (19), 121 (10), 119 (14), 188 (12), 107 (22), 105 (11), 103 (10), 92 (14), 91 (21), 89 (10), 79 (15), 77 (29); HRMS (EI) calcd for $C_{21}H_{22}O_6$ (M^+) 370.1416, found 370.1413.

hs-163 ((*E*)-1-(2,5-Dimethoxyphenyl)-5-(3,5-dimethoxyphenyl)pent-4-ene-1,3-dione). Yield 67%; Yellow crystal; mp 94.8–95.6 °C (MeOH); R_f 0.40 (hexane/AcOEt = 2/1); ^{1}H NMR (500 MHz, CD_3COCD_3) δ 16.44 (brs, 1H), 7.60 (d, *J* = 16.5 Hz, 1H), 7.43 (s, 1H), 7.12 (s, 2H), 6.95–6.89 (m, 3H), 6.79 (s, 1H), 6.54 (d, *J* = 2.5 Hz, 1H), 3.92 (s, 3H), 3.84 (s, 6H), 3.81 (s, 3H); ^{13}C NMR (126 MHz, CD_3COCD_3) δ 189.2, 179.9, 162.1, 154.4, 154.0, 140.3, 138.0, 126.7, 125.2, 120.3, 114.9, 114.4, 106.7, 104.0, 103.0, 56.6, 55.9, 55.7; IR (KBr) 3005 w, 2939 w, 2836 w, 1635 m, 1589 s, 1531 s, 1455 s, 1333 m, 1279 s, 1204 s, 1162 s, 1047 s, 968 m, 845 m, 813 m, 719 w; LRMS (EI, M = $C_{21}H_{22}O_6$) *m/z* 371 (20), 370 (53, M^+), 340 (29), 339 (100), 205 (30), 204 (14), 191 (47), 166 (10), 165 (62), 148 (13); HRMS (EI) calcd for $C_{21}H_{22}O_6$ (M^+) 370.1416, found 370.1415.

Crystallographic data for **hs-163** is as follows: $C_{21}H_{22}O_6$, Mr = 370.40, 0.180 x 0.120 x 0.090 mm^3, triclinic, $P2_1/n$, a = 7.6061(2), b = 10.3452(3) , c = 12.1637(3) Å, α = 103.065(7) °, β = 101.093(7) °, γ = 99.231(7) °, V = 893.86(6) Å^3, Z = 2, ρ_c = 1.376 g/cm^{-3}, μ = 1.006 cm^{-1}, T = -150.0 ℃, λ = 0.71075 Å, 15315 reflections, 4085 unique [R(int) = 0.0131], Final GoF = 1.052, R_1 = 0.0387 ([$I > 2\sigma(I)$]), R_1 = 0.0421, wR_2 = 0.1093 (all data), Flack parameter = -0.002(4). CCDC No: 1900450.

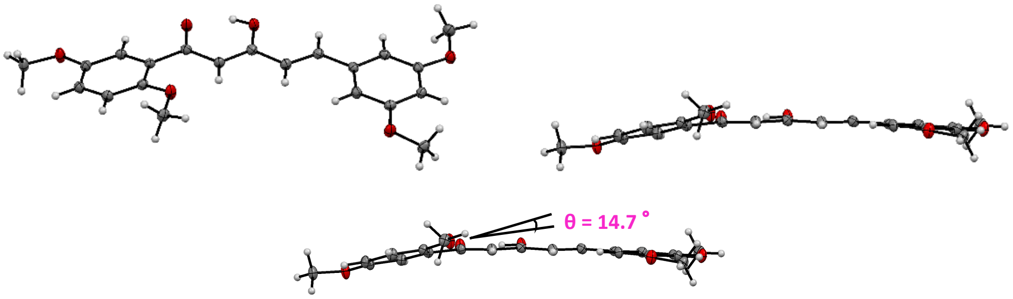

ORTEP representation of hs-163

HF-010 ((*E*) -5-(3,4-dimethoxyphenyl)-1-(3,5-Dimethoxyphenyl)pent-4-ene-1,3-dione). Yield 45%; Orange powder; mp 104.5–106.0 °C (MeOH); R_f 0.20 (hexane/AcOEt = 3/1) ^{1}H NMR (500 MHz, CD_3COCD_3) δ 16.49 (brs, 1H), 7.68 (d, *J* = 15.5 Hz, 1H), 7.34 (s, 1H), 7.25 (d, *J* = 7.5 Hz, 1H), 7.16 (d, *J* = 2.5 Hz, 2H), 7.02 (d, *J* = 8.5 Hz, 1H), 6.81 (d, *J* = 16.5 Hz, 1H), 6.72 (d, *J* = 2.0 Hz, 1H), 6.62 (s, 1H), 3.89–3.87 (m, 12H); ^{13}C NMR (126 MHz, CD_3COCD_3) δ 189.2, 181.4, 162.0, 152.4, 150.5, 141.1, 139.1, 128.8, 123.6, 121.8 ,112.3, 111.0, 105.8, 105.2, 98.0, 56.0, 55.9; IR (KBr) 3000 w, 2938 w, 2836 w, 1632 m, 1583 s, 1508 s, 1456 m, 1321 m, 1263 s, 1152 s, 1061 m, 1024 m, 968 m, 805 m, 692 w, 604 w; LRMS (EI, M = $C_{21}H_{22}O_6$) *m/z* 371 (12), 370 (48, M^+), 352 (14), 215 (12), 205 (43), 192 (12), 191 (100), 190 (20), 174 (11), 166 (79), 165 (35), 164 (10), 163 (14), 151 (18), 139 (11), 138 (71), 137 (15), 122 (12), 77 (11); HRMS (EI) calcd for $C_{21}H_{22}O_6$ (M^+) 370.1416, found 370.1419.

HF-014 ((*E*) -5-(2,4-dimethoxyphenyl)-1-(3,5-Dimethoxyphenyl)pent-4-ene-1,3-dione). Yield 48%; Orange needle; mp 122.0–123.0 °C (MeOH); R_f 0.32 (hexane/AcOEt = 2/1) ^{1}H NMR (500 MHz, CD_3COCD_3) δ 16.56 (brs, 1H), 7.97 (d, *J* = 16.0 Hz, 1H), 7.64 (d, *J* = 8.5 Hz, 1H), 7.16 (d, *J* = 1.5 Hz, 2H), 6.84 (d, *J* = 16.5 Hz, 1H), 6.71 (t, *J* = 2.5 Hz, 1H), 6.65–6.58 (m, 3H), 3.95 (s, 3H), 3.874-3.866 (m, 9H); ^{13}C NMR (126 MHz, CD_3COCD_3) δ 190.1, 181.9, 164.0, 162.0, 160.8, 139.3, 136.1, 130.7, 121.6, 117.4, 106.9, 105.8, 105.2, 99.0, 98.1, 56.1, 55.9; IR (KBr) 3419 w, 2939 w, 2834 w, 1631 m, 1604 m, 1570 m, 1501 w, 1456 m, 1296 m, 1206 m, 1157 m, 1045 m, 924 w, 827 w, 788 w, 635 w; LRMS (EI, M = $C_{21}H_{22}O_6$) *m/z* 371 (13), 370 (53, M^+), 339 (20), 205 (53), 192 (12), 191 (100), 176 (15), 166 (55), 165 (51), 164 (13), 151 (27), 149 (16), 148 (16), 138 (45), 137 (14), 133 (10), 122 (11), 121 (12), 77 (11); HRMS (EI) calcd for $C_{21}H_{22}O_6$ (M^+) 370.1416, found 370.1418.

HF-015 ((*E*) -5-(2,5-dimethoxyphenyl)-1-(3,5-Dimethoxyphenyl)pent-4-ene-1,3-dione). Yield 61%; Orange needle; mp 94.5–95.8 °C (MeOH/AcOEt); R_f 0.24 (hexane/AcOEt = 4/1) ^{1}H NMR (500 MHz, CD_3COCD_3) δ 16.36 (brs, 1H), 8.01 (d, *J* = 16.0 Hz, 1H), 7.27 (d, *J* = 2.5 Hz, 1H), 7.17 (d, *J* = 3.0 Hz, 2H), 7.05–6.96 (m, 3H), 6.73 (t, *J* = 2.5 Hz, 1H), 6.65 (s, 1H), 3.89 (s, 3H), 3.87 (s, 6H), 3.81 (s, 3H); ^{13}C NMR (126 MHz, CD_3COCD_3) δ 190.2, 180.5, 162.0, 154.6, 153.6, 139.2, 135.4, 125.0, 124.5, 118.0, 113.5, 113.2, 105.9, 105.4, 98.7, 56.4, 55.93, 55.87; IR (KBr) 3420 w, 2937 w, 2836 w, 1629 s, 1558 s, 1492 s, 1432 m, 1347 m, 1287 s, 1212 s, 1152 s, 1045 m, 965 m, 839 w, 782 m, 720 w;

LRMS (EI, M = $C_{21}H_{22}O_6$) *m/z* 371 (17), 370 (68, M^+), 340 (10), 339 (45), 205 (24), 201 (12), 191 (35), 176 (23), 166 (59), 165 (100), 151 (10), 148 (10), 139 (10), 138 (51), 137 (19), 122 (13), 77 (11), 69 (10); HRMS (EI) calcd for $C_{21}H_{22}O_6$ (M^+) 370.1416, found 370.1414.

HF-016 ((*E*)-5-(2,3-dimethoxyphenyl)-1-(3,5-Dimethoxyphenyl)pent-4-ene-1,3-dione). Yield 39%; Pale yellow needle; mp 139.5–140.4 °C (AcOEt/MeOH); R_f 0.24 (hexane/AcOEt = 4/1) ^{1}H NMR (500 MHz, CD_3COCD_3) δ 16.30 (brs, 1H), 8.00 (d, *J* = 16.0 Hz, 1H), 7.33 (dd, *J* = 7.5, 2.0 Hz, 1H), 7.18 (d, *J* = 2.5 Hz, 2H), 7.14–7.10 (m, 2H), 6.94 (d, *J* = 16.0 Hz, 1H), 6.73 (t, *J* = 2.0 Hz, 1H), 6.69 (s, 1H), 3.90 (s, 3H), 3.87 (s, 9H); ^{13}C NMR (126 MHz, CD_3COCD_3) δ 190.5, 180.1, 162.1, 154.2, 149.3, 139.2, 135.0, 129.6, 125.1, 120.0, 115.1, 105.9, 105.5, 98.9, 61.3, 56.2, 55.9; IR (KBr) 2945 w, 2838 w, 1635 s, 1582 s, 1481 s, 1353 s, 1308 s, 1271 s, 1206 s, 1157 s, 1047 m, 974 m, 924 w, 833 w, 786 m, 729 w; LRMS (EI, M = $C_{21}H_{22}O_6$) *m/z* 370 (34, M^+), 340 (11), 339 (49), 205 (19), 201 (10), 191 (28), 176 (16),174 (15), 166 (42), 165 (100), 138 (35), 137 (18), 122 (11), 77 (10); HRMS (EI) calcd for $C_{21}H_{22}O_6$ (M^+) 370.1416, found 370.1414.

Crystallographic data for **HF-016** is as follows: $C_{21}H_{22}O_6$, *M*r = 370.40, 0.080 x 0.010 x 0.010 mm^3, monoclinic, P-1, *a* = 4.03639(10), *b* = 20.9433(6), *c* = 21.1770(6) Å, , *β* = 92.340(7) °, *V* = 1788.71(9) Å^3, *Z* = 4, ρ_c = 1.375 g/cm^{-3}, μ = 1.005 cm^{-1}, *T* = -150.0 °C, *λ* = 0.71075 Å, 24755 reflections, 3260 unique [*R*(int) = 0.0785], Final *GoF* = 1.017, R_1 = 0.0448 ([$I > 2\sigma(I)$]), R_1 = 0.0728, wR_2 = 0.0948 (all data), Flack parameter = -0.002(4). CCDC No: 1900446.

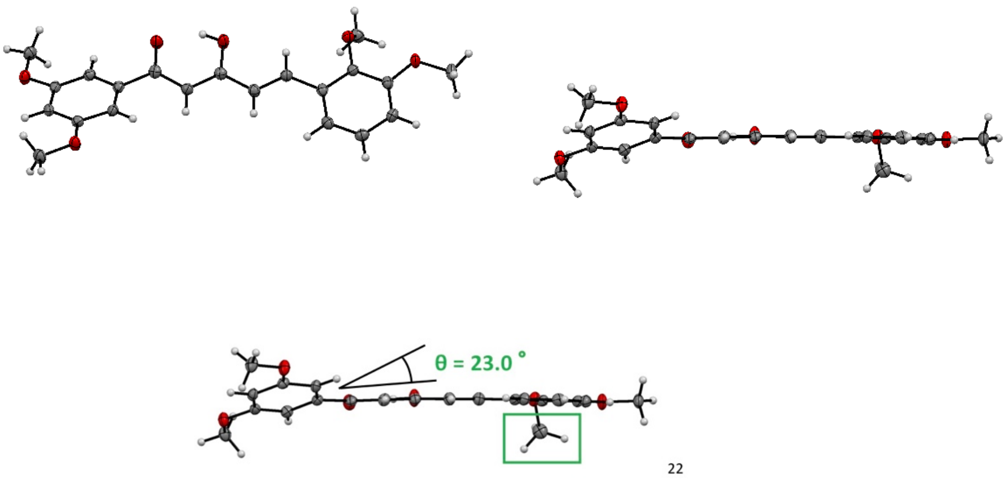

ORTEP representation of **HF-016**

HF-017 ((*E*) -5-(2,6-dimethoxyphenyl)-1-(3,5-Dimethoxyphenyl)pent-4-ene-1,3-dione). Yield 72%; Yellow powder; mp 121.0–122.4 °C (EtOH); R_f 0.26 (hexane/AcOEt = 4/1) ^{1}H NMR (500 MHz, CD_3COCD_3) δ 16.56 (brs, 1H), 8.19 (d, *J* = 16.0 Hz, 1H), 7.35 (t, *J* = 8.5 Hz 1H), 7.29 (d, *J* = 16.5 Hz, 1H), 7.17 (d, *J* = 2.0 Hz, 2H), 6.74–6.71 (m, 3H), 6.59–6.58 (m, 1H), 3.94 (s, 6H), 3.87 (s, 6H); ^{13}C NMR (126 MHz, CD_3COCD_3) δ 189.6, 182.2, 162.0, 161.0, 139. 4, 132.5, 132.0, 126.4, 113.1, 105.8, 105.2, 104.8, 98.6, 56.3, 55.9; IR (KBr) 3004 w, 2939 w, 2838 w, 1623 s, 1569 s, 1472 s, 1430 s, 1345 s, 1302 s, 1207 s, 1155 s, 1112 s, 1040 m, 986 w, 834 w, 772 m; LRMS (EI, M = $C_{21}H_{22}O_6$) *m/z* 371 (16), 370 (30, M^+), 340 (33), 339 (69), 338 (16), 201 (10), 191 (66), 176 (13), 166 (13), 165 (100), 151 (29), 149 (12), 148 (13), 138 (15), 137 (14); HRMS (EI) calcd for $C_{21}H_{22}O_6$ (M^+) 370.1416, found 370.1416.

Figure S4 – Related to Figure 7

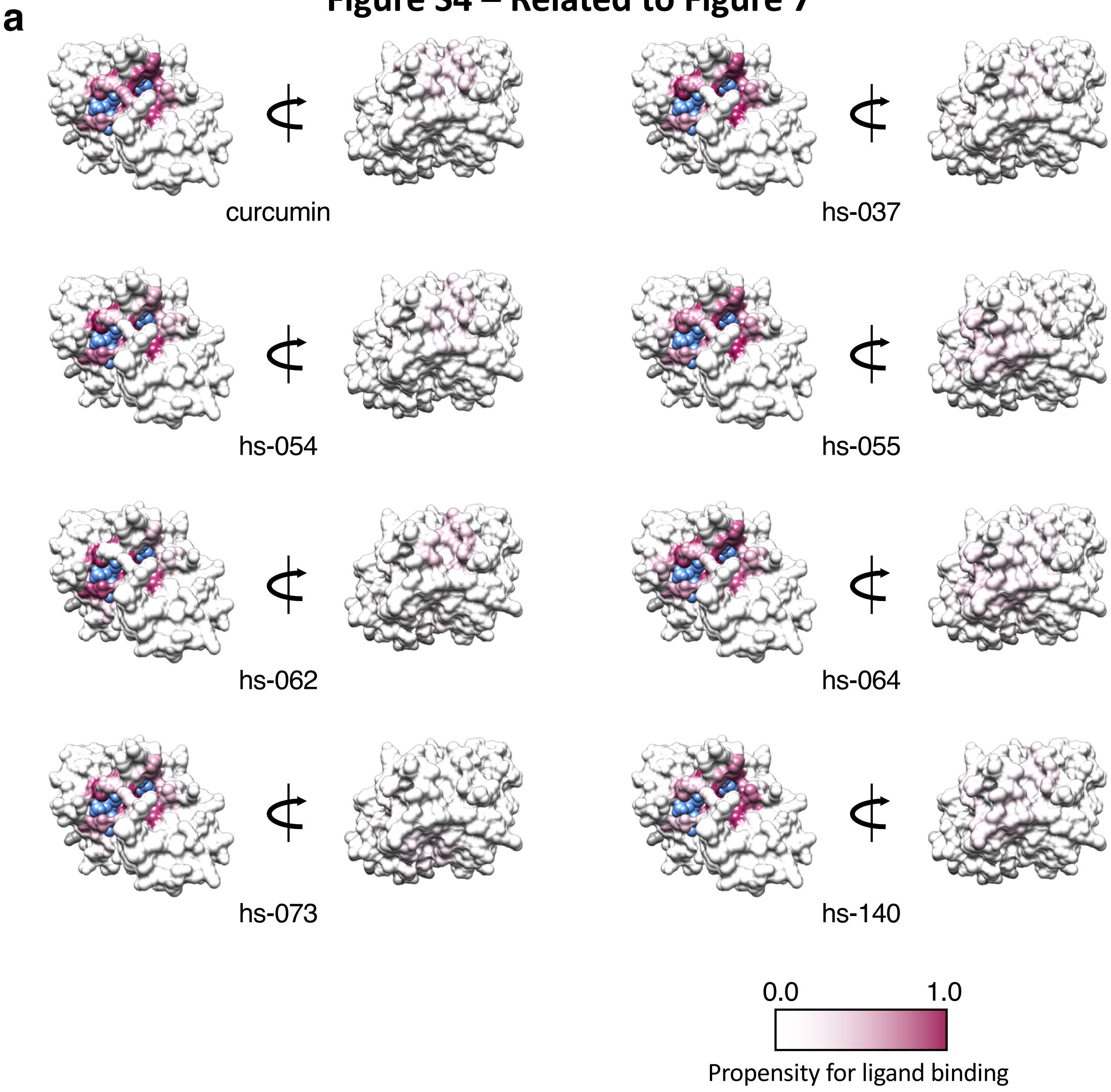

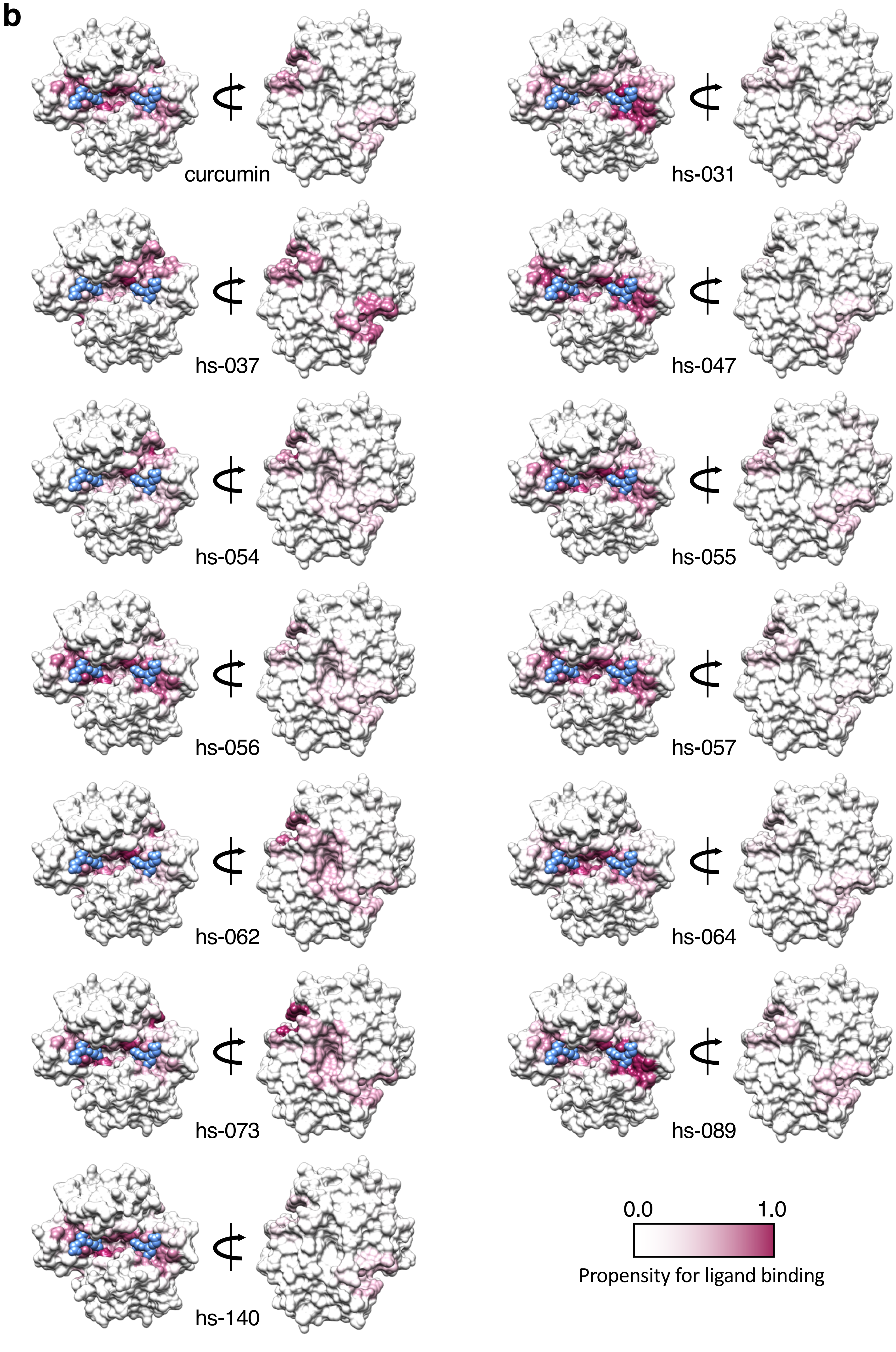
b
curcumin
hs-031
hs-037
hs-047
hs-054
hs-055
hs-056
hs-057
hs-062
hs-064
hs-073
hs-089
hs-140
0.0
1.0
Propensity for ligand binding

c

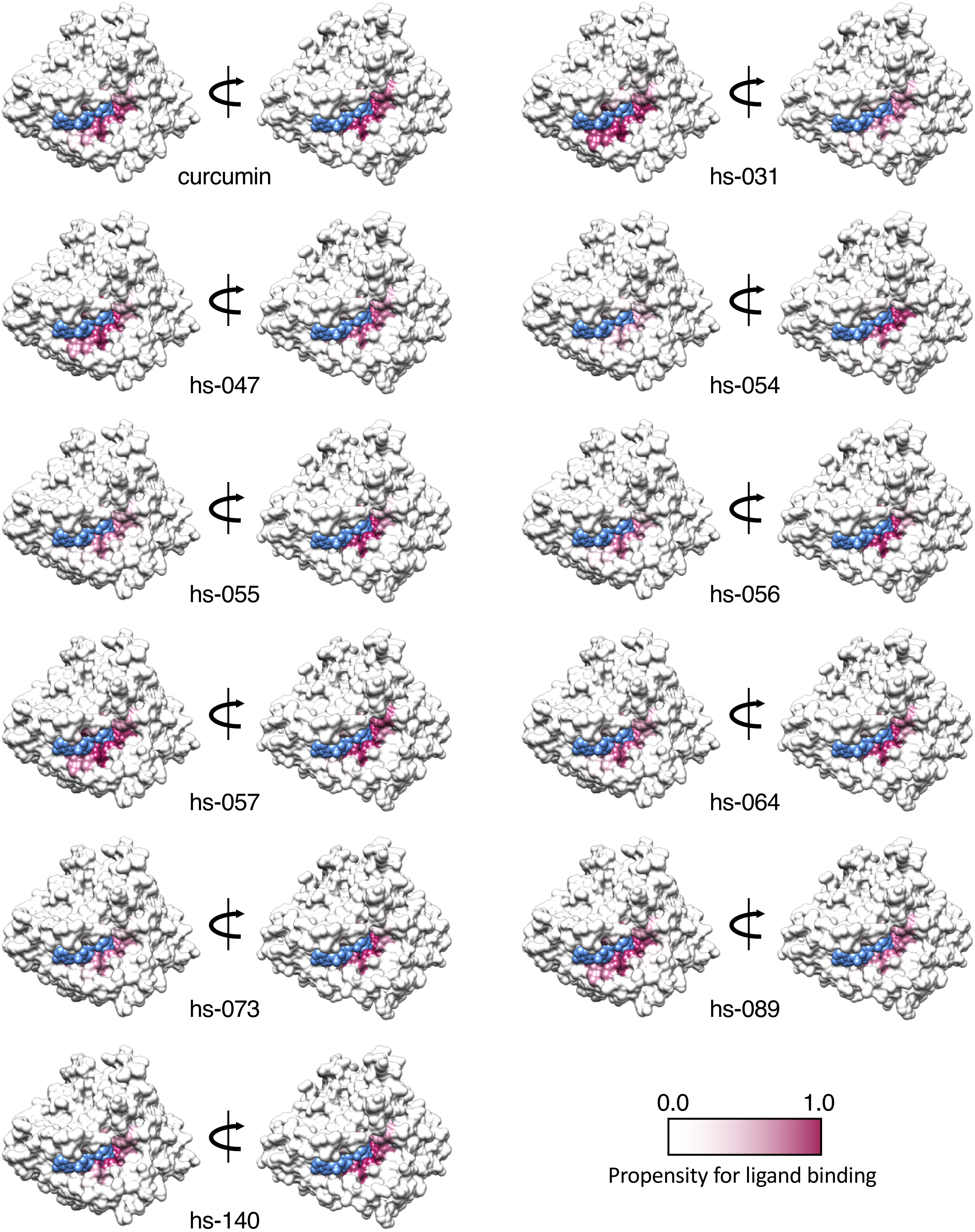
curcumin
hs-031
hs-047
hs-054
hs-055
hs-056
hs-057
hs-064
hs-073
hs-089
hs-140
0.0
1.0
Propensity for ligand binding

d

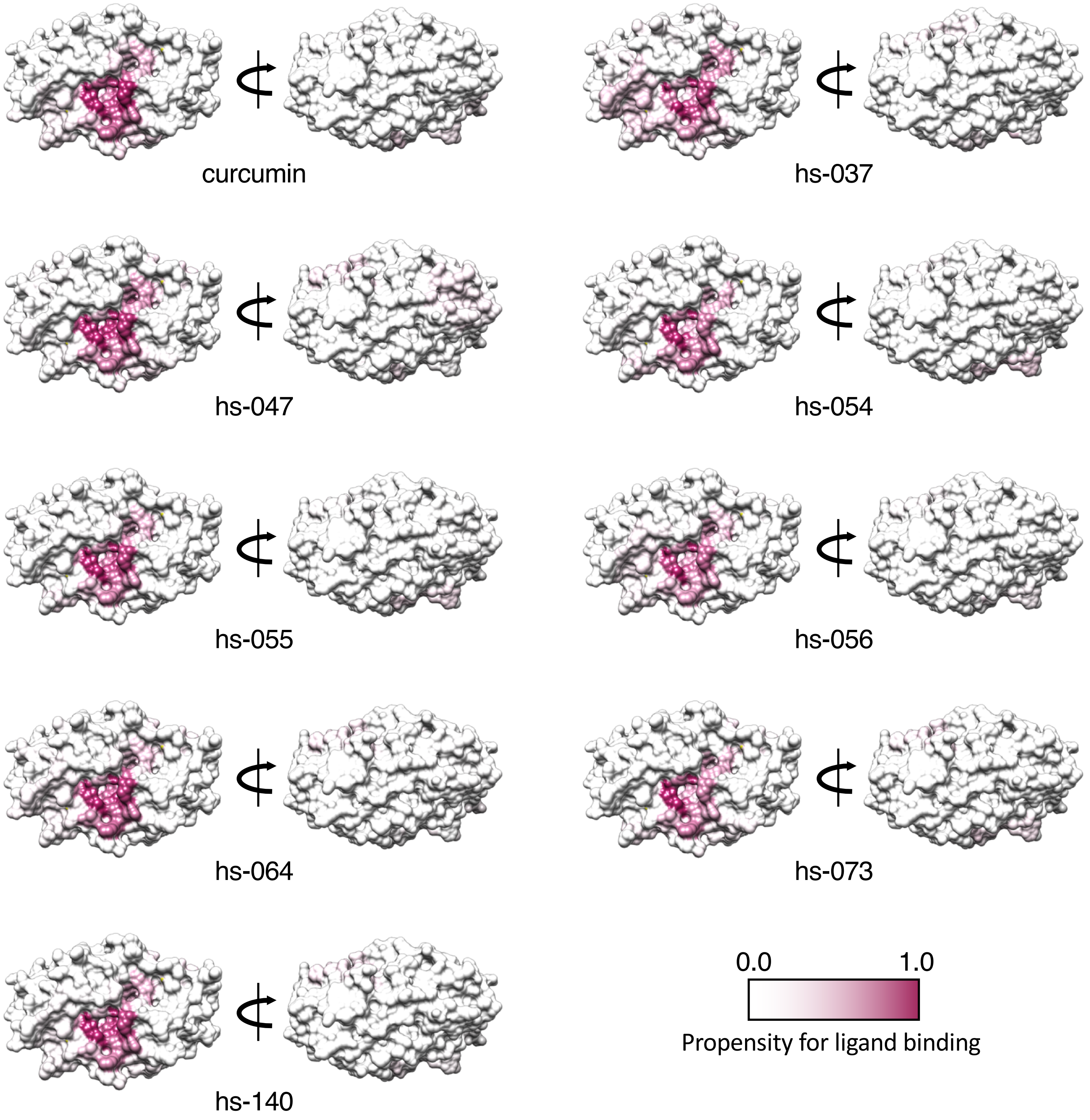
curcumin
hs-037
hs-047
hs-054
hs-055
hs-056
hs-064
hs-073
hs-140
0.0
1.0
Propensity for ligand binding

e

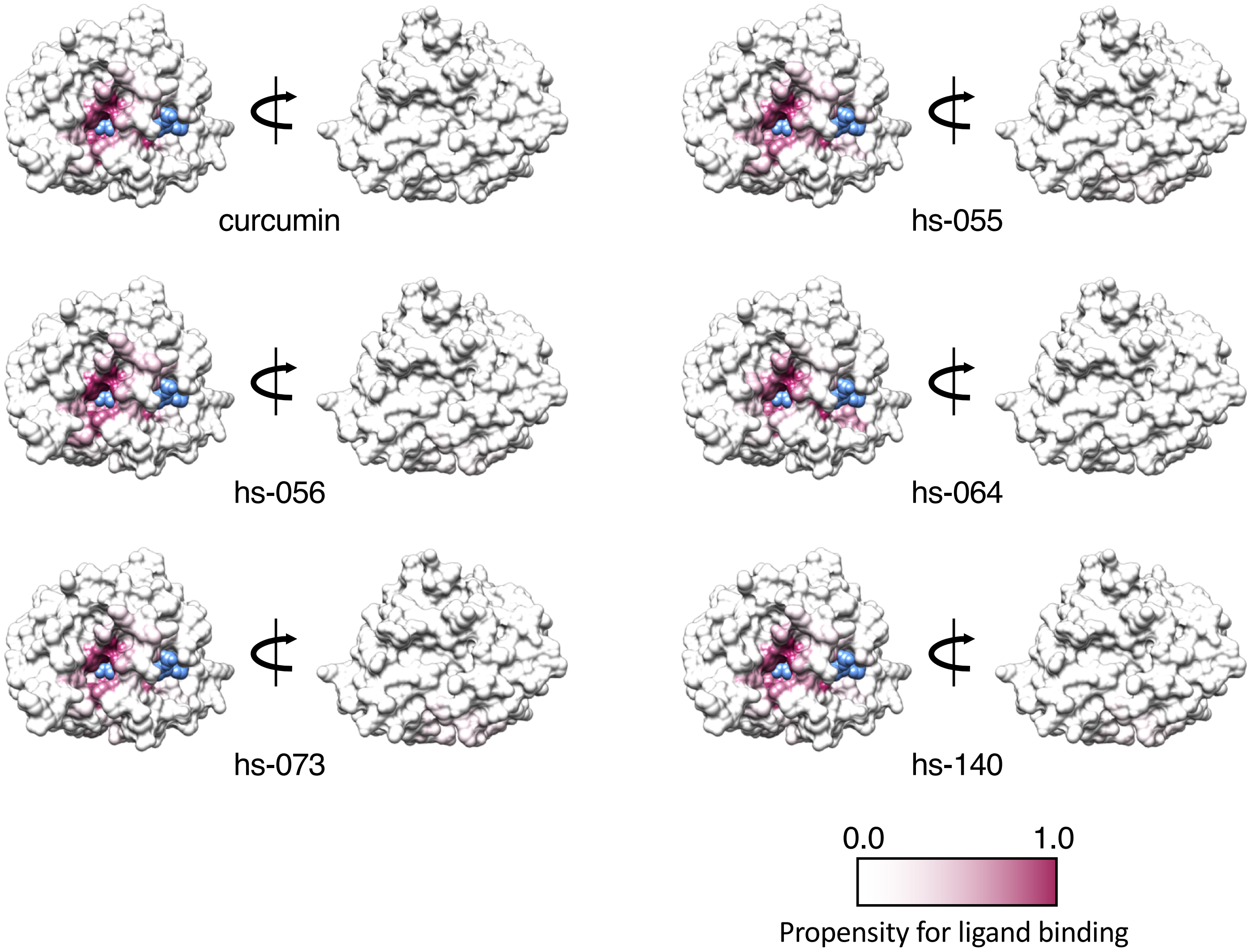
curcumin
hs-055
hs-056
hs-064
hs-073
hs-140
0.0
1.0
Propensity for ligand binding

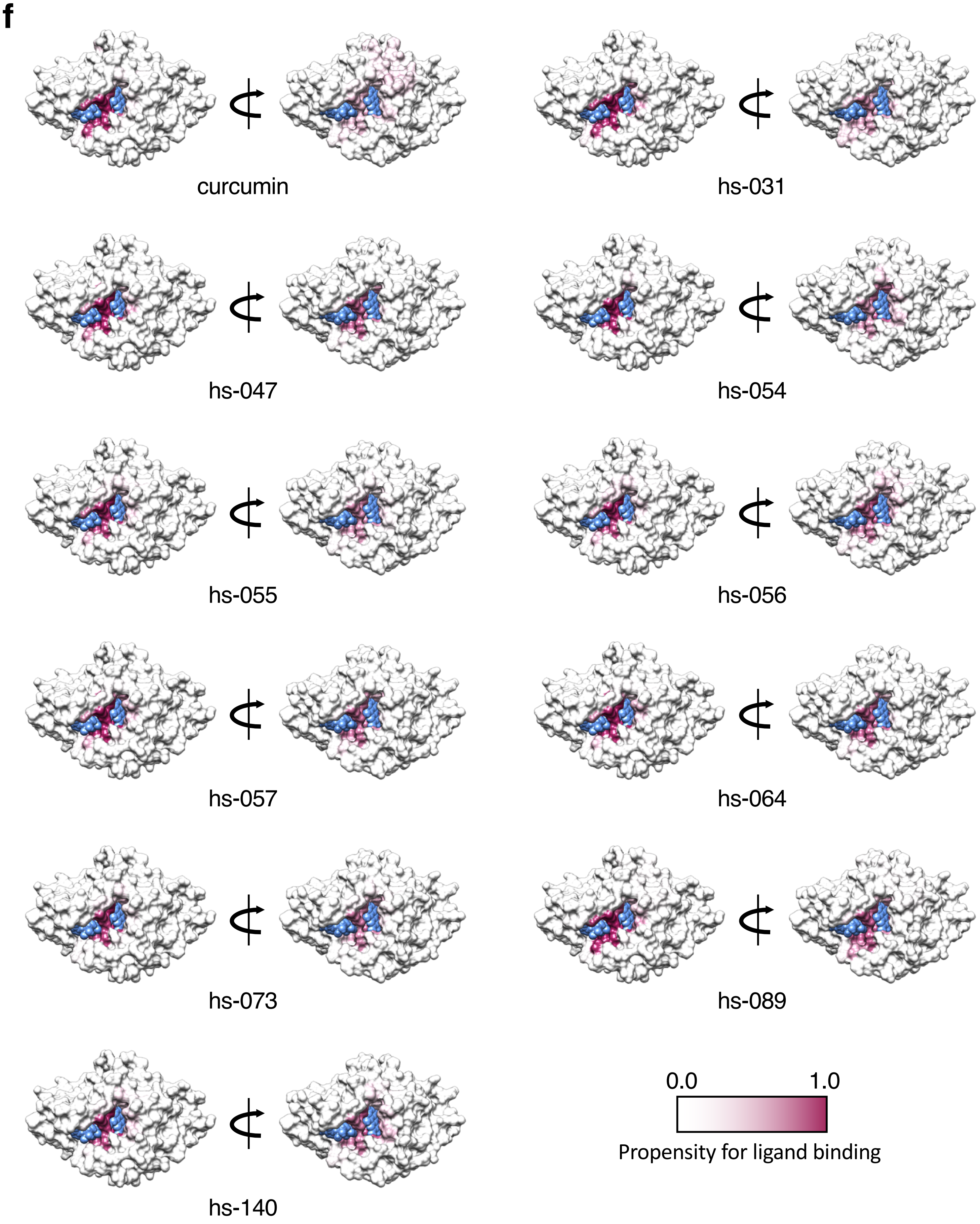
f
curcumin
hs-031
hs-047
hs-054
hs-055
hs-056
hs-057
hs-064
hs-073
hs-089
hs-140
0.0
1.0
Propensity for ligand binding

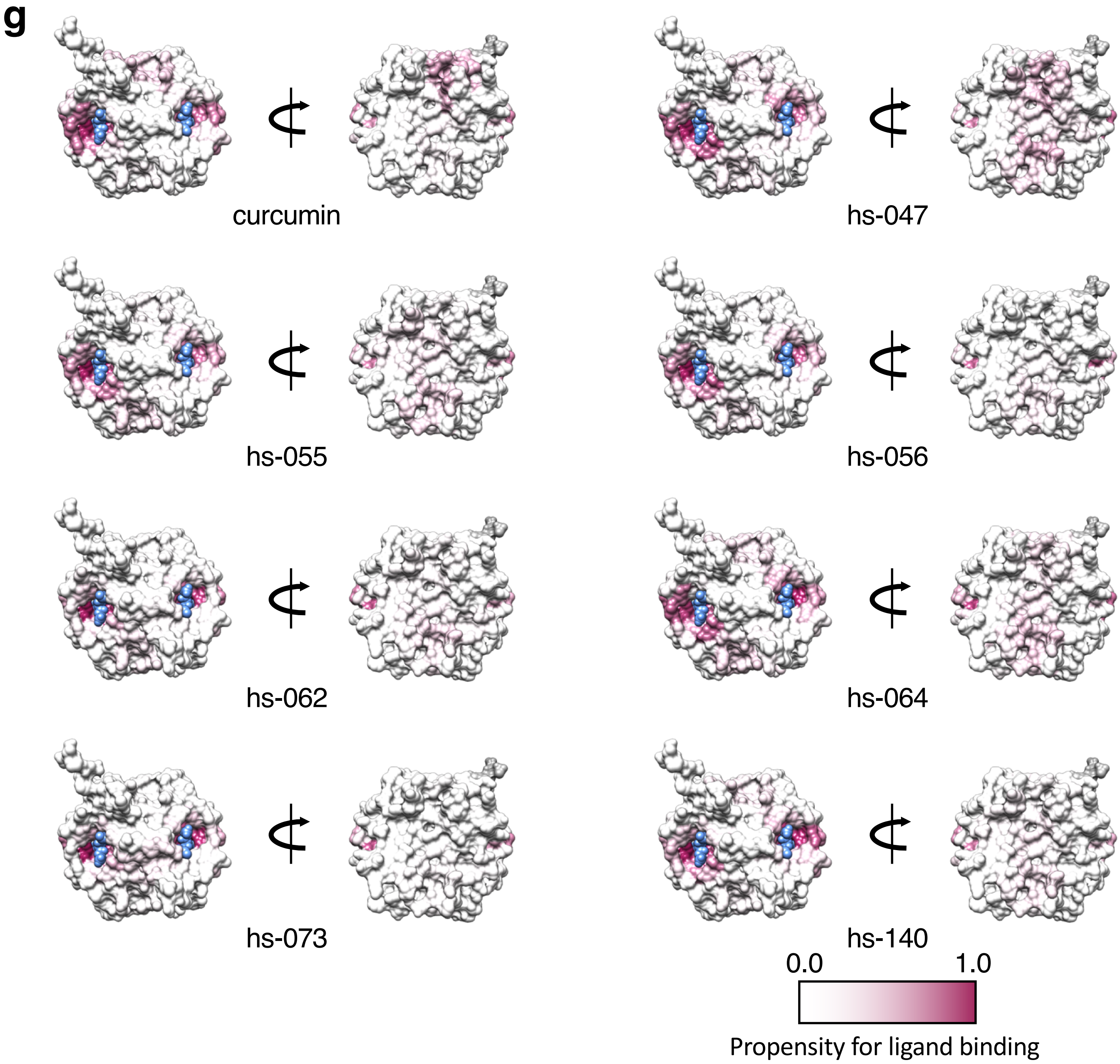
g
curcumin
hs-047
hs-055
hs-056
hs-062
hs-064
hs-073
hs-140
0.0
1.0
Propensity for ligand binding

Supplementary Table S1

	CLogP	Growth (allowed)	Death (induced)	GI50	Washout growth (allowed)	b-gal (Day 2)	b-gal (Day 4)	ROS (1h, fold)	ROS (24h, fold)	ROS (72h, fold)	Tumor formation(%)
Curcumin	2.9394	5.17	10.8	13.3	10	3.67	25	0.325	2.38	1.34	0
hs-031	4.59	2.17	11.8	10	9.67	1	14	1.03	1.53	1.89	59.6774194
hs-037	1.91	1.67	15	3.26	2	4	10.3	5.27	3.27	4.21	4.83870968
hs-047	4.03	12.2	2.17	13.6	129	4	73	1.1	1.12	1.43	279.290323
hs-054	1.81	6.83	8	11.9	14.8	2.67	16.3	3.31	1.25	1.66	89.8387097
hs-055	3.2	3	8.33	21.8	1	2.33	6.33	1.4	1.14	0.92	30.6451613
hs-056	3.35	33.8	1.33	37.3	128	5	32	4.43	6.04	1.83	14.516129
hs-057	3.32	8	4.33	8.78	51.2	0	24	0.994	1.23	1.29	3.22580645
hs-062	1.88	4.67	1.33	17.2	54.5	0.33	33	0.604	0.715	0.851	201.612903
hs-064	2.98	5.5	2.33	12.5	6.17	0.67	5.33	0.75	1.43	0.899	17.7419355
hs-073	2.47	30.7	0.5	38	131	6.67	37	2.37	2.85	1.01	82.2580645
hs-089	3.68	7.5	2.83	9.56	27.8	1.33	10.67	1.02	0.983	0.674	61.2903226
hs-127	2.85			16							
hs-129				14.8		6.5	3.25	1.32	1.029		
hs-131	1.95	3.5	9.66	8.56							
hs-135	2.31			11.3							
hs-140	2.65	1.5	4.83	15.7	18	57.5	12.49	0.643	1.06	1.65	306.451613
hs-141				13		0.96	23.02	0.5532	1.158		
hs-142				12.9		0.79	3.24	2.058	1.129		
hs-143				18.3	69	4.95	77.64	1.1459	1.228		
hs-144				21.2		0.42	14.4	0.5036	0.91		
hs-145		6.67	10.67	8.68		1.31	19.88	1.202	0.942		
hs-149	2.16			13.8	7.2						
hs-150				42.5	271	9.78	33.12	1.22	1.421		
hs-151				24	15	3.88	15.13	0.685	1.309		
hs-152				27.7	98	4.91	70.99	1.4818	1.297		
hs-153				24.7	7	5.31	27.43	0.969	1.218		
hs-154				29.6	233	0.59	18.58	0.9297	1.176		
hs-157	4.77			7.74		0.35	3.95	0.3213	1.015		50
hs-158	3.68			15.8							
hs-159	4.03	11.2	8	8.54							
hs-160	4.03	4.67	9.33	5.8							116.129032
hs-161	4.03			10.3							
hs-162	3.68			12.2							
hs-163	4.03	3	11	3.84							11.2903226
HF-010	3.68	7.67	7.66	4.53							
HF-014	4.03			15.5							
HF-015	4.03			19.33							
HF-016	3.68			101.12							
HF-017	4.03			11.56							
